# A deep transfer learning based convolution neural network framework for air temperature classification using human clothing images

**DOI:** 10.1038/s41598-024-80657-y

**Published:** 2024-12-30

**Authors:** Maqsood Ahmed, Xiang Zhang, Yonglin Shen, Nafees Ali, Aymen Flah, Mohammad Kanan, Mohammad Alsharef, Sherif S. M. Ghoneim

**Affiliations:** 1 School of Geography and Information Engineering, China University of Geosciences, Wuhan, 430074 China; 2 National Engineering Research Center of Geographic Information System, China University of Geosciences, Wuhan, 430074 China; 3 State Key Laboratory of Geomechanics and Geotechnical Engineering, Institute of Rock and Soil Mechanics, Chinese Academy of Sciences, Wuhan, 430071 China; 4 University of Chinese Academy of Sciences, Beijing, 100049 China; 5 National Engineering School of Gabès, University of Gabès, 6072 Gabès, Tunisia; 6 Centre for Research Impact & Outcome, Chitkara University Institute of Engineering and Technology, Chitkara University, Rajpura, 140401, Punjab, India; 7 Chitkara Centre for Research and Development, Chitkara University, Baddi, Himachal Pradesh, 174103, India; 8 Applied Science Research Center, Applied Science Private University, Amman, 11931 Jordan; 9 VSB-Technical University of OSTRAVA, Ostrava, Czech Republic; 10 Industrial Engineering Department, College of Engineering, University of Business and Technology (UBT), 21448 Jeddah, Saudi Arabia; 11 Department of Electrical Engineering, College of Engineering, Taif University, P.O. BOX 11099, 21944 Taif, Saudi Arabia; 12 College of Engineering, University of Business and Technology (UBT), Jeddah, 21448, Saudi Arabia

**Keywords:** Air temperature, Human clothing, Deep transfer learning, Classification, Computational biology and bioinformatics, Climate sciences, Environmental sciences

## Abstract

Weather recognition is crucial due to its significant impact on various aspects of daily life, such as weather prediction, environmental monitoring, tourism, and energy production. Several studies have already conducted research on image-based weather recognition. However, previous studies have addressed few types of weather phenomena recognition from images with insufficient accuracy. In this paper, we propose a transfer learning CNN framework for classifying air temperature levels from human clothing images. The framework incorporates various deep transfer learning approaches, including DeepLabV3 Plus for semantic segmentation and others for classification such as BigTransfer (BiT), Vision Transformer (ViT), ResNet101, VGG16, VGG19, and DenseNet121. Meanwhile, we have collected a dataset called the Human Clothing Image Dataset (HCID), consisting of 10,000 images with two categories (High and Low air temperature). All the models were evaluated using various classification metrics, such as the confusion matrix, loss, precision, F1-score, recall, accuracy, and AUC-ROC. Additionally, we applied Gradient-weighted Class Activation Mapping (Grad-CAM) to emphasize significant features and regions identified by models during the classification process. The results show that DenseNet121 outperformed other models with an accuracy of 98.13%. Promising experimental results highlight the potential benefits of the proposed framework for detecting air temperature levels, aiding in weather prediction and environmental monitoring.

## Introduction

The prediction of weather phenomena significantly impacts several domains, including environmental monitoring, weather prediction, and the evaluation of environmental quality^1^. Both natural processes and human activities contribute to global climate change, resulting in extended alterations in weather patterns^2^. Many climate events such as snow, sandstorms, and haze exert substantial influence on autonomous driving systems^3^, and also have profound effects on our daily routines, travel patterns, and choice of attire^4^. Severe weather conditions pose significant hazards to traffic, leading to accidents on highways caused by intense rainfall, snow showers, thick fog and sandstorms^5,6^. Furthermore, weather conditions also can have diverse effects on agriculture process^7^. The contemporary weather phenomena can influence the weather conditions of subsequent days^8^. Additionally, the temperature fluctuations have caused extreme climate challenges^9^. The rising air temperatures contribute to climate change alongside with several other factors, leading to extensive consequences including rise seawater-level rising, raised rate of severe events, and increased global warming^10^. The extreme hot and cold temperatures have caused in critical impacts on mortality rates^11^. Furthermore, intense health concerns in populations have been linked with departures from the suitable range of air temperatures^12,13^. The existing research has discovered that there exists a link between air temperature and daily fatalities in metropolitan areas^14^. Air temperature is an essential factor in climate change as it directly impacts atmospheric processes, weather patterns, and ecosystems. Therefore, predicting and understanding air temperature is important in order to address the impacts of climate change.

Meteorologists typically utilize thermometers, satellite imagery, and weather forecast models to measure air temperature. The estimating temperatures over diverse locations commonly involves collecting large-scale data and using complex algorithms, potentially compromising accuracy. Moreover, fluctuating environmental conditions and inadequate sensor coverage can supplementary challenge the precision of these estimates. Artificial intelligence (AI) is revolutionizing various domains, including computer vision, remote sensing and environmental science, by enabling more accurate climate modeling, wildlife monitoring, and natural resource management. The rapid progress of machine learning in AI, nowadays allows researchers to actively employ it across various academic fields^15^ proposed a method based on and Least Squares Support Vector Machine and Empirical Mode Decomposition for air temperature prediction.^16^ developed an Xgboost model to accurately estimate the outdoor air temperature. Besides, Ref.^17^ proposed an innovative nonparametric technique based on random forest to estimate outdoor temperatures. The model employed data gathered at NUS’s Kent Ridge campus from February to July 2019. However, ordinary machine learning faces difficulties in accurately acquiring the characteristics of weather phenomena for weather phenomenon recognition. Deep learning methods revolutionizes machine learning by utilizing complex neural networks to process huge amounts of data, achieving supreme accuracy in jobs like image recognition and natural language processing^18^.

The Convolutional Neural Network (CNN) is a deep learning method renowned for its prowess in representing image features due to its utilization of a deep model structure with convolutional kernels, pooling operations, and other relevant mechanisms^19,20^. The CNN, pioneered by AlexNet’s success in the ImageNet Large-Scale Visual Recognition Challenge (ILSVRC), has become a foundational model across various research domains such as continuous prediction^21^, object detection^22^, and face recognition^23^. Recently, many researchers have been applying CNN models to atmospheric issues. Reference^24^ employed CNNs to perform snow cover extraction from earth observation data. Two models were developed based on CNNs, demonstrating improved accuracy in cloud recognition tasks^25^. A framework with a lightweight deep structure was proposed, integrating daytime and nighttime image segmentation. This approach was applied to public databases, resulting in improved results^26^. In conclusion, it can be seen that CNNs are preferred and offer distinct advantages in meteorological research and the classification of weather phenomena. Recently, several studies have employed CNNs for weather phenomena classification, achieving the highest accuracy and demonstrating effective performance^27^. These studies have successfully addressed tasks such as sunny and cloudy classification^4^ and the classification of weather into three classes: rainy, foggy, and snowy^28^. Reference^29^ successfully developed a deep classification model capable of accurately classifying six classes of weather events, encompassing rain, snow, haze, frozen, dust and dew. Another study effectively utilized a three-channel CNN for the classification of weather phenomena into six classes^30^. Besides, Ref.^31^ developed a multi-classification model for weather phenomena recognition. However, these studies only focus on a limited number of weather phenomena and lack accuracy. There exists a wide array of weather conditions in our daily lives, each varying significantly. Therefore, it is essential to incorporate additional types of weather phenomena for analysis and recognition.

To the best of our knowledge, previous research has not extensively addressed the prediction of air temperature based on human clothing images using deep learning techniques. As we know that, Humans efficiently adjust their clothing on their bodies in response to temperature conditions to achieve optimal thermal comfort^32^. Furthermore, clothing serves as a portable environment, allowing individuals to adapt to various settings, indoors or outdoors, by regulating their microclimate economically and sustainably^33,34^. Therefore, in this study, we utilized images of human clothing to classify air temperature levels into two categories: high temperature and low temperature, employing a deep transfer learning convolutional neural network framework. Our research represents a significant advancement in the field of weather recognition, offering a novel, precise, and practical solution for classifying air temperature based on images of human clothing. This method holds promise for applications in diverse domains such as weather prediction, environmental monitoring, tourism, building management systems, and energy production.

In this paper, we present a deep transfer learning framework designed for the classification of low and high-level temperatures using human clothing images. Secondly, we have created a Human Clothing Image Dataset (HCID) consisting of 10,000 images, with each category comprising 5000 images. Thirdly, we apply transfer learning techniques on customizing proposed models, and evaluate the performance with several metrics. Additionally, we employ the Grad-CAM test to visualize feature maps across the layers of the models. The remainder of the paper is organized into the following sections: Section "Methodology" presents the methodology and dataset, Section "Results and discussion" presents the results and discussion, and Sect. “Conclusion” provides the conclusion of this paper.

## Methodology

**Fig. 1:**
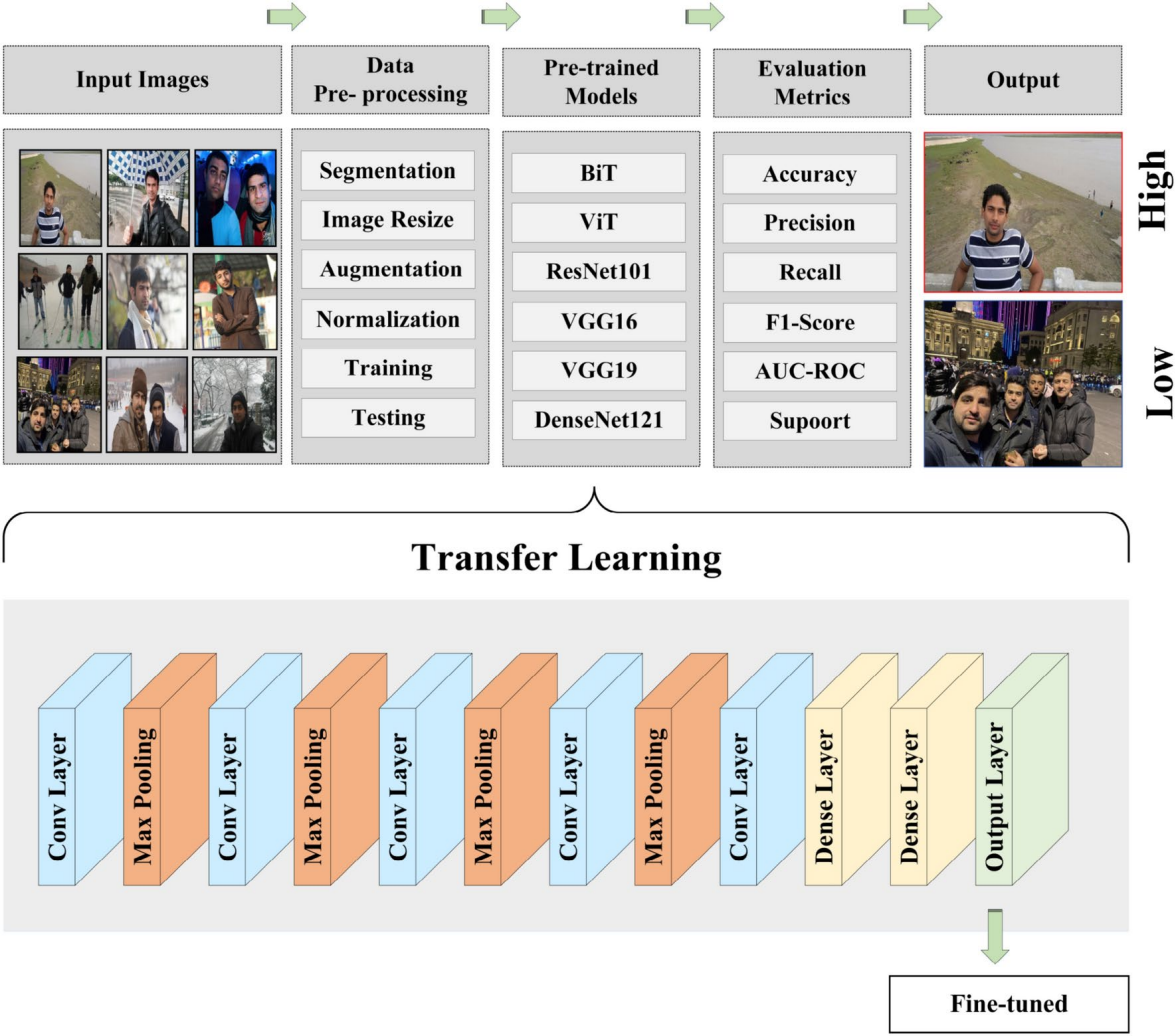
Proposed deep transfer learning framework for ambient temperature classification using human clothing images.

Deep learning is a rising area with numerous successful applications recently. It helps scientists in many domains. Deep learning has made it significantly easier to forecast and address problems in artificial intelligence (AI). CNNs have received significant attention in the field of image classification research because of their remarkable accuracy. Additionally, it effectively extracts image features and patterns, thereby improving classification accuracy. Since its inception, it has been utilized in image processing. Recent advancements in deep learning, especially in the climate and environmental domain, suggest that several deep CNN structures can be utilized. This study initiates with a comprehensive analysis of different baseline models including Big Transfer (BiT), Vision Transformer (ViT), ResNet101, VGG16, VGG19 and DenseNet121. In this study, we maintain standard convolution specifications for all baseline ImageNet models. Human-clothing images are input into the model, and images of various sizes are resized to the same dimensions. The proposed deep learning system is showed in Fig. 1.

### Dataset description

**Fig. 2:**
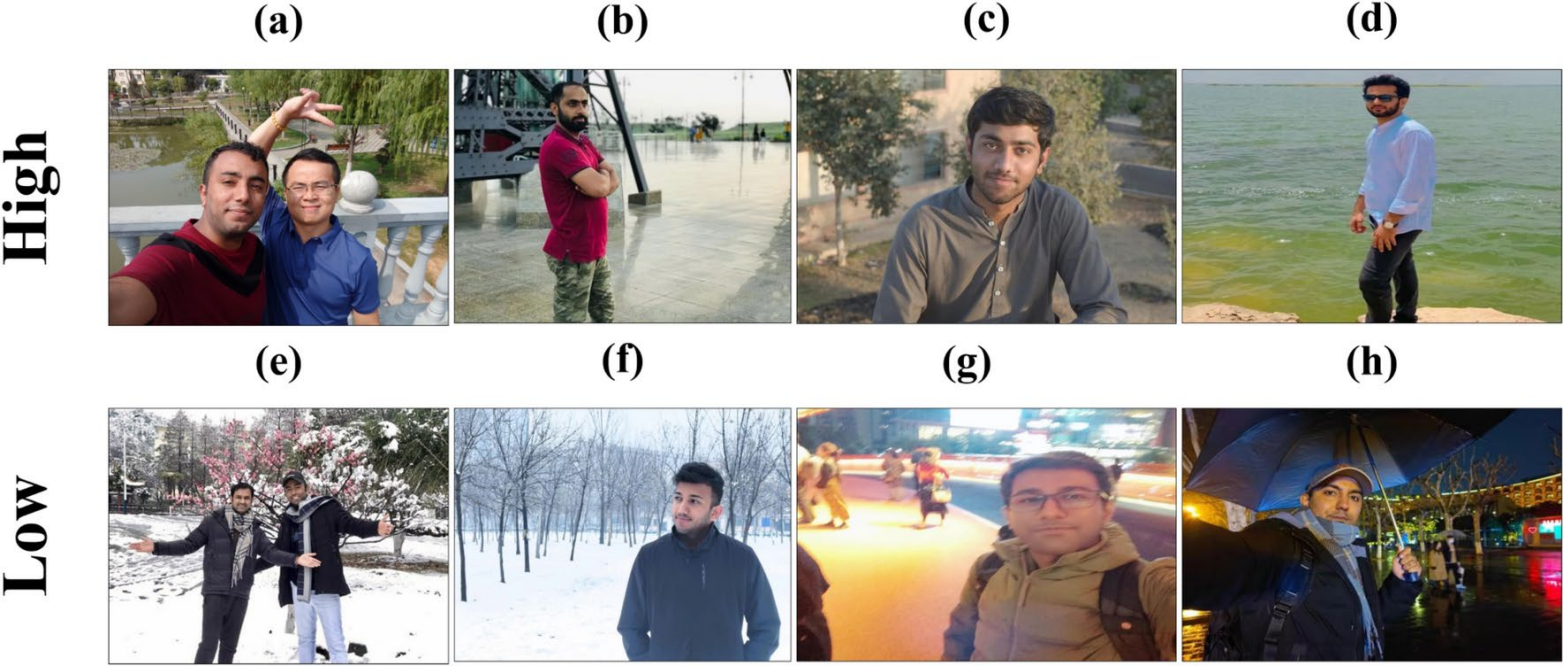
Displays eight example images from the HAID dataset, illustrating both high (**a**–**d**) and low (**e**,**h**) temperature classes.

The dataset comprises images of individuals captured across varied locations and under diverse weather conditions. Two hundred students participated in this study, including undergraduates, graduates, and postgraduates. Their historical images were collected for analysis and experimentation. The dataset consists of 10,000 images acquired from participants and categorized into two groups: Low Temperature and High Temperature. Figure 2 displays eight images of two distinct categories from the HCID dataset. The first row, consisting of images (a), (b), (c), and (d), corresponds to high temperatures, whereas the second row, comprising images (e), (f), (g), and (h), corresponds to low temperatures. Images of individuals associated with low temperatures are classified within the Low category, whereas those linked with high temperatures are categorized under the High category. The Low class comprises 5000 images, while the High class contains 5000 images. For model training, 80% of the dataset was utilized, with the remaining 20% reserved for testing. Table 1 provides this information.

**Table 1:** Human Attire Image Dataset (HCID).

Dataset	Low	High
Training	4000	4000
Testing	1000	1000
Total	5000	5000

#### Ethical considerations

Informed consent was obtained from all participants (or their legal guardians) involved in the study for the use of their clothing images for research and publication purposes. All methods were performed in accordance with relevant guidelines and regulations. Participants were thoroughly informed about the objectives of this study, the nature of the images collected, and how their data would be utilized, including potential publication in academic contexts. The study emphasized the importance of privacy, ensuring that all images were anonymized to protect the identity of individuals. No personally identifiable information was included in the dataset, and strict measures were taken to maintain confidentiality throughout the research process.

### Data preprocessing

**Fig. 3:**
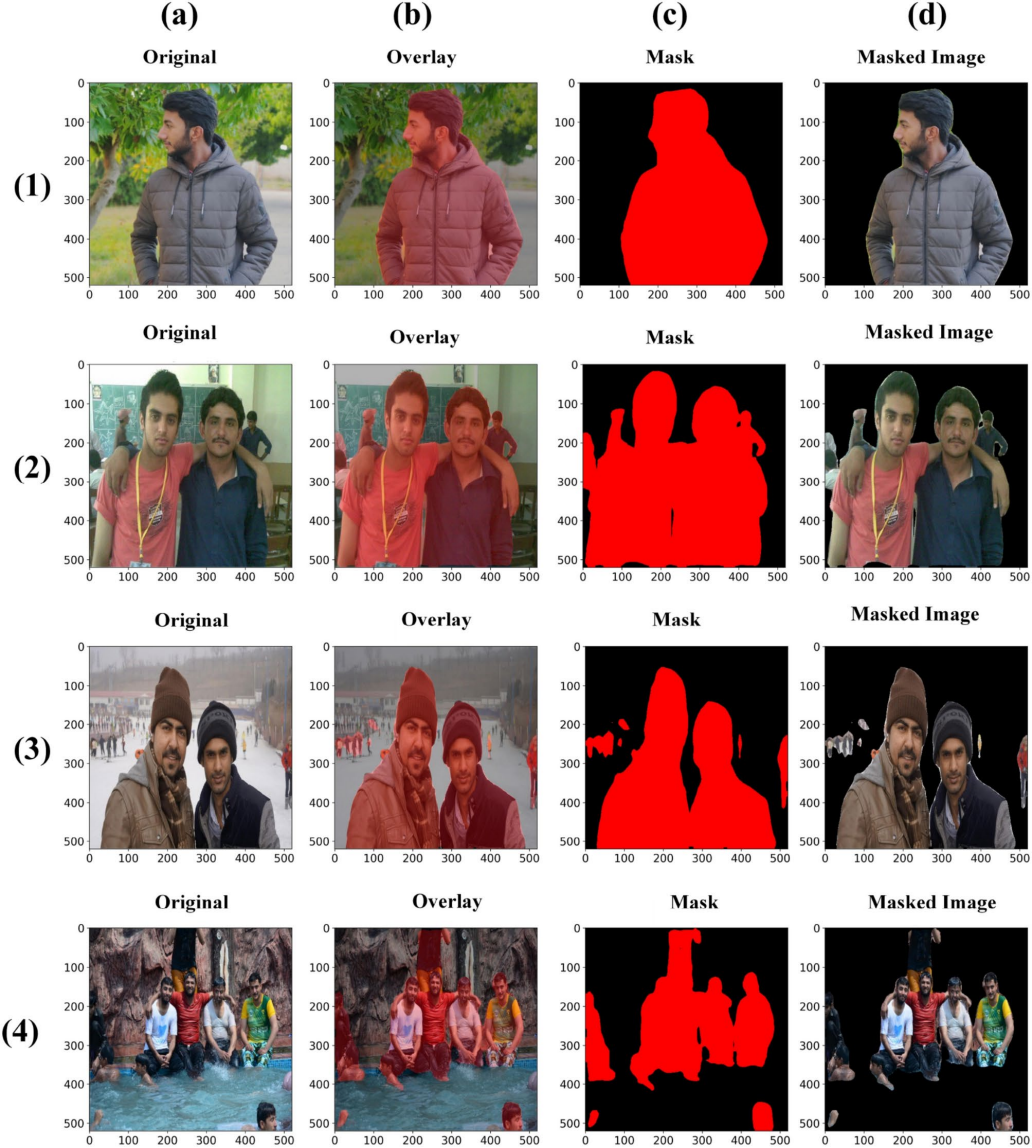
Presents examples of semantic segmentation with Person-Class using the DeepLabV3 transfer learning model.

Image preprocessing is an essential stage in working with image datasets. In this proposed work, we have utilized deep learning to create binary classifier for human clothing images to predict the presence of temperature levels. Due to difference sizes of images, we resized all images to 520 × 520 × 3 dimensions to preserve uniformity for all models. Images with human appearances also contain background elements with various objects, such as buildings, vehicles, roads, tables, chairs, and several objects, which affect the accuracy of models. DeepLabV3 Plus was then applied to the entire dataset for semantic segmentation with the “Person” (Class-15). Figure 3 illustrates examples of result images segmented with the “Person” class. Rows (1), (2), (3) and (4) represent different examples, while columns (a), (b), (c), and (d) represent different stages of processing. Column (a) shows the original input image fed into DeepLabV3 Plus, and Column (b) displays the overlay of the “Person” class generated by the model, while Column (c) depicts the application of the mask corresponding to the “Person” class. Finally, Column (d) presents the masked image, which serves as the final input for training models for temperature classification. Following segmentation, we transformed all images into pixel arrays, normalized them to a scale ranging from 0 to 1, and categorized them based on their source directory, assigning labels respectively.

Data augmentation is a technique utilized to expand datasets, facilitating enhanced feature learning on a larger scale, mitigating overfitting and boosting generalization in models. It contains several parameters in augmentation process. For example, we randomly adjusted the color saturation, hue, and brightness values for all training images. Affine transformation was performed to each image with rotation of 0 to 30. The selected shearing and zooming scale was 0.2, and we made brightness level adjustments ranging from 0.75 to 1.25.

### Pre-trained models and transfer learning

Training models from scratch requires a sizable dataset, ample resources, and significant computational power. The (HCID) dataset was utilized with transfer learning to produce an accurate and wide range of feature set to operate. Since, the most datasets containing images of people are relatively small, requiring more time to develop an efficient model. Therefore, pre-trained models were adapted and customized to create the proposed models for predicting air temperature.

#### DeepLabV3 plus

**Fig. 4:**
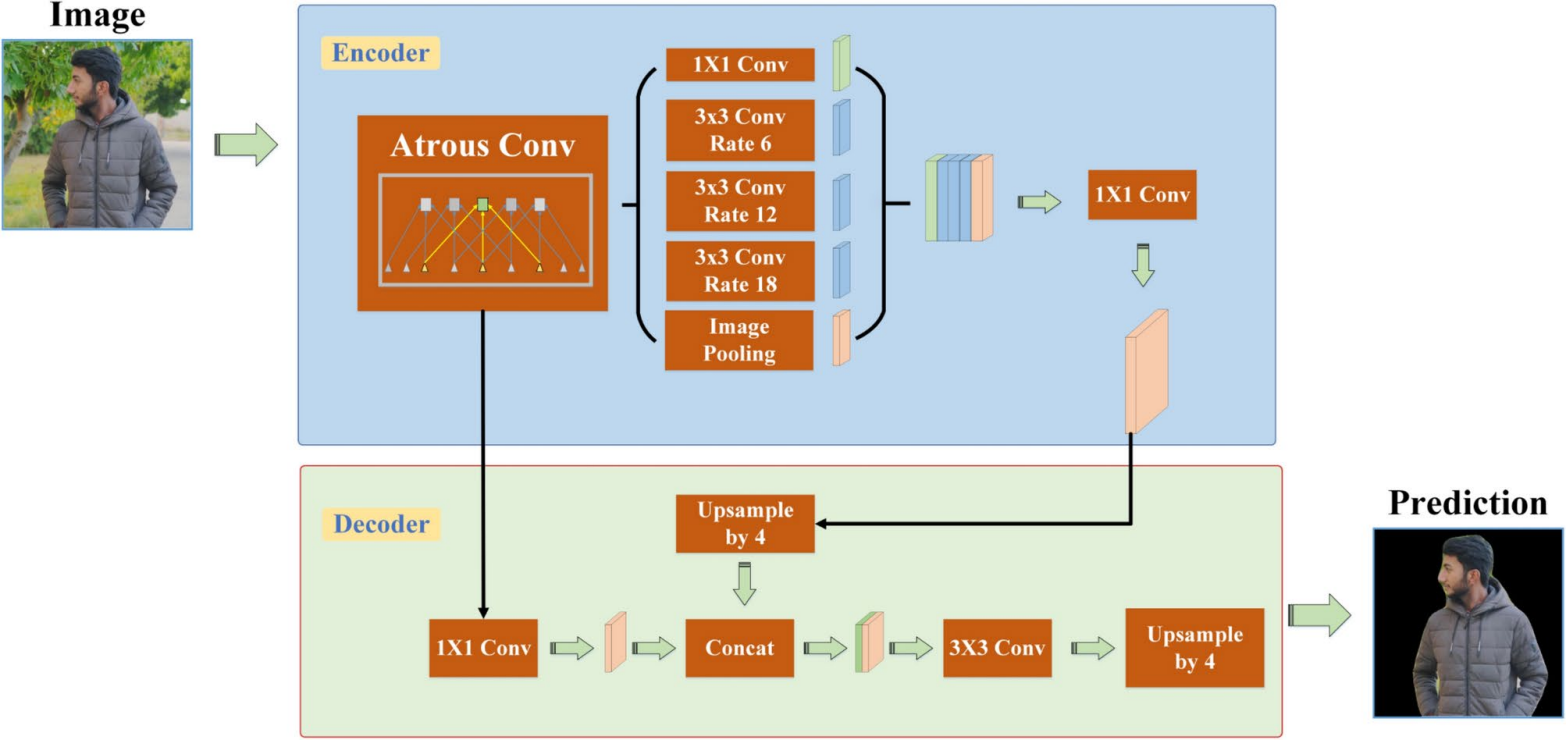
Illustrates the architecture of DeepLabV3 Plus for semantic segmentation.

The DeepLab network solves image segmentation scales with Atrous convolution method. DeepLabV1 is a technique that integers CNNs and probabilistic graphical models for Object delineation^35^. DeepLabV2 utilizes Atrous Spatial Pyramid Pooling (ASPP), employing an expanded convolutional structure with varying proportions^36^. DeepLabV3 presented enhancements to the ASPP component^37^. The DeepLabV3 Plus model combines CNNs with atrous convolution to perform image segmentation tasks. The model widens the convolutional receptive field without adding parameters, balancing speed and accuracy. Moreover, DeepLabV3 Plus employs ASPP (Atrous Spatial Pyramid Pooling) for semantic segmentation, enhancing its performance in this area. It processes and encodes input image features through convolutions with multiple expansion rates and operational fields of view, followed by a pooling operation to capture context across various scales. In DeepLabV3 Plus, a decoder is introduced which actively compresses low-level features, thereby diminishing their relative representation compared to DeepLabV3. The ASPP module processes the feature map by up-sampling to match low-level feature resolution, applying a 3 × 3 convolution, and then up-sampling again to restore spatial information, efficiently capturing refined target boundaries in images for prediction of semantic segmentation^38^. The architecture of the DeepLabV3 Plus model for semantic segmentation with Person class depicted in Fig. 4.

#### BigTransfer (BiT)

Big Transfer (BiT) was introduced by Google Research’s Brain team in 2019, a cutting-edge technique for computer vision tasks^39^. Recently, BiT is so popular due to strong transfer ability. It consists capability to perform even on tiny image datasets with less number of labels. Generally, most researchers in deep learning area accepted models with large scale architecture for better performance. Because large architecture models perform well compared to models with smaller architectures. Moreover, it achieves higher generalization due to being trained on large datasets. BiT applies the potency of scale through the upstream (pre-training) stage. Essentially, it employed large datasets and a high-performing model architecture for its training process. Consequently, this provides the better performance in downstream tasks (new tasks adjusted through fine-tuning) by leveraging well-trained weights. Moreover, BiT uses group normalization (GN) instead of batch normalization^40^, which is popular in convolutional deep learning models, in order to achieve computational efficiency on large-scale datasets. In addition, it employs weight standardization technique^41^ to avoid numerical abilities during the weight updates.

#### Resnet101

The detailed description of ResNet topologies is provided in this work^42^. In this design of the network, convolutional and pooling layers were sequentially layered in a vertical stack. This can lead to a decline in network performance as a result of vanishing gradient issues. Hence, networks with residual blocks, which incorporate shortcut connections, can significantly improve performance by mitigating training errors in deep architectures. ResNet101 is variant of ResNet structure with 101 layers.

#### Vision transformer (ViT)

The architecture of the Vision Transformer is based on the Vanilla Transformer, which has recently attracted many researchers due to its demonstration of cutting-edge performance in machine learning field^43^. The ViT architecture utilizes an encoder-decoder system with a parallel mechanism that can process sequential data without depending on any recurrent networks. The efficiency of transformer models can be attributed significantly to the self-attention mechanism. This mechanism is specially designed to detect and understand the long-distance connections among sequence elements. The ViT structure was introduced to adapt standard transformer model for image classification tasks. The primary goal is to generalize across modalities without integrating data specific architectures. The Vision Transformer uses the encoder for image classification, transforming image patches into semantic labels. Contrasting traditional CNNs that only focus on local areas with filters, it employs an attention mechanism to consider the entire image, allowing a comprehensive analysis of visual content.

#### DenseNet121

The DenseNet architecture, introduced by Gao Huang et al. in 2017^44^, utilizes dense connections, where each layer is directly connected to every other layer, enabling efficient reuse of features across the network. This interconnected structure guarantees that each layer is influenced by the parameters and feature maps of the preceding layers. Additionally, this structure enables feature reuse, where layers share features learned by earlier layers, reducing redundancy and improving efficiency. This helps the model capture complex patterns with fewer parameters, leading to better performance. There are several variants of the DenseNet architecture frequently used by researchers in image classification tasks, one of them is DenseNet121, which consists of 121 layers and is utilized in this study.

#### VGG16 & VGG19

The VGGNet architecture, introduced in 2014 by the University of Oxford’s Visual Geometry Group^45^, is a highly popular deep convolutional model. It achieved both first prize and second place in image classification at the ILSVRC 2014. The initial idea was to design this model to perform accurate image processing tasks with various kernels. Recently, researchers have widely utilized the VGG architecture to extract deep image patterns and features across different domains for further processing in computer vision tasks. VGG16 consists of 16 layers, whereas VGG19 comprises 19 layers within its architecture.

### The proposed models with customization

**Fig. 5:**
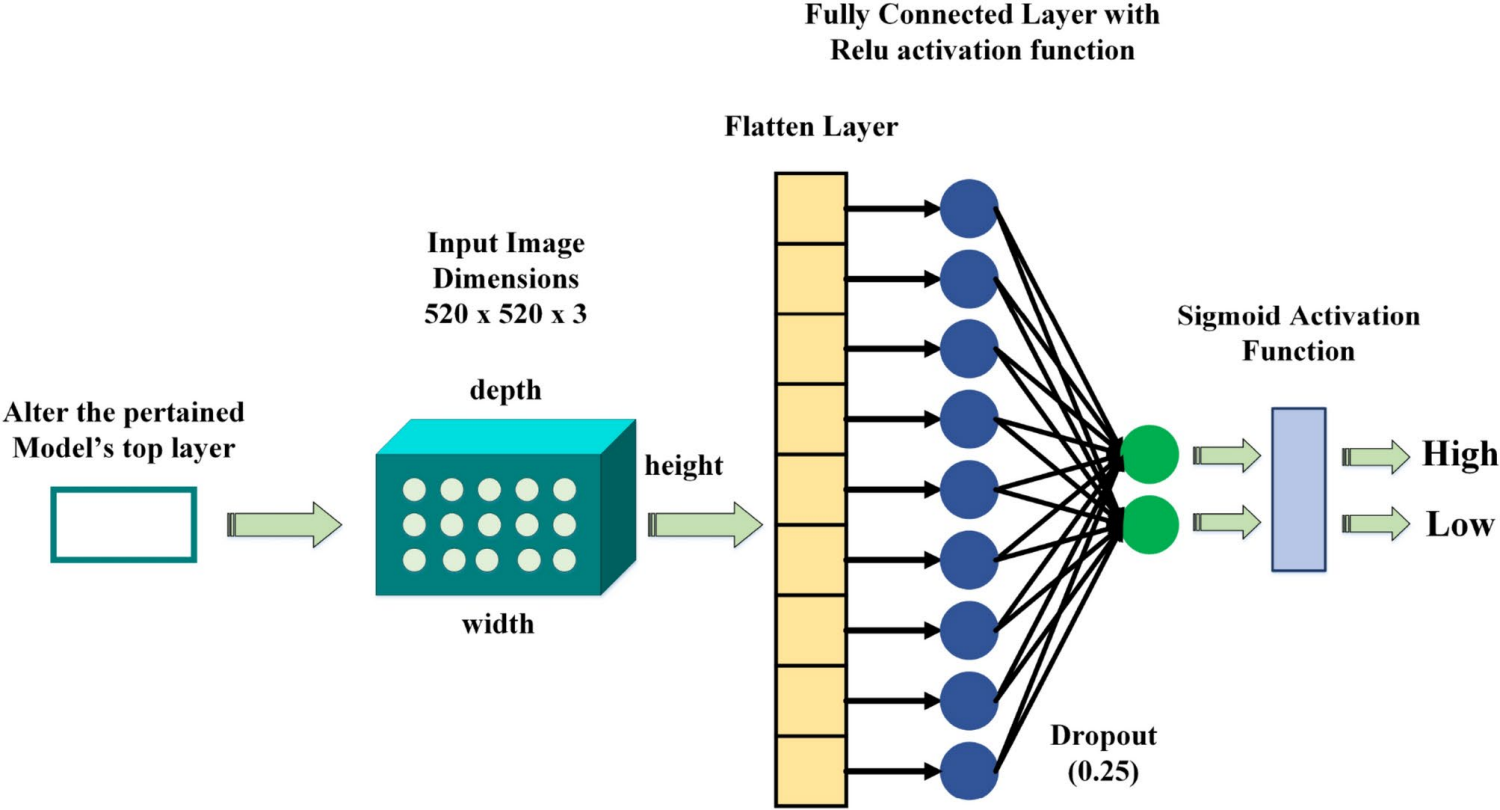
Customized model structure for a proposed deep transfer learning framework designed for the classification of two classes: High and Low temperatures.

Following the preprocessing step, the dataset was partitioned into two subsets: 80% allocated for training the models, while the remaining 20% was reserved for testing purposes. Neural networks can be applied on different types of data tasks, CNNs are one of them, specially designed for image classification. Most research employs CNNs for the analysis of visual imagery and often plays a vital role in image classification. The Python package TensorFlow, a deep learning framework, was used to train CNNs using the Keras library. Due to the large size of the ImageNet dataset, that covers approximately 1.2 million images, it is often used to build numerous architectures for generating general models. To achieve generalization beyond ImageNet, transfer learning techniques are applied in this study. This can only be done with pre-trained models. Fine-tuning was also applied to pre-trained models to capture changes. We constructed our models utilizing a variety of architectures, such as BigTransfer (BiT), ResNet101, Vision Transformer (ViT), DenseNet121, VGG16, and VGG19. For instance, the VGG19 convolutional neural network (CNN) incorporates a 2D Max Pooling layer yielding an output shape of (7, 7, 512) with zero parameters, and it can be imported via the Keras applications class. Transfer learning was employed to address a two-class classification problem distinguishing between high and low temperatures. This involved modifying the output layer to function as a binary classifier. The system retrieved the outputs from the preceding layer and fed them into a flatten layer with a default shape of (7, 7, 512), following the max pooling output layer, resulting in a flattened matrix with dimensions of 7 × 7 × 512, totaling 25,088. We generally perform this flattening of rectangular shapes at the end of CNN, as they cannot directly input into the network. We introduced a dropout layer as a regularization technique to mitigate overfitting and improve generalization error. As our goal is to create a binary classifier, so we set the dropout value to 0.25 for the layer, a common choice. This principle is based on the observation that expansive neural networks often face an overfitting problem when trained on small datasets, leading to a reduction in validation accuracy. The customization of the proposed transfer learning model is illustrated in Fig. 5.

The output layer was introduced with two dense layers and a sigmoid function for predicting high and low temperature levels. The sigmoid function is used at the output layer to forecast a multinomial probability distribution for neural network models. Because we have a binary classifier that requires two dense layers, the Adam optimizer and the categorical cross-entropy loss function were utilized to build the model. The CNN employs cross-entropy loss during training to compute class probabilities for each image, facilitating probabilistic image classification.

### Performance metrics

In this study, we evaluate our proposed models through classification, employing metrics such as Accuracy, Sensitivity, Specificity, F1-Score, Precision, Recall, and ROC-AUC. These evaluation metrics are valuable for assessing the temperature classification system based on images. These metrics can be mathematically expressed as follows:

$$Accuracy=\frac{{T}_{Pos}+ {T}_{Neg}}{{T}_{Pos}+{F}_{Neg}+{T}_{Neg}+{F}_{Pos}}\times 100$$

$$Sensitivity=\frac{{T}_{Pos}}{{T}_{Pos}+{F}_{Neg}}\times 100$$

$$Specificity=\frac{{T}_{Neg}}{{T}_{Neg}+{F}_{Pos}}\times 100$$

$$F1-Score=\frac{2\times {T}_{Pos}}{2\times {T}_{Pos}+{F}_{Neg}+{F}_{Pos}}$$

$$Precision=\frac{{T}_{Pos}}{{T}_{Pos}+{F}_{Pos}}$$

$$Recall=\frac{{T}_{Pos}}{{T}_{Pos}+{F}_{Neg}}$$

## Results and discussion

### Model training

The proposed models underwent training and testing procedures utilizing the PyTorch deep learning framework. The computer configuration for the models employed dual CPUs with Intel Core i5 processors, equipped with 32 GB of RAM, and a 1024 GB hard disk. In this work, we selected the hyper-parameters based on prior research and carried out several experiments. The Adam optimizer integrates the advantages of the AdaGrad and RMSProp algorithms while consuming less memory compared to other optimization approaches. As a result, The Adam optimizer enhances the optimization of the cross-entropy loss. To ensure optimal training of the proposed models, the training process involves selecting an initial learning rate of 0.0001, a batch size of 64, and a validation frequency of 10, spanning 200 epochs. Table 2 illustrates the model parameters, and all six models are executed with an identical experimental setup.

**Table 2:** Presents the parameters employed across all models in this study.

Parameters	Big Transfer (BiT)	Vision Transformer (ViT)	Resnet101	VGG16	VGG19	DenseNet121
Image size	520 × 520x3	520 × 520x3	520 × 520x3	520 × 520x3	520 × 520x3	520 × 520x3
Batch size	64	64	64	64	64	64
Optimizer	Adam	Adam	Adam	Adam	Adam	Adam
Learning rate	0.0001	0.0001	0.0001	0.0001	0.0001	0.0001
Epochs	200	200	200	200	200	200
Dense Layer Activation Function	Relu	Relu	Relu	Relu	Relu	Relu
Dropout	0.5	0.5	0.5	0.5	0.5	0.5
Output Layer	Sigmoid	Sigmoid	Sigmoid	Sigmoid	Sigmoid	Sigmoid
Activation/ Loss Function	Cross-Entropy	Cross-Entropy	Cross-Entropy	Cross-Entropy	Cross-Entropy	Cross-Entropy

### Experiment outcomes and analysis

#### The accuracy and loss graphs

**Fig. 6:**
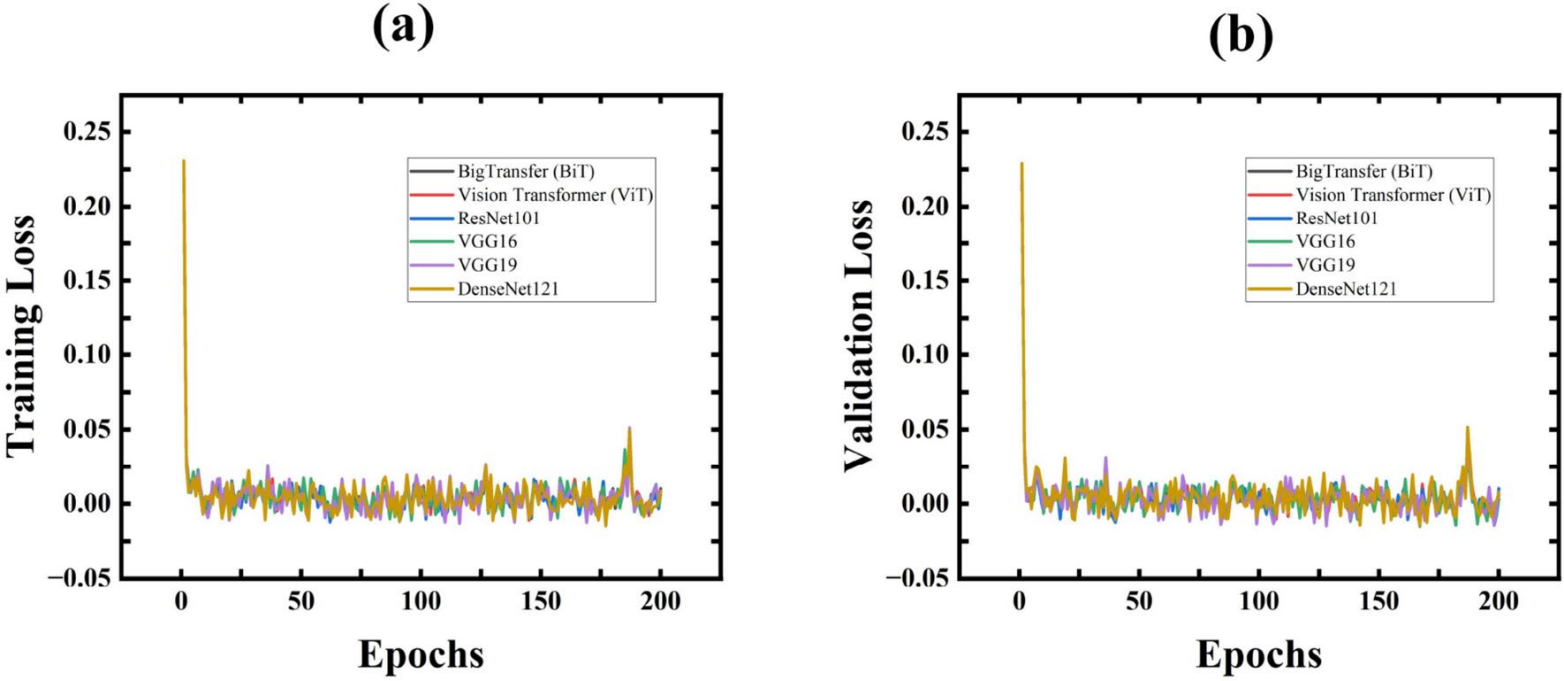
Training Loss and Validation Loss for All Proposed models.

**Fig. 7:**
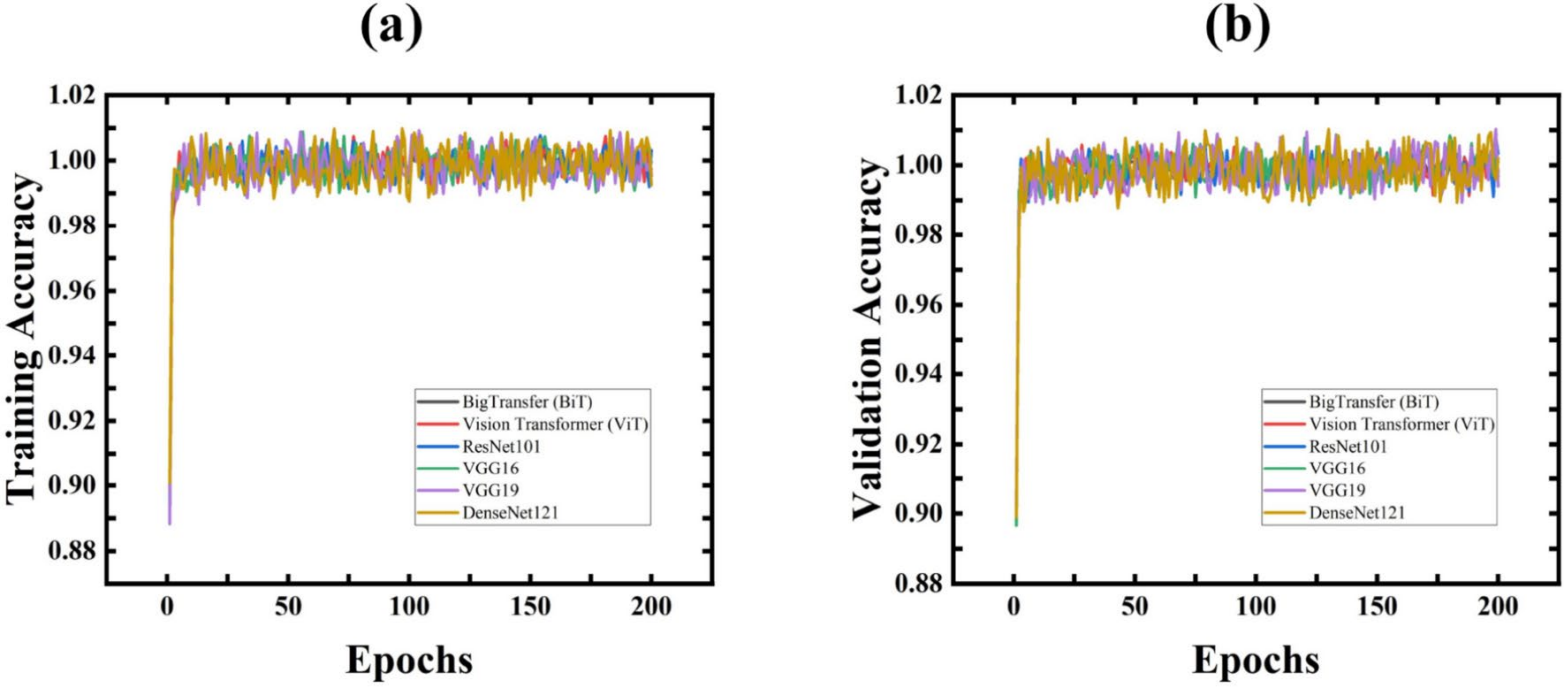
Training Accuracy and Validation Accuracy for All Proposed models.

The accuracy and loss graphs serve as crucial tools for visualizing model performance during the training phase. Figure 6a,b illustrates the training and validation losses, while Fig. 7a,b depicts the training and validation accuracies. Observing Fig. 6a,b demonstrates a rapid decrease in both training and validation losses, indicating optimal classification performance. Additionally, in Fig. 7a,b, it becomes evident that the models achieved comparable accuracies after a few epochs.

#### The ROC curve and AUC

**Fig. 8:**
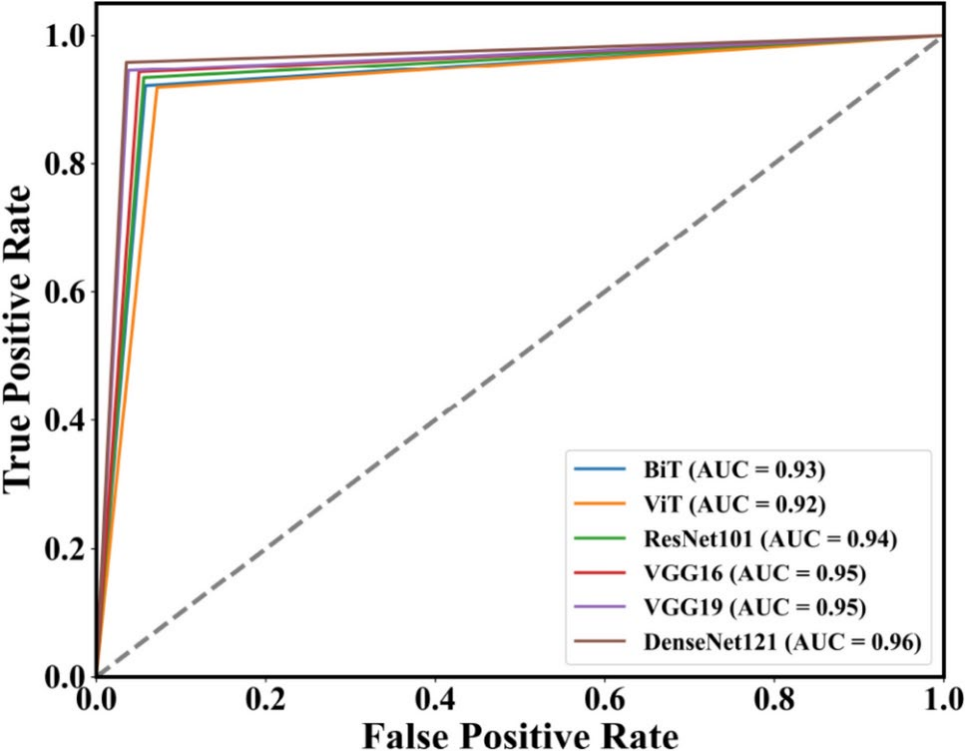
ROC-AUC Curve for BiT, ViT, ResNet101, VGG16, VGG19 and DenseNet121.

The ROC curve serves as a pivotal tool for evaluating the performance of classification models^46^. Within the ROC curve diagram, each point delineates a pair of values: The False Positive Rate (FPR) on the x-axis and the True Positive Rate (TPR) on the y-axis. The Area Under the Curve (AUC) quantifies the entirety of the area encompassed by the curve, providing a comprehensive measure of model performance. The performance improvement of a classification model is indicated by the proximity of its ROC curve to the upper-left corner, and a greater area under the curve corresponds to superior classification outcomes. It can be observed from Fig. 8 that DenseNet121 achieved the highest AUC of 0.96, demonstrating the best ROC curve among all models.

#### Confusion Matrix (CM)

**Fig. 9:**
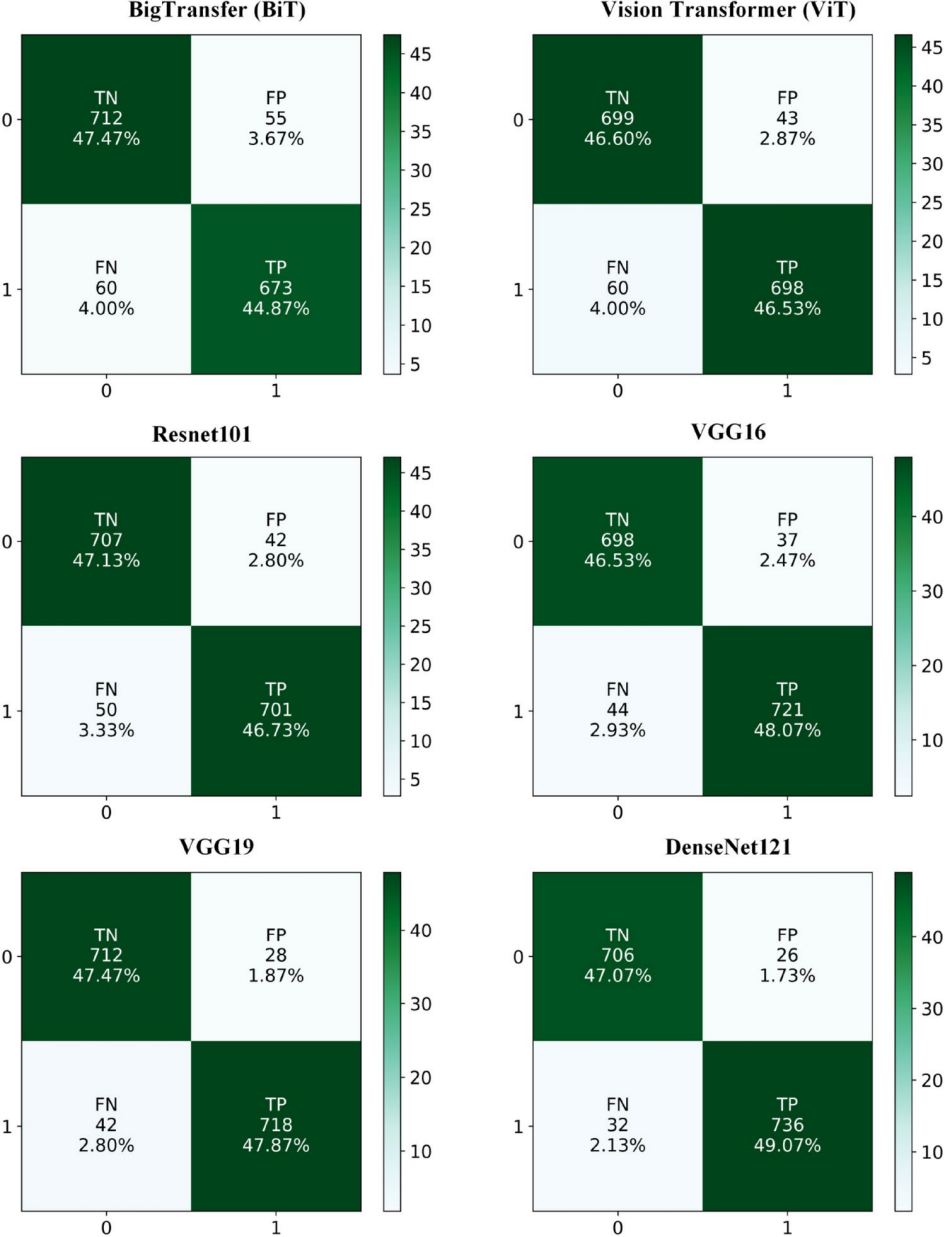
Confusion Matrix (CM) for proposed deep learning models.

The confusion matrix is specifically employed to evaluate classification problems^47^. Figure 9 shows the confusion matrix of predicted results generated by proposed models including BigTransfer(BiT), Vision Transformer(ViT), ResNet101, VGG16, VGG19, and DenseNet121. The horizontal axis of the confusion matrix represents the predicted class labels, typically denoted as 0 and 1, corresponding to the test set. Meanwhile, the vertical axis signifies the actual class labels of the test samples, also represented as 0 and 1 respectively. Where 0 signifies the High class and 1 indicates the Low class category. In the confusion matrix, the predicted values of the model correspond with the actual values of the test samples along the diagonal. In Fig. 9, the diagonal values denote the count of accurately classified images corresponding to different temperature levels, whereas the values outside the diagonal indicate the count of misclassified images across temperature levels. For example, out of the total test samples, 712 (representing 47.47%) instances associated with class 0 (High Temperature) were accurately classified by the BigTransfer (BiT) model. However, in the same dataset, there were 55 samples (equating to 3.67%) originally categorized as class 0 (High Temperature) that were misclassified as class 1 (Low Temperature) by the BiT. It can be seen that, among the six models, DenseNet121 achieved the highest correct classification rate, with a (TN) value of 706 (47.07%) and a (TP) value of 736 (49.07%). It also had a relatively low misclassification rate, with a (FP) value of 26 (1.73%) and a (FN) value of 32 (2.13%). On the other hand, BigTransfer (BiT) demonstrated a high misclassification rate, with a (FP) value of 55 (3.67%) and a (FN) value of 60 (4%). It achieved a (TN) value of 712 (47.47%) and a (TP) value of 673 (44.87%). The remaining models, including Vision Transformer (ViT), ResNet101, VGG16, and VGG19, showed moderate misclassification and correct classification rates. Overall, these findings indicate that DenseNet121 is the most effective model for classification of air temperature levels from mobile images, while BigTransfer is the least accurate and the remaining models, namely Vision Transfer (ViT), ResNet101, VGG16, and VGG19, showed an average level of performance. The performance indicators for the proposed models for each class (0 and 1) are shown in Tables 3 and 4.

**Table 3:** Comparison results of High Class (class-0).

Metrics	BiT	ViT	ResNet101	VGG16	VGG19	DenseNet121
Precision	92.22	92.09	93.39	94.07	94.42	98.66
Recall	92.82	94.20	94.39	94.96	96.21	98.44
F1-score	92.52	93.13	93.89	94.51	95.31	99.05
Support	767	742	749	735	740	749
ROC/AUC	93.67	96.85	96.13	95.39	94.65	98.85

**Table 4:** Comparison results of Low Class (class-1).

Metrics	BiT	ViT	ResNet101	VGG16	VGG19	DenseNet121
Precision	92.44	94.19	94.347	95.11	96.24	98.58
Recall	91.81	92.08	93.34	94.24	94.47	98.83
F1-score	92.12	93.12	93.84	94.68	95.35	99.20
Support	733	758	751	765	760	751
ROC/AUC	92.32	93.14	93.86	94.60	95.34	98.14

#### Comparative analysis of model performance for air temperature classification from clothing images

The analysis of the performance metrics provides valuable insights into the effectiveness of various deep learning models in classifying air temperature based on human clothing images. The models compared include BiT, ViT, ResNet101, VGG16, VGG19, and DenseNet121. Among these, DenseNet121 consistently outperformed the others across all key performance metrics, such as accuracy, sensitivity, specificity, F1-score, precision, recall, and AUC. These results highlight the advantages of DenseNet121 architecture, which are instrumental in achieving its superior performance.

DenseNet121 achieved the highest accuracy at 98.13%, along with the highest sensitivity (97.83%) and specificity (98.44%). These metrics indicate that DenseNet121 not only correctly identifies the majority of true positive instances but also excels in minimizing false positive cases. The F1-score of 97.05% and AUC of 98.14% confirm a robust balance between precision and recall, underscoring robustness across different metrics. The strength of DenseNet121 lies in its densely connected convolutional layers, which promote efficient feature reuse and improve gradient flow across the network. This design minimizes the number of parameters while preserving high representational capacity, making the model more compact and efficient. The densely connected layers enable DenseNet121 to learn more robust features, a critical aspect in handling the complexity of visual data such as clothing images used for temperature classification. Consequently, the ability of model to maintain high precision and recall demonstrates its effectiveness in reducing both false positives and false negatives, key factors in achieving accurate classification results^44^. In contrast, ResNet101, which achieved an accuracy of 93.86%, struggled to match the performance of DenseNet121. While its sensitivity (93.34%) and specificity (94.39%) were relatively high, these values suggest some degree of overfitting, likely due to the deep architecture of network. While residual connections of ResNet101 are beneficial in mitigating the vanishing gradient problem, its deeper architecture of 101 layers can lead to overfitting, especially with datasets that are not sufficiently large or diverse. This overfitting issue may result in the model performing well on the training data but struggling to generalize effectively to new, unseen data, thus negatively affecting its precision and recall^48^. Similarly, ViT, with an accuracy of 93.13% and a sensitivity of 92.08%, fell short of DenseNet121, further illustrating the limitations of self-attention-based models in scenarios with more complex visual patterns like clothing images. ViT treats image patches as sequences similar to how tokens are used in natural language processing. Although ViT has shown promise in some domains, its effectiveness is often reliant on large datasets for training. In scenarios like this one, where the dataset size is relatively limited, CNN models like DenseNet121 tend to outperform ViT due to their ability to learn hierarchical and spatial features more effectively. The self-attention mechanism of ViT, while powerful for capturing long-range dependencies, may struggle to capture local spatial details critical for accurate image classification, such as distinguishing different temperature classes based on subtle variations in clothing^49^.

Both VGG16 and VGG19 performed well, particularly in terms of recall and F1-score, which can be attributed to their deep, sequential convolutional layers that facilitate extensive feature extraction. VGG19 achieved a recall of 96.21%, narrowly trailing DenseNet121, while VGG16 recorded a recall of 94.96%. However, these models also exhibited a tendency toward overfitting due to their high parameter count and depth, leading to a slight reduction in their overall accuracy compared to DenseNet121^50^. While, BiT demonstrating respectable performance with an accuracy of 92.33%, lagged behind the other models, particularly in precision (92.22%) and F1-score (92.52%). This may be attributed to reliance of BiT on transfer learning and the specific characteristics of the training dataset, which may not align optimally with the pre-trained model knowledge^51^.

#### Impact of semantic segmentation on air temperature classification performance

**Fig. 10:**
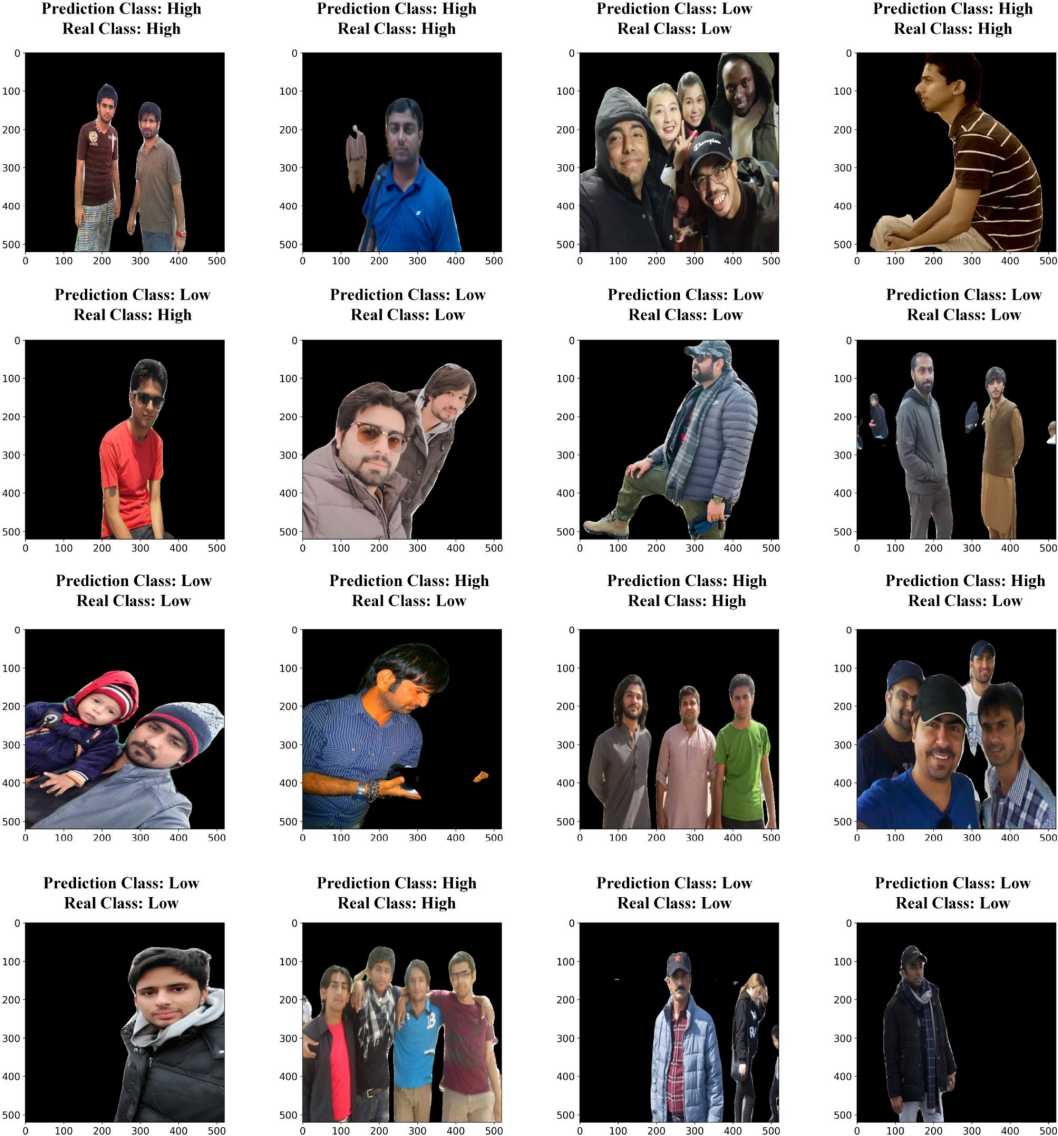
Comparison of Actual vs. Predicted Classification Results with Image Examples.

The implementation of semantic segmentation, particularly through the DeepLabV3 Plus architecture, has demonstrated a significant impact on the classification performance of models for air temperature classification using human clothing images. The results presented in Table 5 illustrate that all models trained on the segmented dataset achieved higher performance metrics compared to those trained on the non-segmented dataset (Table 6), with performance dropping by almost 50%. For instance, the accuracy of the DenseNet121 model on the segmented dataset reached 98.13%, while the same model only achieved 56.62% accuracy on the non-segmented dataset. Figure 10 shows the actual and predicted results generated by DenseNet121 on randomly sampled images from the segmented dataset, further illustrating the impact of segmentation. This stark contrast underscores the critical role that semantic segmentation plays in enhancing the ability of models to discern relevant features from the images, which is essential for accurate classification tasks^52,53^. The observed drop in performance metrics such as accuracy, sensitivity, specificity, F1-Score, precision, recall, and AUC in the absence of segmentation can be attributed to inability of model to effectively isolate and identify the relevant features of clothing that correlate with varying air temperatures. Without semantic segmentation, the models are presented with cluttered images where the background and irrelevant details can obscure the critical features of clothing, leading to misclassification and reduced sensitivity to temperature related cues^54^. The segmentation process allows the models to focus on the pertinent regions of interest, thereby improving their ability to generalize and classify accurately. This is particularly important in the context of clothing images, where variations in color, texture, and layering can significantly influence temperature classification outcomes^55^.

**Table 5:** Overall performance metrics achieved for proposed models dataset.

Parameters	BiT	ViT	ResNet101	VGG16	VGG19	DenseNet121
Accuracy (%)	92.33	93.13	93.86	94.65	95.33	98.13
Sensitivity (%)	91.81	92.08	93.34	94.24	94.47	97.83
Specificity (%)	92.82	94.20	94.39	94.96	96.21	98.44
F1-Score	92.52	93.13	93.89	94.51	95.31	97.05
Precision (%)	92.22	92.09	93.39	94.07	94.42	97.66
Recall	92.82	94.20	94.39	94.96	96.21	98.44
AUC (%)	92.32	93.14	93.86	94.60	95.34	98.14

**Table 6:** Overall performance metrics achieved for proposed models on non-segmented dataset.

Parameters	BiT	ViT	ResNet101	VGG16	VGG19	DenseNet121
Accuracy (%)	54.33	51.06	52.21	53.93	54.31	56.62
Sensitivity (%)	53.43	50.57	52.93	53.63	52.52	57.82
Specificity (%)	52.11	50.84	53.56	52.38	53.73	56.26
F1-Score	53.47	51.82	52.66	51.74	52.81	57.37
Precision (%)	56.32	50.99	51.78	52.53	53.66	57.86
Recall	52.11	51.63	52.37	53.92	52.78	56.11
AUC (%)	53.81	51.55	52.73	52.33	53.24	57.48

Moreover, the DeepLabV3 Plus architecture employs atrous convolution, which enables the model to capture multi scale contextual information while maintaining spatial resolution. This capability is crucial for semantic segmentation tasks, as it allows the model to learn from a broader context of the image, enhancing its understanding of the relationships between different clothing items and their corresponding temperature classifications^52,53^. The ability to maintain high spatial resolution while capturing contextual information is a significant advantage that contributes to the superior performance metrics observed in the segmented dataset. In contrast, the non-segmented dataset lacks this refined feature extraction, resulting in a loss of critical information that is necessary for accurate classification^54^. The necessity of employing semantic segmentation, particularly through advanced architectures like DeepLabV3 Plus, is evident in the substantial improvements in classification performance for air temperature classification using human clothing images. The marked differences in performance metrics between the segmented and non-segmented datasets highlight the importance of isolating relevant features from complex images to enhance model accuracy and reliability. This approach not only improves classification outcomes but also provides a more robust framework for understanding the intricate relationships between clothing attributes and temperature classifications, ultimately leading to more effective applications in real world scenarios^52,53^.

**Fig. 11:**
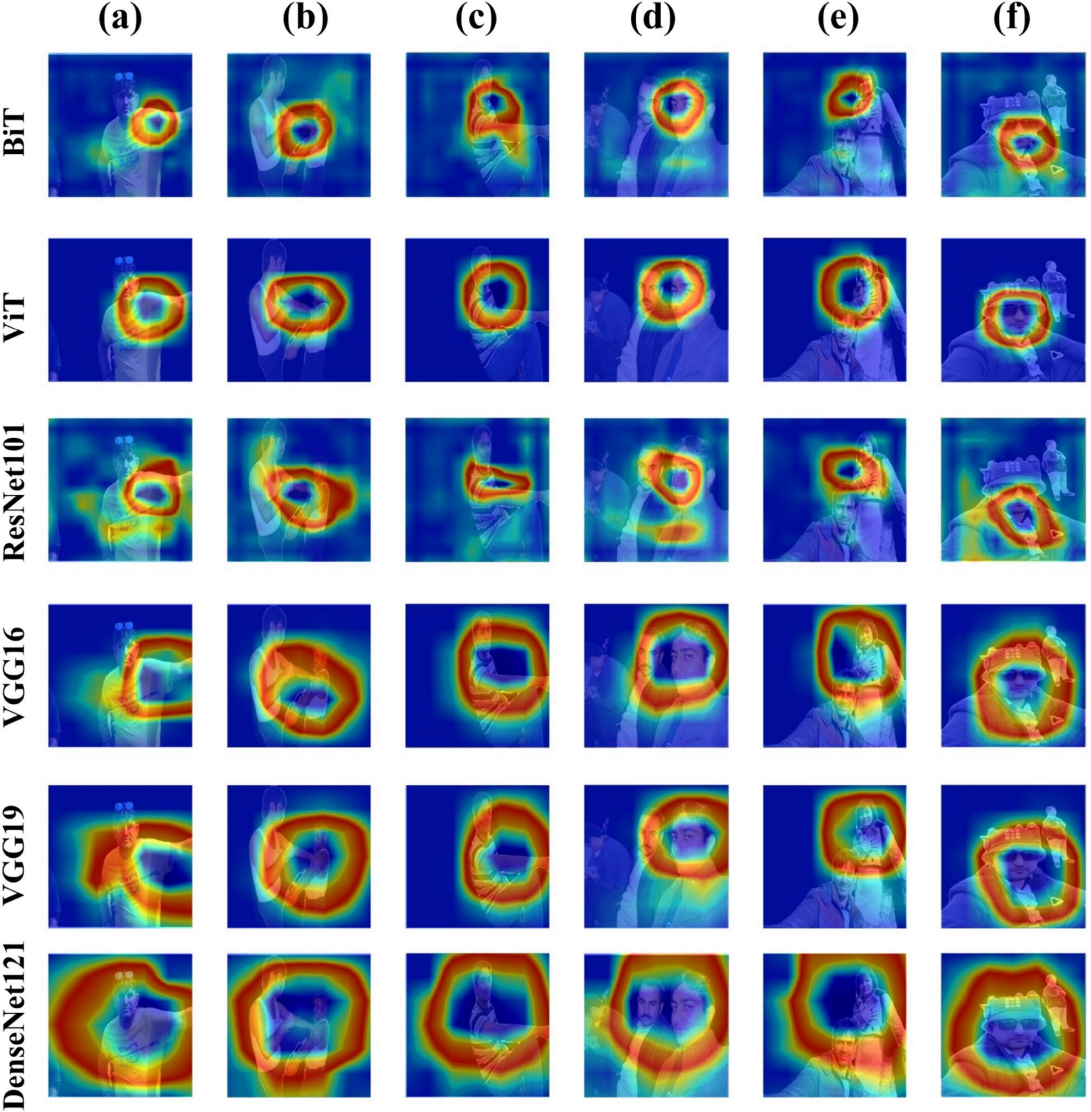
Gradient-weighted Class Activation Mapping (Grad-CAM) for all proposed models.

To further validate the deep transfer learning methods employed in this study, we applied Gradient-weighted Class Activation Mapping (Grad-CAM) technique^56^ to visualize the essential pixels and area considered by the model in computation process for classification. The images are randomly selected from test set to perform this approach for all models. Figure 11 shows the resulting images with feature maps, where columns represent images (a) through (f), and rows represent different models. The first row presents the results generated by the BiT model, while the second row shows the results produced by ViT. In (a), (c), and (f) samples, BiT predictions are correct, while (b), (d), and (e) are incorrect predictions. ViT produced four correct predictions: (a), (b), (d), and (f), and two incorrect predictions: (c) and (e). ResNet produced four correct predictions: (a), (b), (d), and (e), and two wrong predictions: (c) and (f). VGG16 and VGG19 both produced five correct predictions. For VGG16, it corrected (a), (c), (d), (e), and (f), while (b) is incorrect. For VGG19, (a), (b), (c), (e), and (f) are correct, and (d) is incorrect. But DenseNet121 made accurate predictions for all six samples. Results show that, compared to other models, DenseNet121 exhibits a preference for broader scopes, integrating a greater amount of contextual feature data due to the depth of convolutional layers in architecture. Following DenseNet121, VGG16 and VGG19 took second place, while BiT, ViT, and ResNet101 demonstrated moderate performance.

## Conclusion

Human attire images can represent the temperature levels induced by various weather phenomena. Deep learning is a powerful technique for detecting temperature levels using images. This paper presents a deep transfer learning framework for detecting temperature levels from mobile images. The framework consists of six different powerful deep learning models applied on HAID dataset for the classification task, including BigTransfer (BiT), Vision Transformer (ViT), ResNet101, VGG16, VGG19, and DenseNet121. DeepLabV3 Plus was also applied for semantic segmentation job. Furthermore, the Grad-CAM technique was performed to assess the classification performance and visualize the feature maps. The classification results, covering all metrics, demonstrate that DenseNet1121 outperformed other models in the classification of human attire images into high and low temperature classes. The developed system can be applied in weather phenomena recognition and environmental monitoring.

## Supplementary Information

Supplementary Information.

## Data Availability

All data generated or analyzed during this study are included in this published article and its supplementary information files.
