# Supplementary figures and images for: A deep transfer learning based convolution neural network framework for air temperature classification using human clothing images (part 2 of 2)
Source: Sci Rep. 2024 Dec 30;14:31658. doi: 10.1038/s41598-024-80657-y (PMC11685903; doi:10.1038/s41598-024-80657-y)

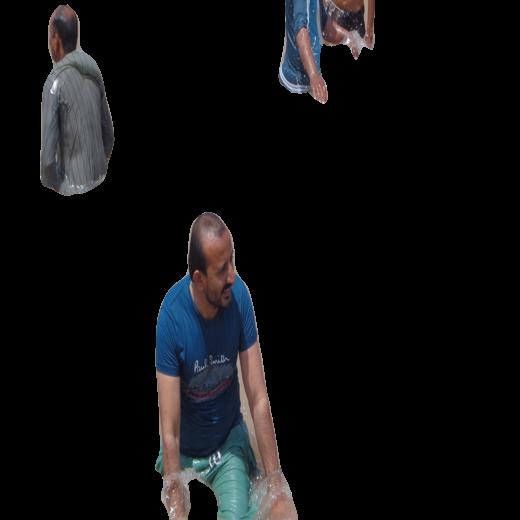

Supplement: Supplementary file 1 — Supplementary Information. [file 41598_2024_80657_MOESM1_ESM.zip › Dataset/High temperature human images/masked_10348924_725951040781284_4523637239526119392_o.jpg]

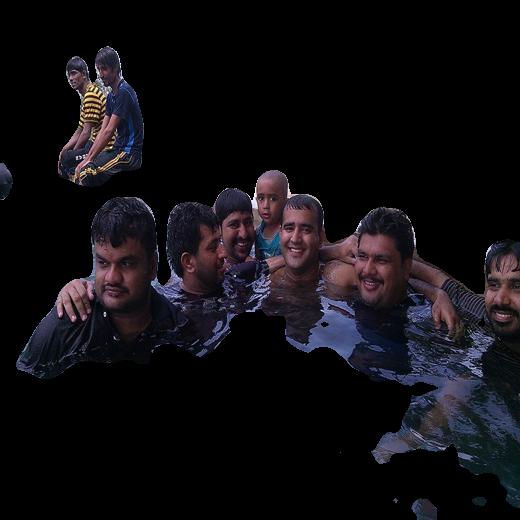

Supplement: Supplementary file 1 — Supplementary Information. [file 41598_2024_80657_MOESM1_ESM.zip › Dataset/High temperature human images/masked_10349023_752157401489627_7881384426039365339_n.jpg]

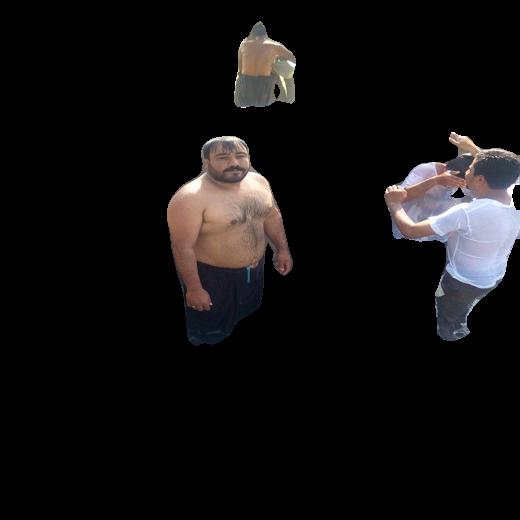

Supplement: Supplementary file 1 — Supplementary Information. [file 41598_2024_80657_MOESM1_ESM.zip › Dataset/High temperature human images/masked_10349949_690081314362727_6195987759243248970_n.jpg]

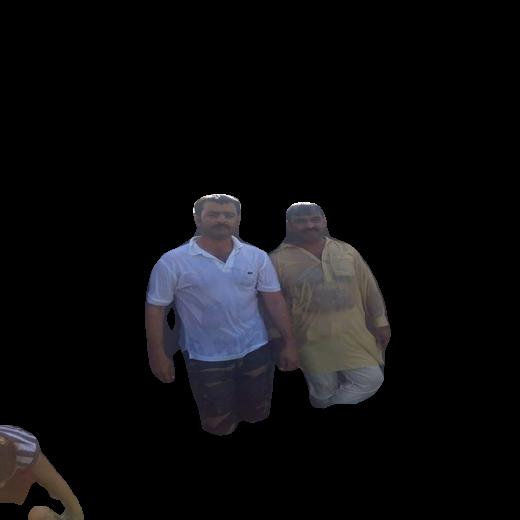

Supplement: Supplementary file 1 — Supplementary Information. [file 41598_2024_80657_MOESM1_ESM.zip › Dataset/High temperature human images/masked_10351813_690081207696071_1191031693110919920_n.jpg]

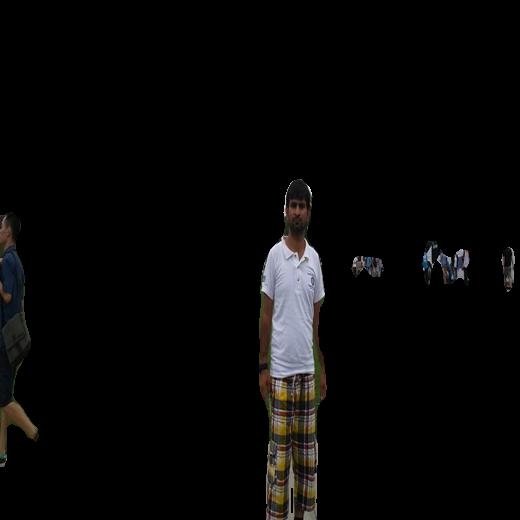

Supplement: Supplementary file 1 — Supplementary Information. [file 41598_2024_80657_MOESM1_ESM.zip › Dataset/High temperature human images/masked_10351880_789750204410833_2278924583273959658_n.jpg]

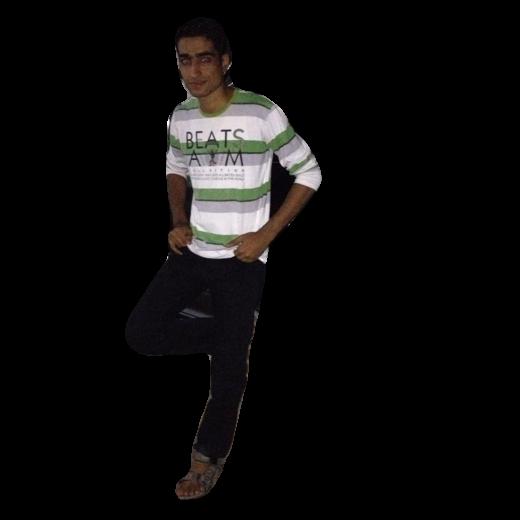

Supplement: Supplementary file 1 — Supplementary Information. [file 41598_2024_80657_MOESM1_ESM.zip › Dataset/High temperature human images/masked_10352033_608970522551862_6122010847783364106_n.jpg]

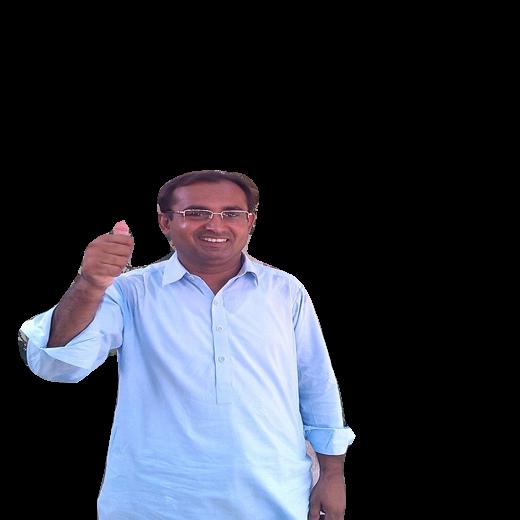

Supplement: Supplementary file 1 — Supplementary Information. [file 41598_2024_80657_MOESM1_ESM.zip › Dataset/High temperature human images/masked_10352324_752559181449449_8003031797592413066_n.jpg]

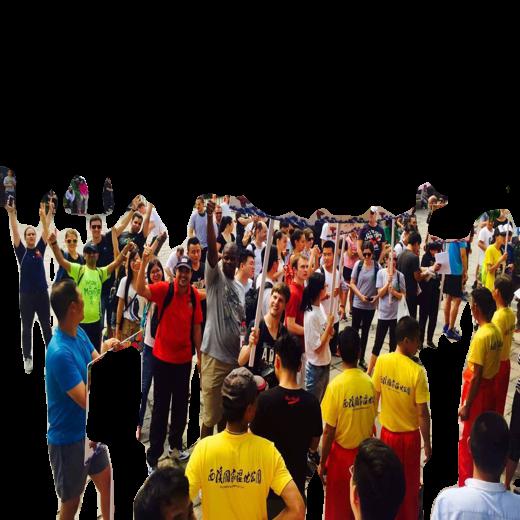

Supplement: Supplementary file 1 — Supplementary Information. [file 41598_2024_80657_MOESM1_ESM.zip › Dataset/High temperature human images/masked_10353255_943731122346073_5459196075792958591_o.jpg]

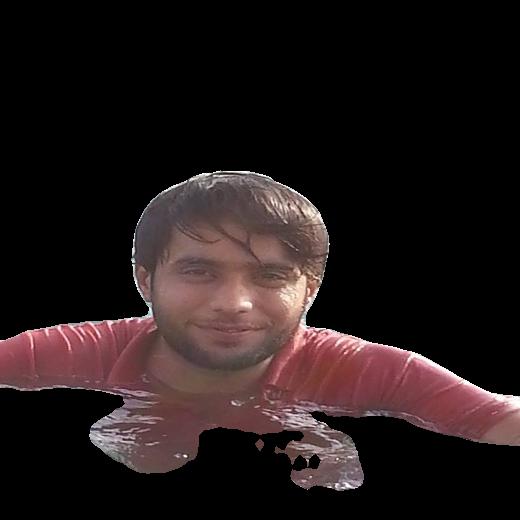

Supplement: Supplementary file 1 — Supplementary Information. [file 41598_2024_80657_MOESM1_ESM.zip › Dataset/High temperature human images/masked_10354959_762433690473974_6556596713247427865_n.jpg]

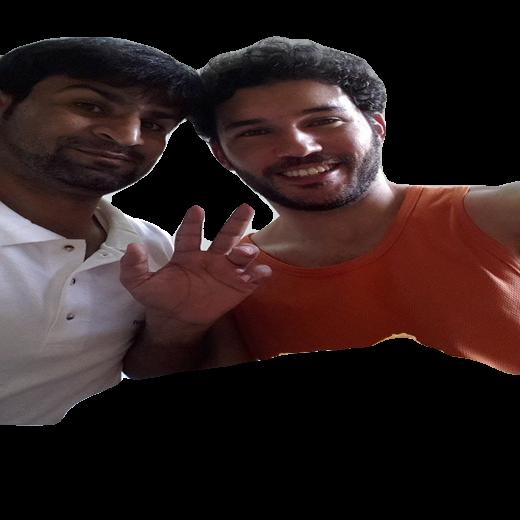

Supplement: Supplementary file 1 — Supplementary Information. [file 41598_2024_80657_MOESM1_ESM.zip › Dataset/High temperature human images/masked_10357585_789677814418072_5199014926259212415_n.jpg]

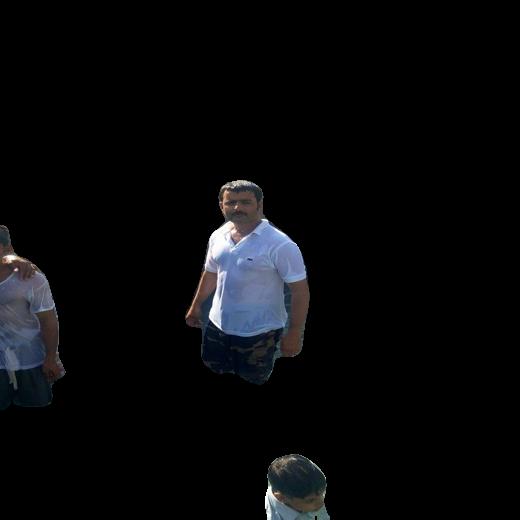

Supplement: Supplementary file 1 — Supplementary Information. [file 41598_2024_80657_MOESM1_ESM.zip › Dataset/High temperature human images/masked_10363662_690081247696067_4492261190064085886_n.jpg]

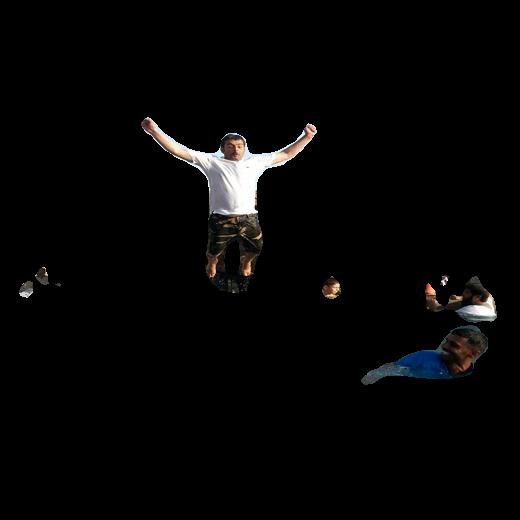

Supplement: Supplementary file 1 — Supplementary Information. [file 41598_2024_80657_MOESM1_ESM.zip › Dataset/High temperature human images/masked_10365885_762433397140670_8924675584995155760_n.jpg]

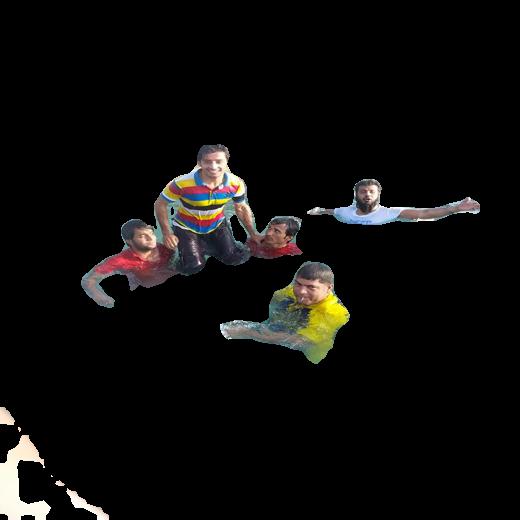

Supplement: Supplementary file 1 — Supplementary Information. [file 41598_2024_80657_MOESM1_ESM.zip › Dataset/High temperature human images/masked_10365990_762433573807319_8049692804757770213_n.jpg]

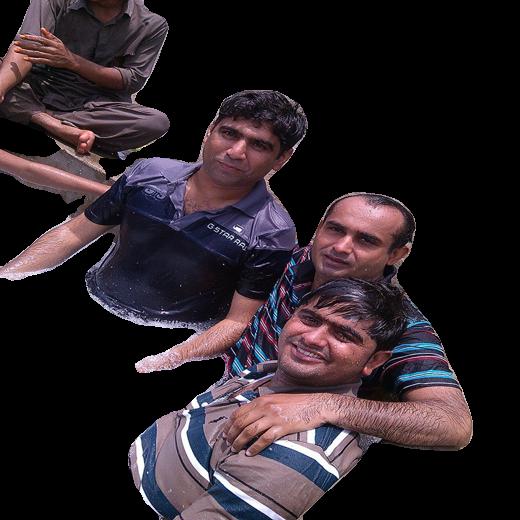

Supplement: Supplementary file 1 — Supplementary Information. [file 41598_2024_80657_MOESM1_ESM.zip › Dataset/High temperature human images/masked_10366089_752561011449266_3221807901447557231_n.jpg]

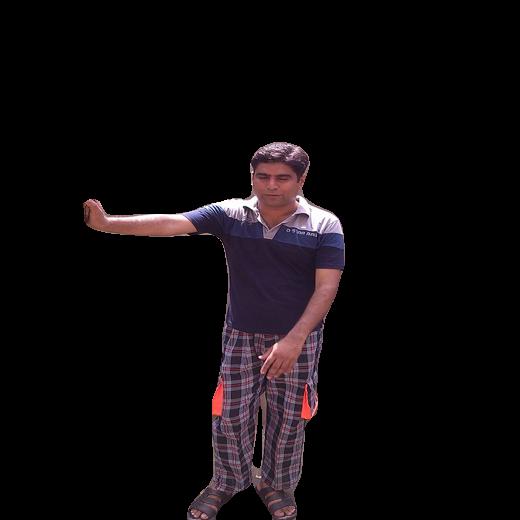

Supplement: Supplementary file 1 — Supplementary Information. [file 41598_2024_80657_MOESM1_ESM.zip › Dataset/High temperature human images/masked_10366118_752559261449441_2946128407885325932_n.jpg]

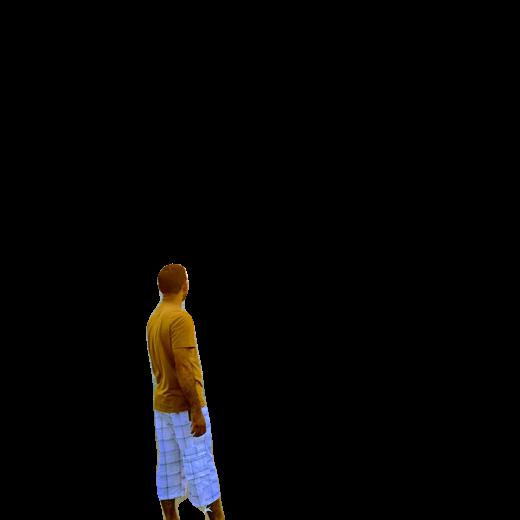

Supplement: Supplementary file 1 — Supplementary Information. [file 41598_2024_80657_MOESM1_ESM.zip › Dataset/High temperature human images/masked_103670153_1671856812966968_1787467107345495735_n.jpg]

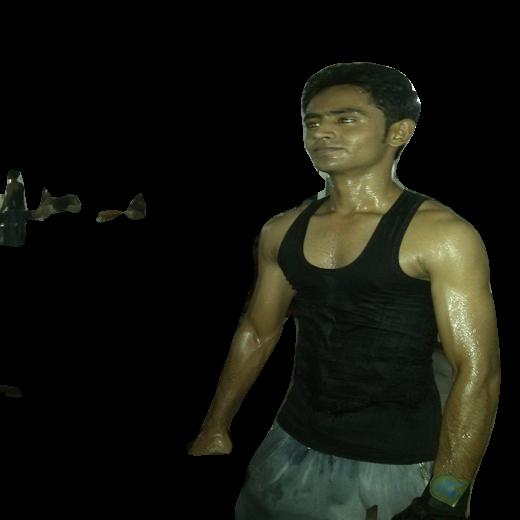

Supplement: Supplementary file 1 — Supplementary Information. [file 41598_2024_80657_MOESM1_ESM.zip › Dataset/High temperature human images/masked_10368338_591029524344626_235435207929542617_o.jpg]

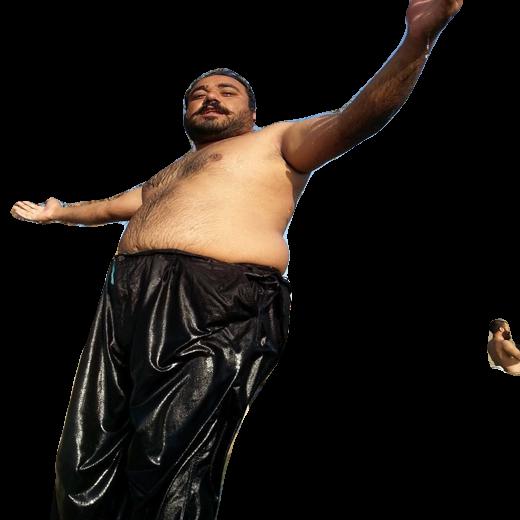

Supplement: Supplementary file 1 — Supplementary Information. [file 41598_2024_80657_MOESM1_ESM.zip › Dataset/High temperature human images/masked_10369110_762433223807354_2528666289725019596_n.jpg]

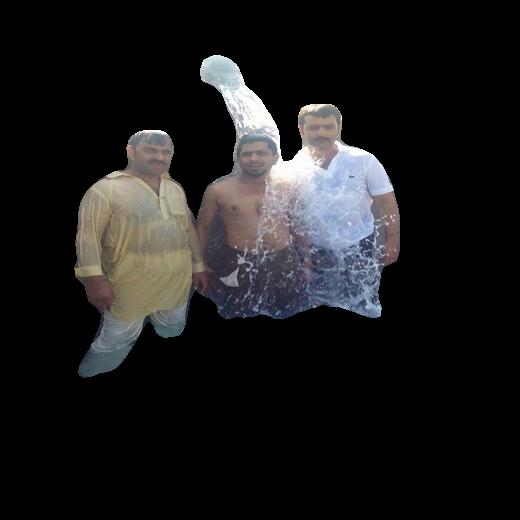

Supplement: Supplementary file 1 — Supplementary Information. [file 41598_2024_80657_MOESM1_ESM.zip › Dataset/High temperature human images/masked_10371990_690081104362748_8238652409762140890_n.jpg]

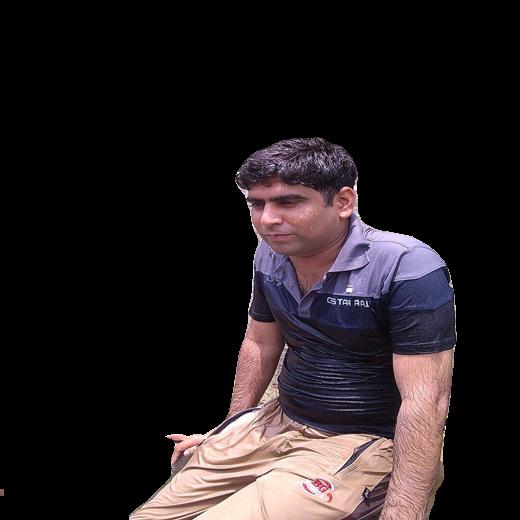

Supplement: Supplementary file 1 — Supplementary Information. [file 41598_2024_80657_MOESM1_ESM.zip › Dataset/High temperature human images/masked_10372739_752560474782653_6998039640950936219_n.jpg]

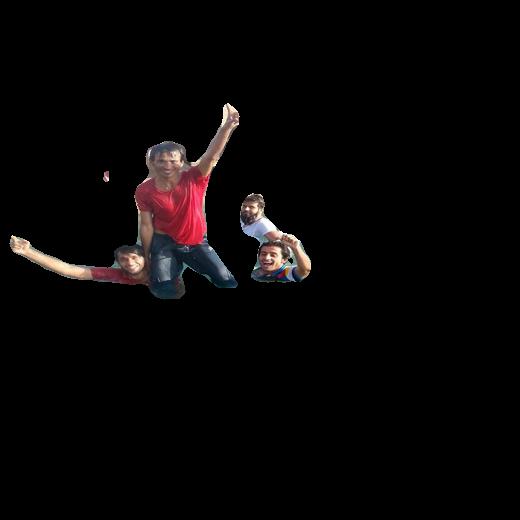

Supplement: Supplementary file 1 — Supplementary Information. [file 41598_2024_80657_MOESM1_ESM.zip › Dataset/High temperature human images/masked_10373493_762433607140649_5282473407821688690_n.jpg]

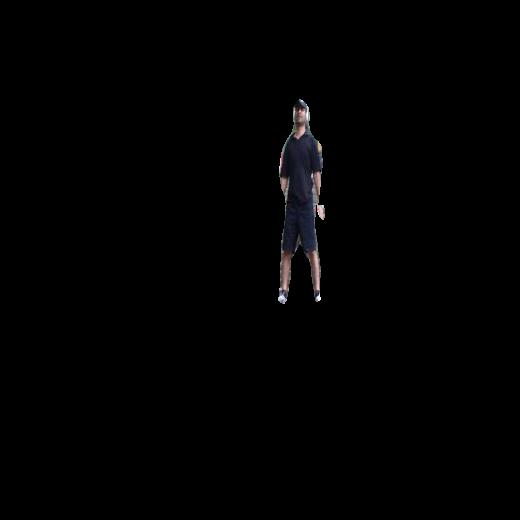

Supplement: Supplementary file 1 — Supplementary Information. [file 41598_2024_80657_MOESM1_ESM.zip › Dataset/High temperature human images/masked_10377067_911250992260753_5815151006249584047_n.jpg]

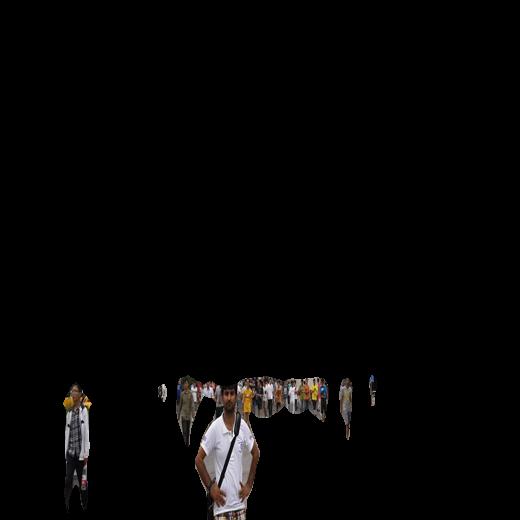

Supplement: Supplementary file 1 — Supplementary Information. [file 41598_2024_80657_MOESM1_ESM.zip › Dataset/High temperature human images/masked_10377084_789748977744289_8374946795379867958_n.jpg]

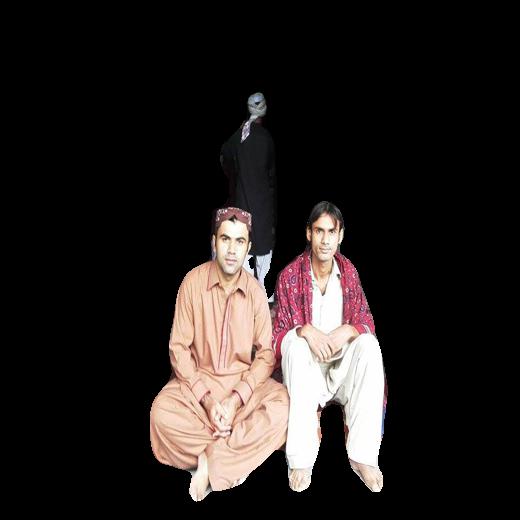

Supplement: Supplementary file 1 — Supplementary Information. [file 41598_2024_80657_MOESM1_ESM.zip › Dataset/High temperature human images/masked_10377266_908084159219298_4826505095373068041_n.jpg]

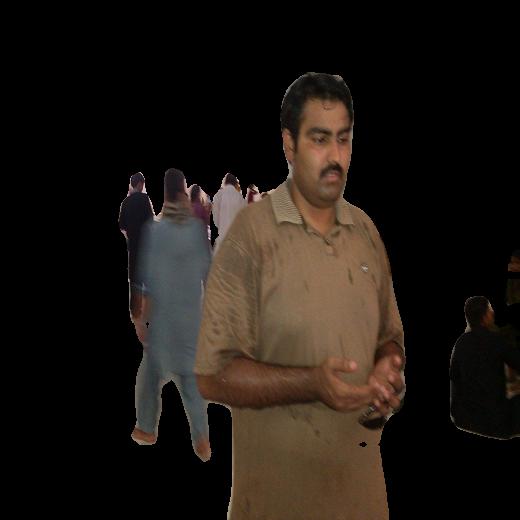

Supplement: Supplementary file 1 — Supplementary Information. [file 41598_2024_80657_MOESM1_ESM.zip › Dataset/High temperature human images/masked_10378309_752559724782728_5080884498280119987_n.jpg]

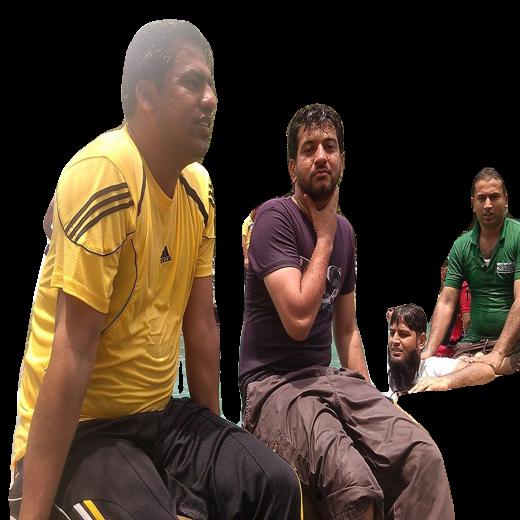

Supplement: Supplementary file 1 — Supplementary Information. [file 41598_2024_80657_MOESM1_ESM.zip › Dataset/High temperature human images/masked_10378999_752156368156397_7024535656910372959_n.jpg]

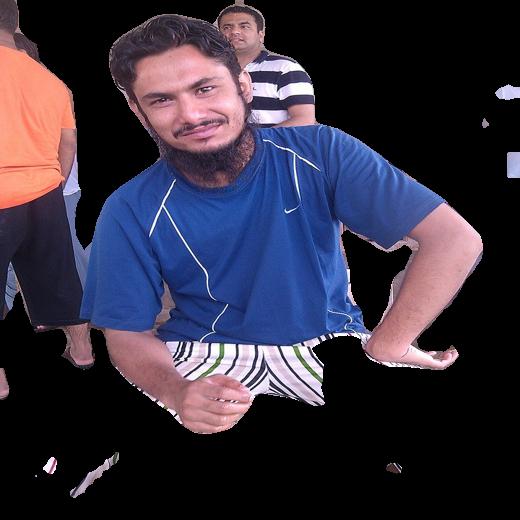

Supplement: Supplementary file 1 — Supplementary Information. [file 41598_2024_80657_MOESM1_ESM.zip › Dataset/High temperature human images/masked_10380274_752156664823034_8495827150384156443_n.jpg]

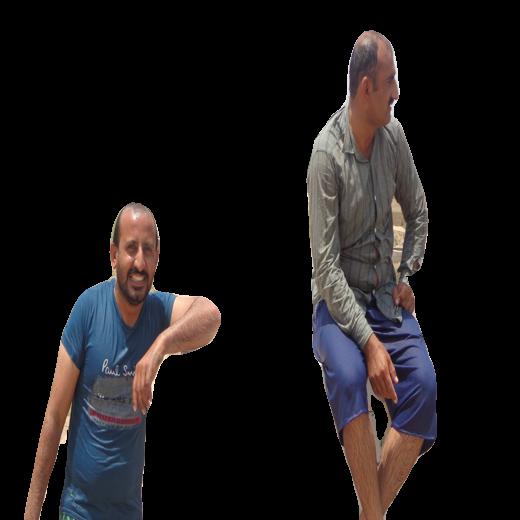

Supplement: Supplementary file 1 — Supplementary Information. [file 41598_2024_80657_MOESM1_ESM.zip › Dataset/High temperature human images/masked_10380426_725950817447973_3012406270356212517_o.jpg]

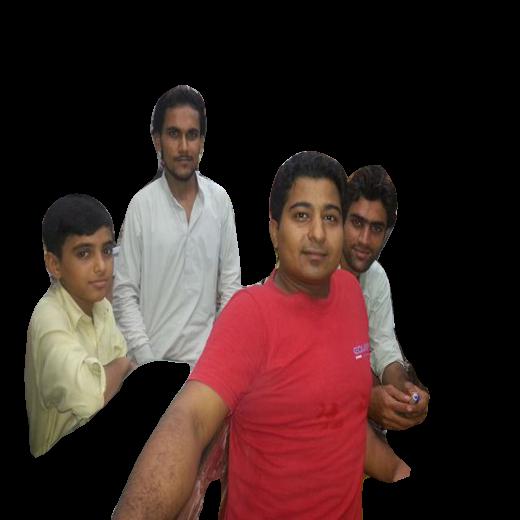

Supplement: Supplementary file 1 — Supplementary Information. [file 41598_2024_80657_MOESM1_ESM.zip › Dataset/High temperature human images/masked_10382252_690923280991908_1332170003756701127_o.jpg]

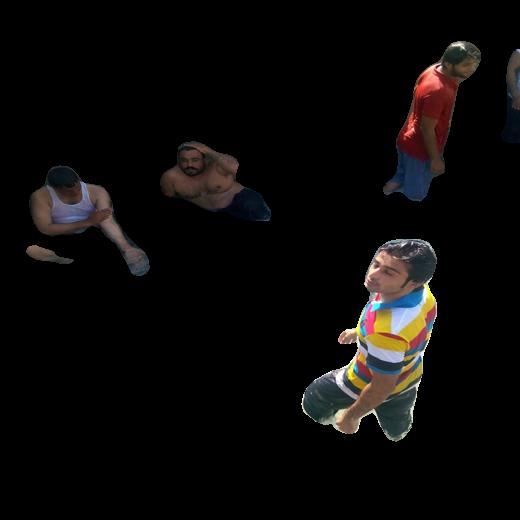

Supplement: Supplementary file 1 — Supplementary Information. [file 41598_2024_80657_MOESM1_ESM.zip › Dataset/High temperature human images/masked_10384363_690082417695950_3121084240166701655_n.jpg]

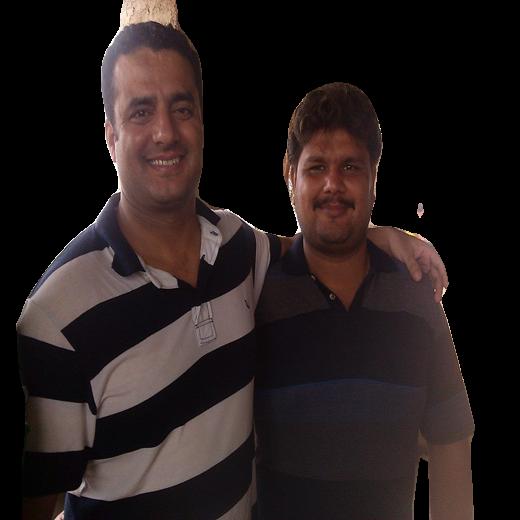

Supplement: Supplementary file 1 — Supplementary Information. [file 41598_2024_80657_MOESM1_ESM.zip › Dataset/High temperature human images/masked_10386353_752156568156377_3437440191100354309_n.jpg]

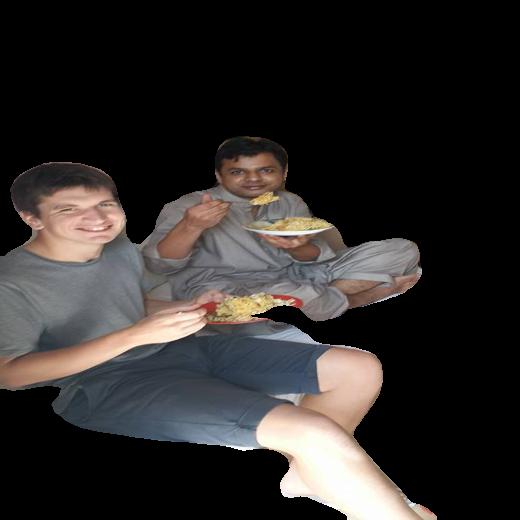

Supplement: Supplementary file 1 — Supplementary Information. [file 41598_2024_80657_MOESM1_ESM.zip › Dataset/High temperature human images/masked_10387223_801168939935626_7485563156115196872_n.jpg]

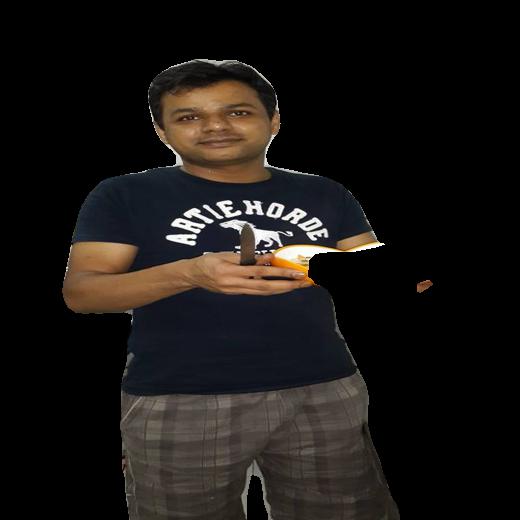

Supplement: Supplementary file 1 — Supplementary Information. [file 41598_2024_80657_MOESM1_ESM.zip › Dataset/High temperature human images/masked_10387545_801169189935601_6784162585763005609_n.jpg]

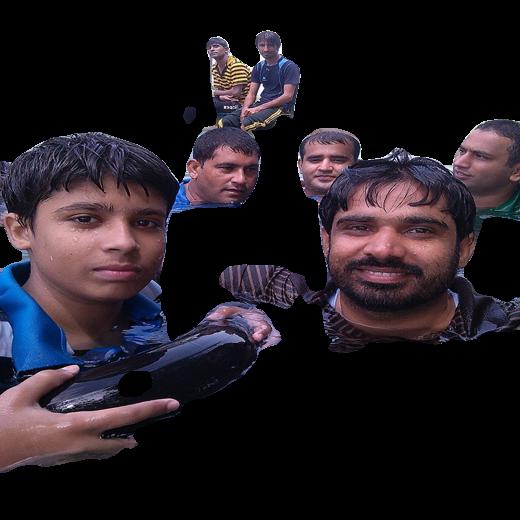

Supplement: Supplementary file 1 — Supplementary Information. [file 41598_2024_80657_MOESM1_ESM.zip › Dataset/High temperature human images/masked_10387566_752157094822991_1929486537614801292_n.jpg]

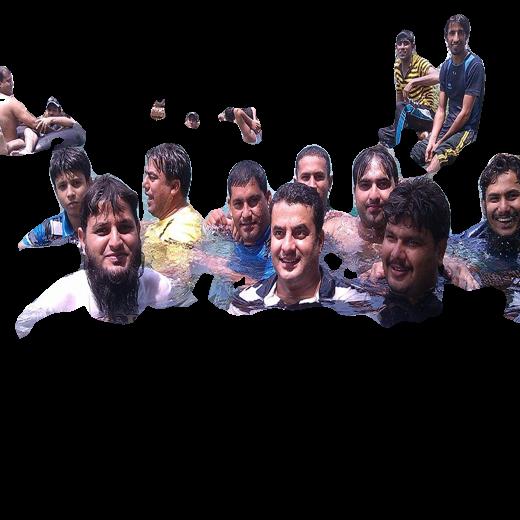

Supplement: Supplementary file 1 — Supplementary Information. [file 41598_2024_80657_MOESM1_ESM.zip › Dataset/High temperature human images/masked_10388569_751600404878660_4956191227794037014_n.jpg]

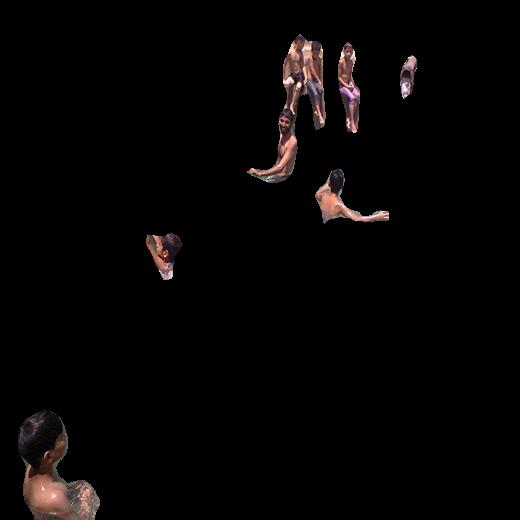

Supplement: Supplementary file 1 — Supplementary Information. [file 41598_2024_80657_MOESM1_ESM.zip › Dataset/High temperature human images/masked_10388683_811970258826713_3771260495076974629_n.jpg]

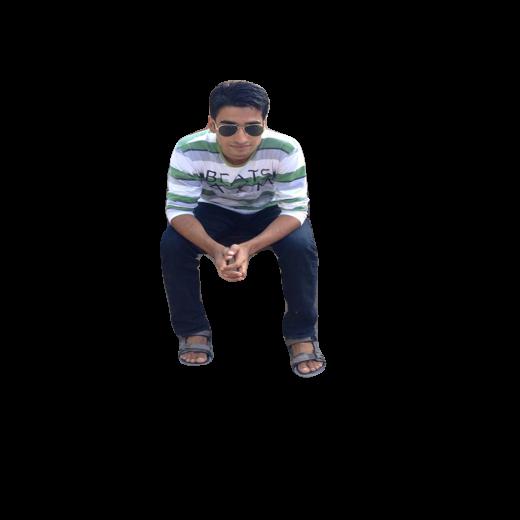

Supplement: Supplementary file 1 — Supplementary Information. [file 41598_2024_80657_MOESM1_ESM.zip › Dataset/High temperature human images/masked_10390988_608968895885358_3987960497018957545_n.jpg]

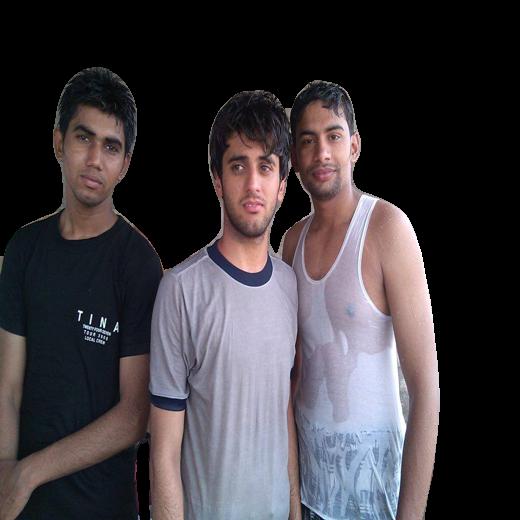

Supplement: Supplementary file 1 — Supplementary Information. [file 41598_2024_80657_MOESM1_ESM.zip › Dataset/High temperature human images/masked_10391389_666910840049895_832331268480157689_n.jpg]

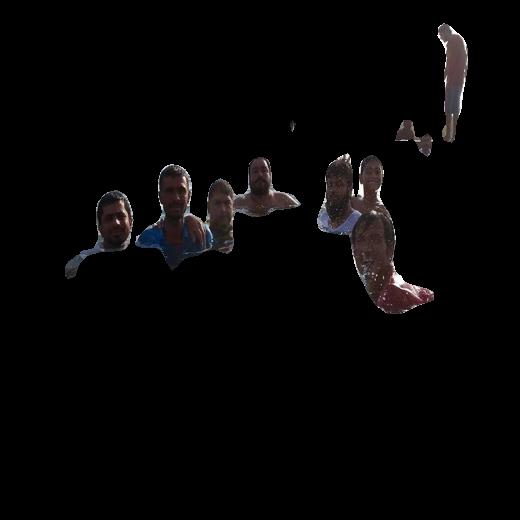

Supplement: Supplementary file 1 — Supplementary Information. [file 41598_2024_80657_MOESM1_ESM.zip › Dataset/High temperature human images/masked_10391463_762433743807302_1926099638989048100_n.jpg]

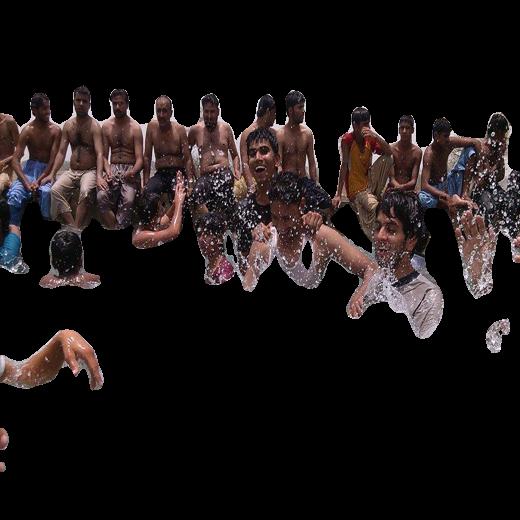

Supplement: Supplementary file 1 — Supplementary Information. [file 41598_2024_80657_MOESM1_ESM.zip › Dataset/High temperature human images/masked_10394114_666910633383249_767490953610365481_n.jpg]

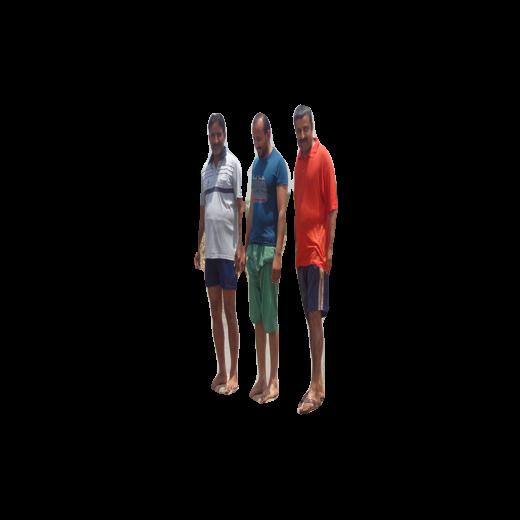

Supplement: Supplementary file 1 — Supplementary Information. [file 41598_2024_80657_MOESM1_ESM.zip › Dataset/High temperature human images/masked_10397102_725950664114655_4846285384864226843_o.jpg]

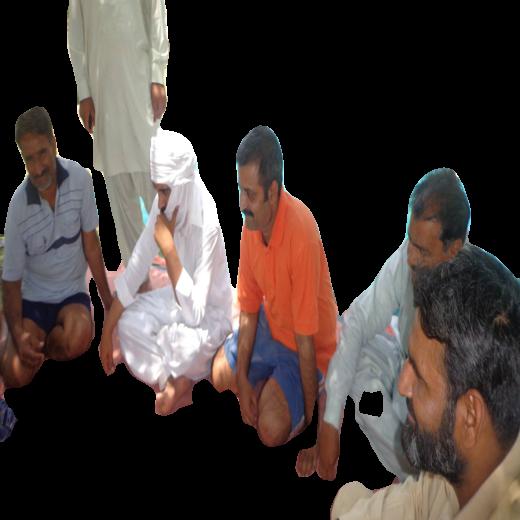

Supplement: Supplementary file 1 — Supplementary Information. [file 41598_2024_80657_MOESM1_ESM.zip › Dataset/High temperature human images/masked_10397127_725949510781437_859633059128165737_o.jpg]

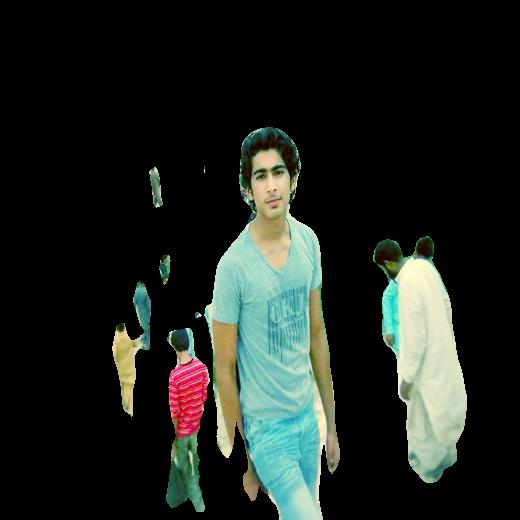

Supplement: Supplementary file 1 — Supplementary Information. [file 41598_2024_80657_MOESM1_ESM.zip › Dataset/High temperature human images/masked_10398046_882703971745761_4848563192324075896_n.jpg]

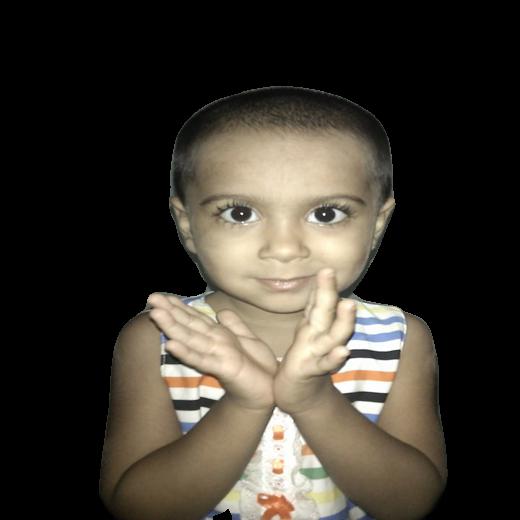

Supplement: Supplementary file 1 — Supplementary Information. [file 41598_2024_80657_MOESM1_ESM.zip › Dataset/High temperature human images/masked_10398707_739097596128941_4501908018489876219_n.jpg]

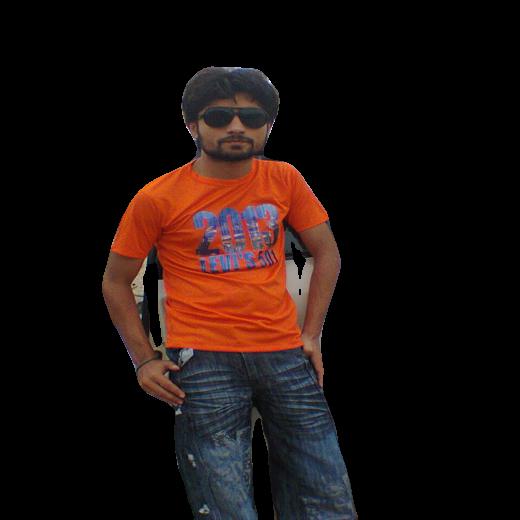

Supplement: Supplementary file 1 — Supplementary Information. [file 41598_2024_80657_MOESM1_ESM.zip › Dataset/High temperature human images/masked_1039988_482985528449784_1132263018_o.jpg]

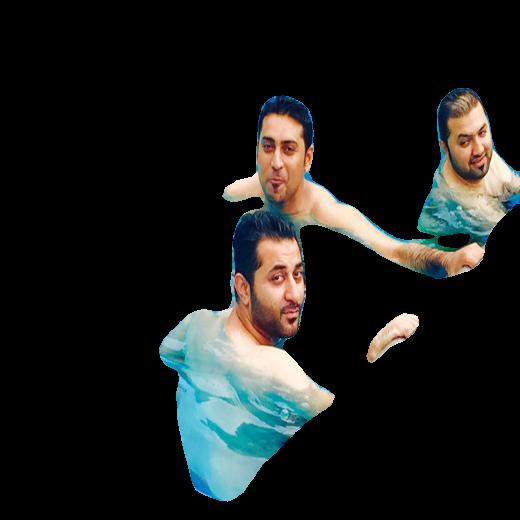

Supplement: Supplementary file 1 — Supplementary Information. [file 41598_2024_80657_MOESM1_ESM.zip › Dataset/High temperature human images/masked_10401364_879929268719297_4484524908166877298_n.jpg]

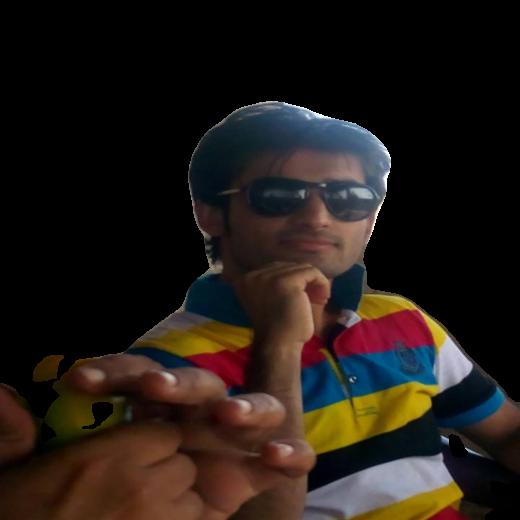

Supplement: Supplementary file 1 — Supplementary Information. [file 41598_2024_80657_MOESM1_ESM.zip › Dataset/High temperature human images/masked_10402871_690080754362783_3689709671092076108_n.jpg]

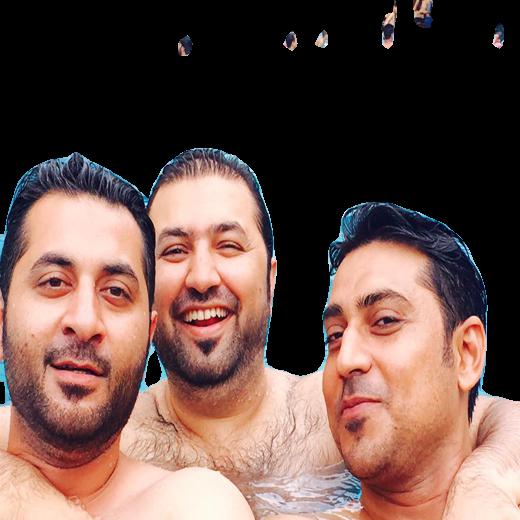

Supplement: Supplementary file 1 — Supplementary Information. [file 41598_2024_80657_MOESM1_ESM.zip › Dataset/High temperature human images/masked_10403251_879928888719335_7324985736650709046_n.jpg]

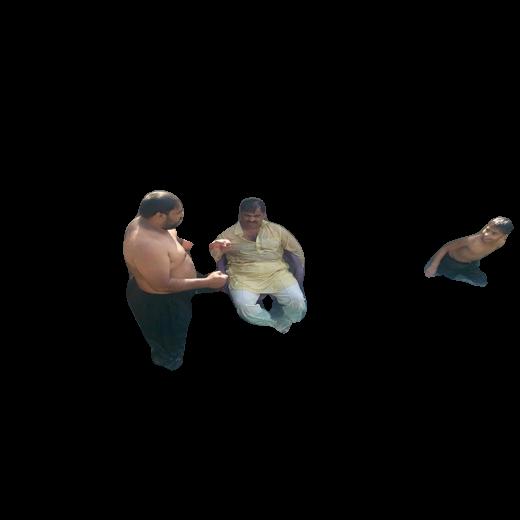

Supplement: Supplementary file 1 — Supplementary Information. [file 41598_2024_80657_MOESM1_ESM.zip › Dataset/High temperature human images/masked_10403658_690081454362713_1799633932508016586_n.jpg]

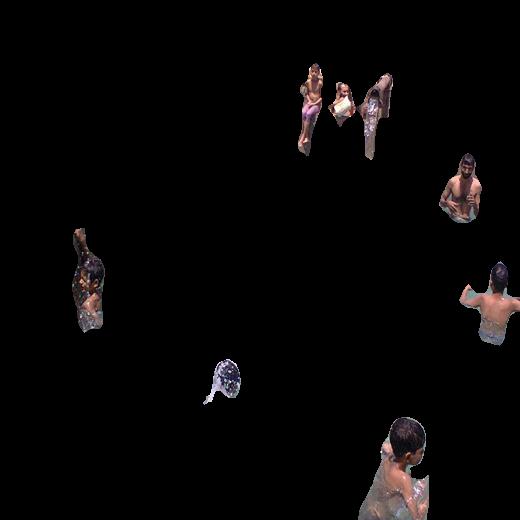

Supplement: Supplementary file 1 — Supplementary Information. [file 41598_2024_80657_MOESM1_ESM.zip › Dataset/High temperature human images/masked_10404198_811970662160006_8982281282440809274_n.jpg]

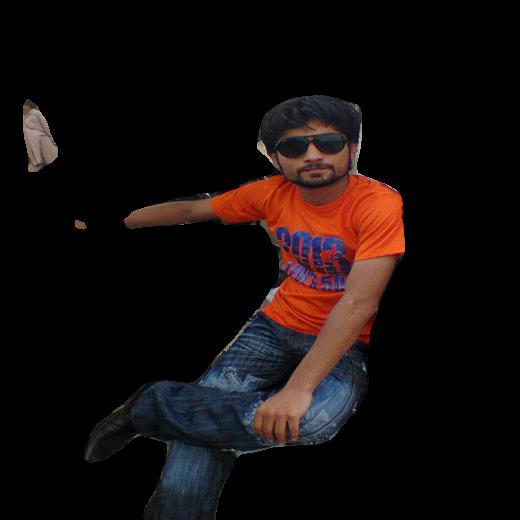

Supplement: Supplementary file 1 — Supplementary Information. [file 41598_2024_80657_MOESM1_ESM.zip › Dataset/High temperature human images/masked_1040459_482985531783117_1859635662_o.jpg]

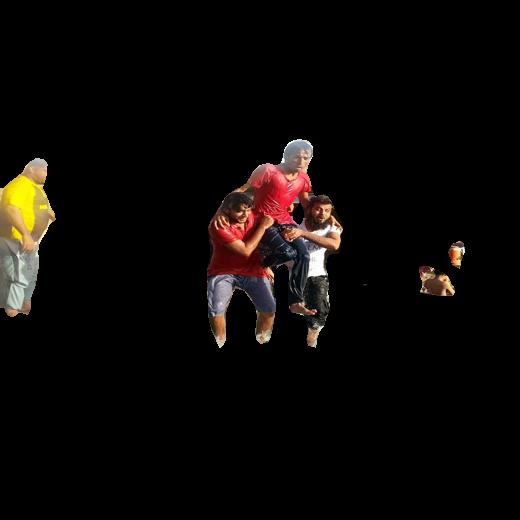

Supplement: Supplementary file 1 — Supplementary Information. [file 41598_2024_80657_MOESM1_ESM.zip › Dataset/High temperature human images/masked_10405551_762433530473990_5403883855203678564_n.jpg]

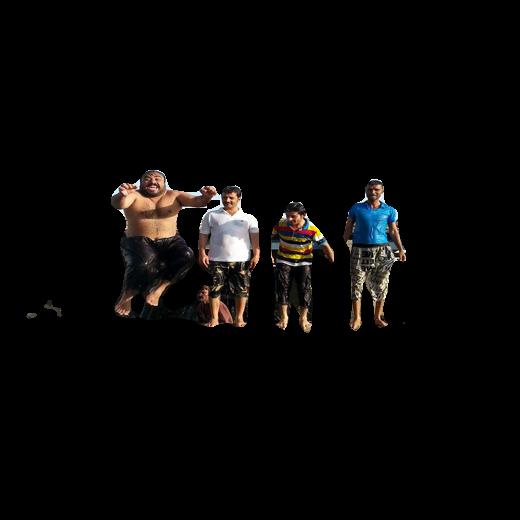

Supplement: Supplementary file 1 — Supplementary Information. [file 41598_2024_80657_MOESM1_ESM.zip › Dataset/High temperature human images/masked_10408526_762433303807346_1568953244297414598_n.jpg]

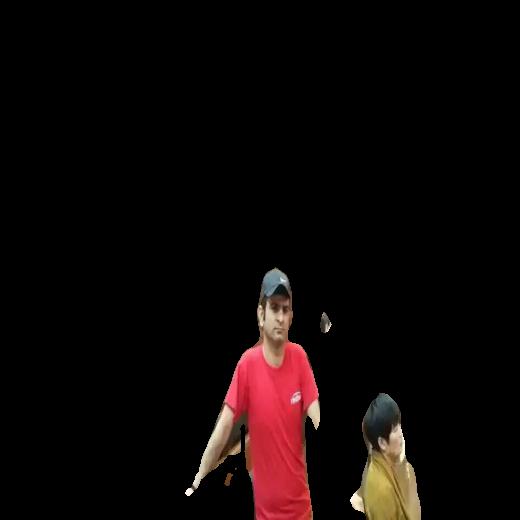

Supplement: Supplementary file 1 — Supplementary Information. [file 41598_2024_80657_MOESM1_ESM.zip › Dataset/High temperature human images/masked_10410568_921512391234613_8839364724060575905_n.jpg]

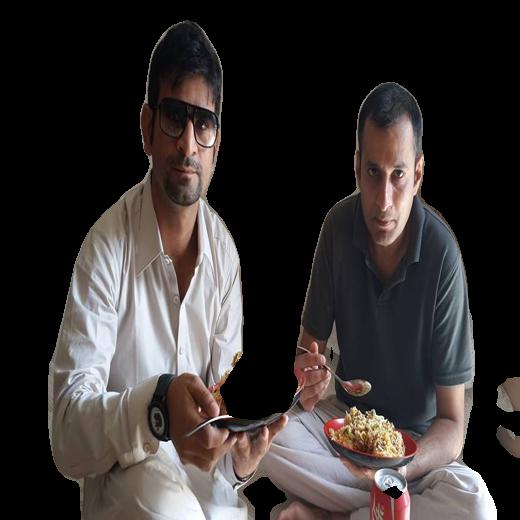

Supplement: Supplementary file 1 — Supplementary Information. [file 41598_2024_80657_MOESM1_ESM.zip › Dataset/High temperature human images/masked_10410775_801161583269695_8153383597055237544_n.jpg]

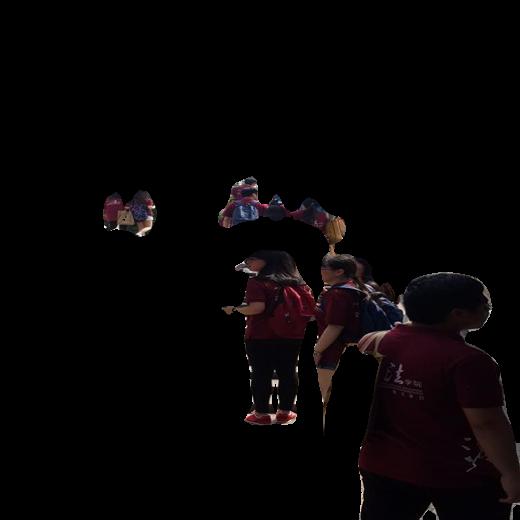

Supplement: Supplementary file 1 — Supplementary Information. [file 41598_2024_80657_MOESM1_ESM.zip › Dataset/High temperature human images/masked_10411008_956512171067968_757742966123468822_n.jpg]

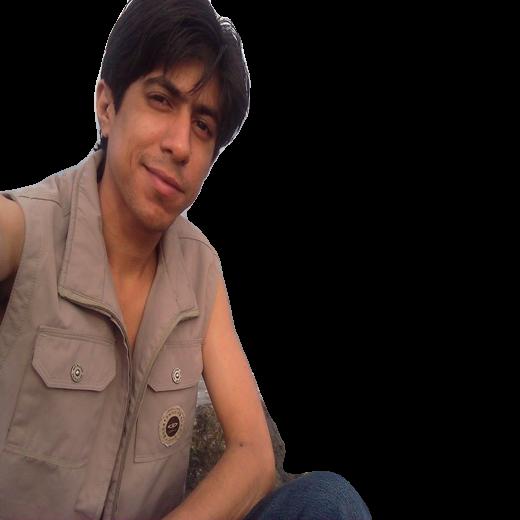

Supplement: Supplementary file 1 — Supplementary Information. [file 41598_2024_80657_MOESM1_ESM.zip › Dataset/High temperature human images/masked_10411083_746050508802133_7152451984677876042_n.jpg]

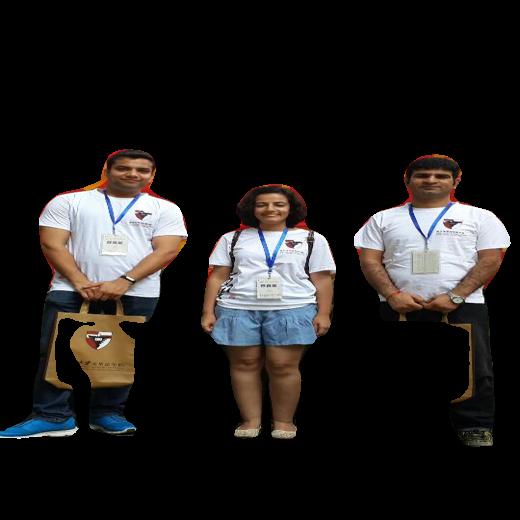

Supplement: Supplementary file 1 — Supplementary Information. [file 41598_2024_80657_MOESM1_ESM.zip › Dataset/High temperature human images/masked_10411842_955840307801821_848719595228710592_n.jpg]

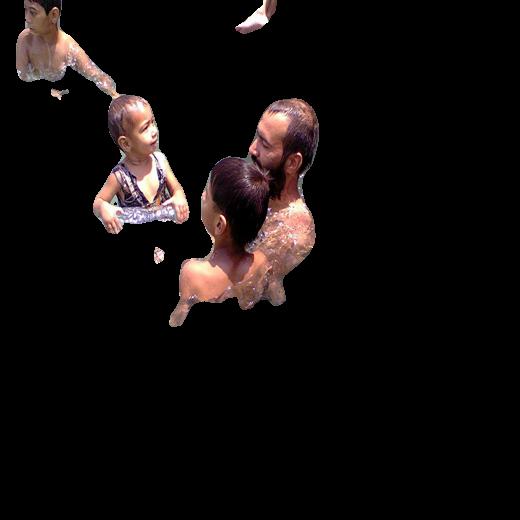

Supplement: Supplementary file 1 — Supplementary Information. [file 41598_2024_80657_MOESM1_ESM.zip › Dataset/High temperature human images/masked_10415607_811970748826664_7795099256917859777_n.jpg]

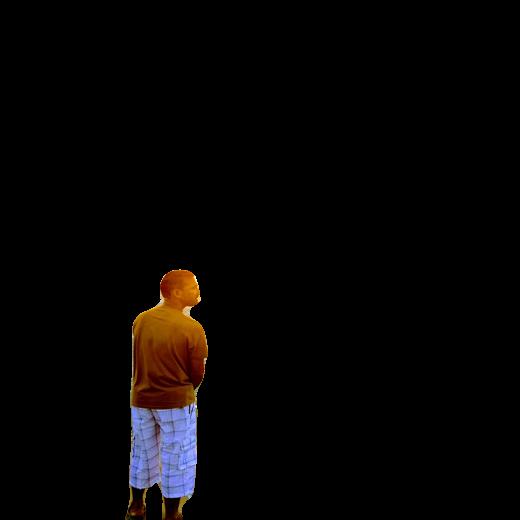

Supplement: Supplementary file 1 — Supplementary Information. [file 41598_2024_80657_MOESM1_ESM.zip › Dataset/High temperature human images/masked_104166153_1671857646300218_8431809757818905109_n.jpg]

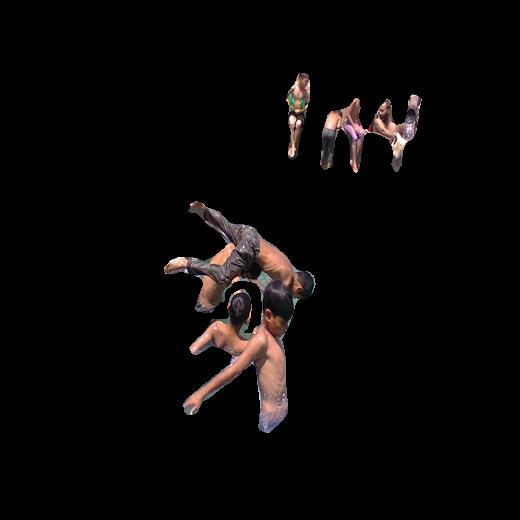

Supplement: Supplementary file 1 — Supplementary Information. [file 41598_2024_80657_MOESM1_ESM.zip › Dataset/High temperature human images/masked_10417513_811970462160026_6448252927329556536_n.jpg]

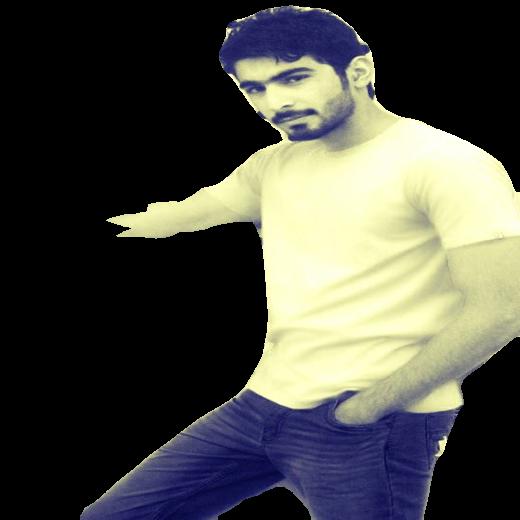

Supplement: Supplementary file 1 — Supplementary Information. [file 41598_2024_80657_MOESM1_ESM.zip › Dataset/High temperature human images/masked_10417768_1023553514327472_1953182784351418394_n.jpg]

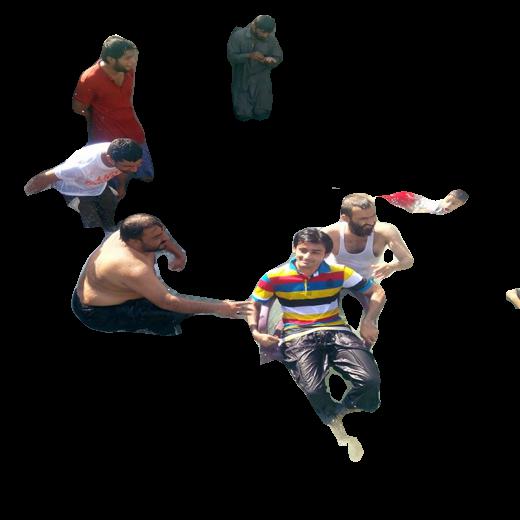

Supplement: Supplementary file 1 — Supplementary Information. [file 41598_2024_80657_MOESM1_ESM.zip › Dataset/High temperature human images/masked_10418357_690082091029316_7255931297409040133_n.jpg]

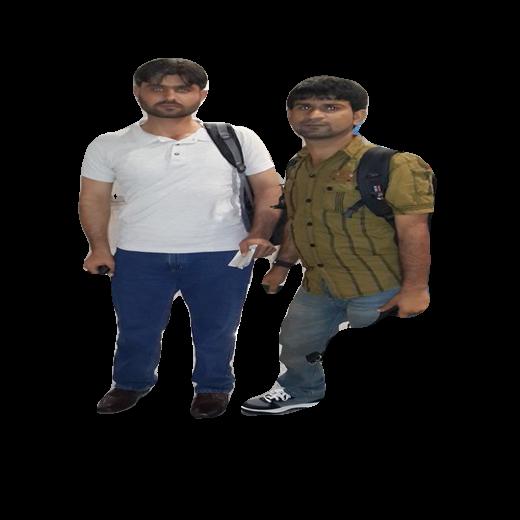

Supplement: Supplementary file 1 — Supplementary Information. [file 41598_2024_80657_MOESM1_ESM.zip › Dataset/High temperature human images/masked_10419563_788794937839693_4557059858841920029_n.jpg]

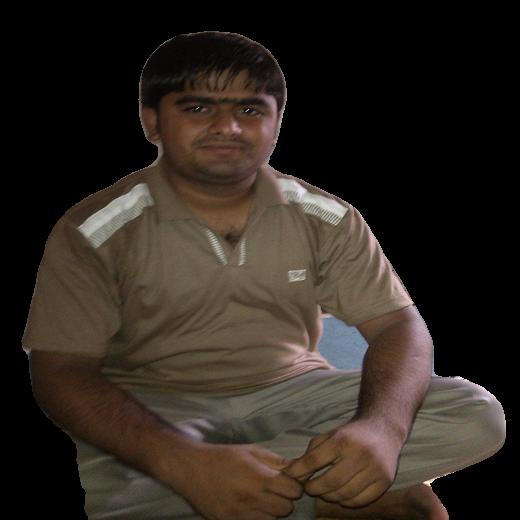

Supplement: Supplementary file 1 — Supplementary Information. [file 41598_2024_80657_MOESM1_ESM.zip › Dataset/High temperature human images/masked_10420014_750669841638383_5201072182524982836_n.jpg]

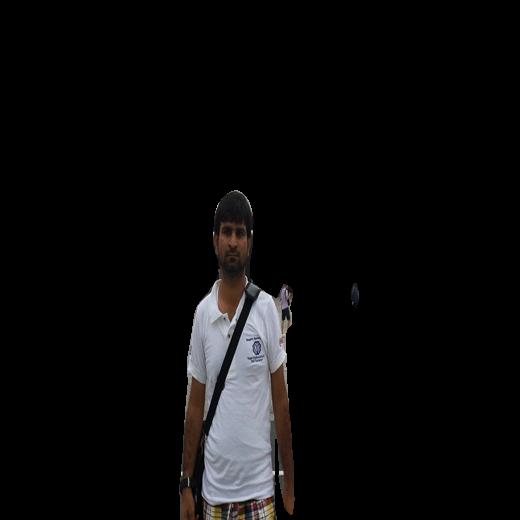

Supplement: Supplementary file 1 — Supplementary Information. [file 41598_2024_80657_MOESM1_ESM.zip › Dataset/High temperature human images/masked_10420087_789750144410839_6463154516600832038_n (1).jpg]

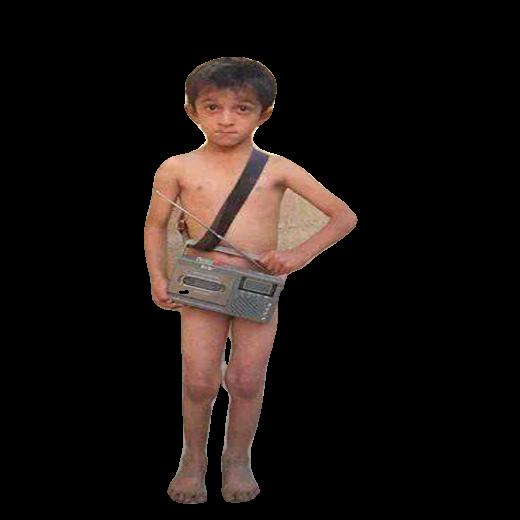

Supplement: Supplementary file 1 — Supplementary Information. [file 41598_2024_80657_MOESM1_ESM.zip › Dataset/High temperature human images/masked_10421176_860444937341359_3060202390941258717_n.jpg]

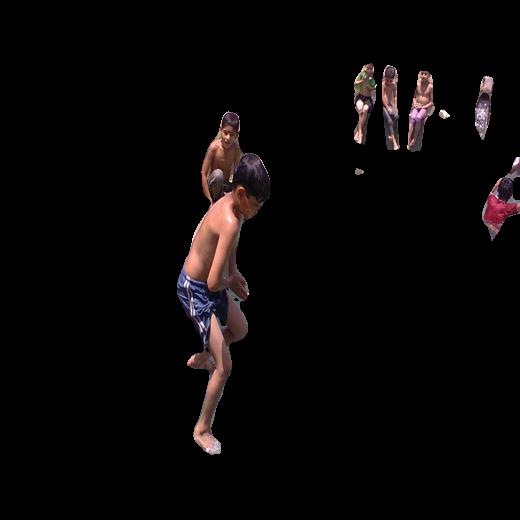

Supplement: Supplementary file 1 — Supplementary Information. [file 41598_2024_80657_MOESM1_ESM.zip › Dataset/High temperature human images/masked_10421620_811970775493328_1654101293320515417_n.jpg]

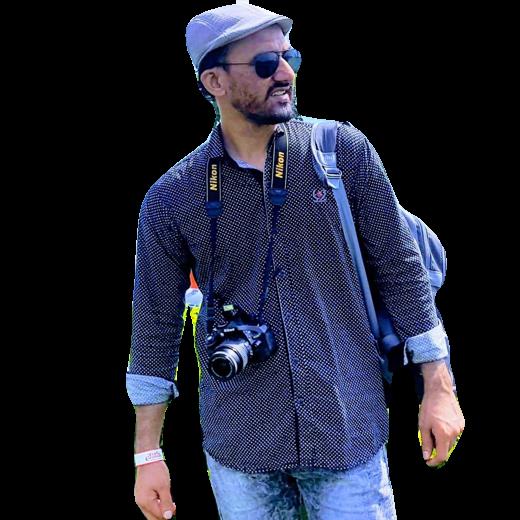

Supplement: Supplementary file 1 — Supplementary Information. [file 41598_2024_80657_MOESM1_ESM.zip › Dataset/High temperature human images/masked_104218867_1675740462578603_5426802094401479768_n.jpg]

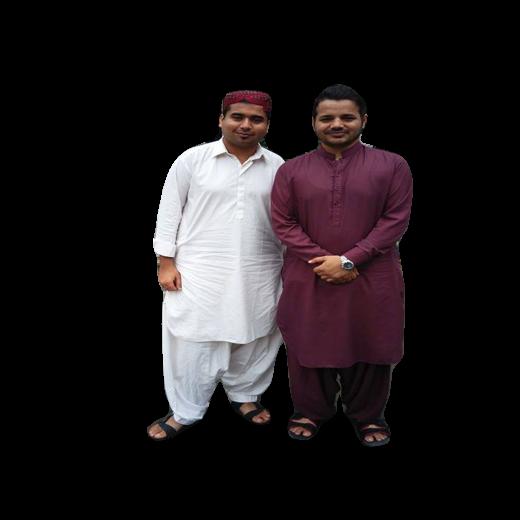

Supplement: Supplementary file 1 — Supplementary Information. [file 41598_2024_80657_MOESM1_ESM.zip › Dataset/High temperature human images/masked_10426908_908086439219070_2703334516942423082_n.jpg]

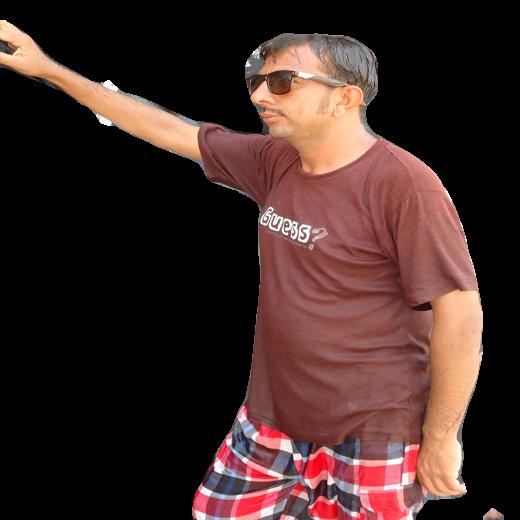

Supplement: Supplementary file 1 — Supplementary Information. [file 41598_2024_80657_MOESM1_ESM.zip › Dataset/High temperature human images/masked_10431215_1490641817875134_253415242885599918_o.jpg]

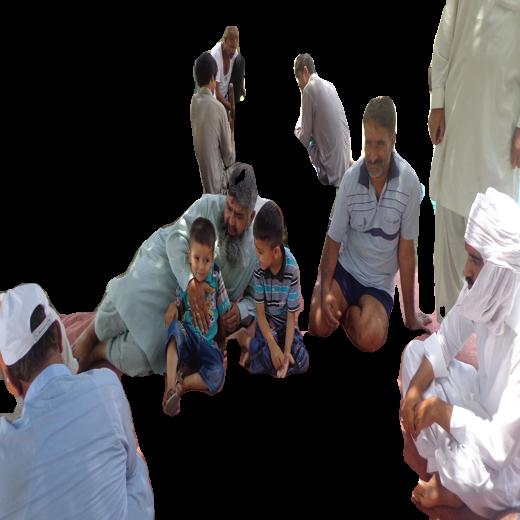

Supplement: Supplementary file 1 — Supplementary Information. [file 41598_2024_80657_MOESM1_ESM.zip › Dataset/High temperature human images/masked_10431364_725949700781418_3315450312805827546_o.jpg]

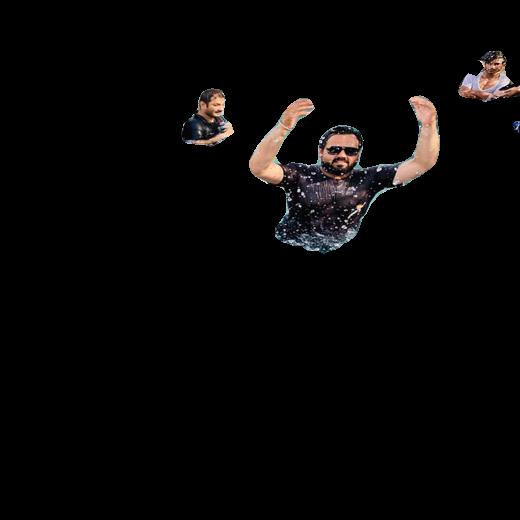

Supplement: Supplementary file 1 — Supplementary Information. [file 41598_2024_80657_MOESM1_ESM.zip › Dataset/High temperature human images/masked_104384028_1186144365063278_4530931589806244133_n.jpg]

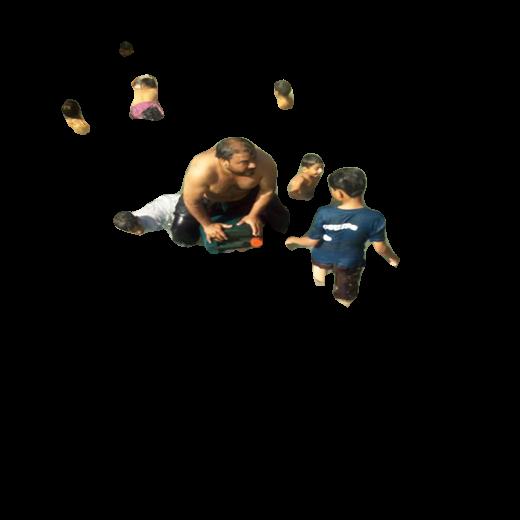

Supplement: Supplementary file 1 — Supplementary Information. [file 41598_2024_80657_MOESM1_ESM.zip › Dataset/High temperature human images/masked_10440222_690081647696027_6094855040482230859_n.jpg]

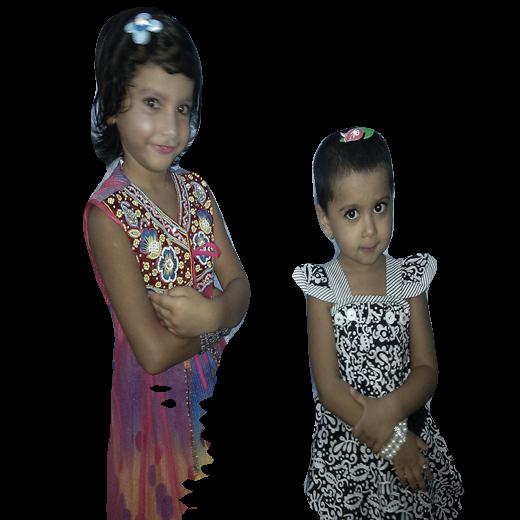

Supplement: Supplementary file 1 — Supplementary Information. [file 41598_2024_80657_MOESM1_ESM.zip › Dataset/High temperature human images/masked_10441020_749807338391300_8617008859109874827_n.jpg]

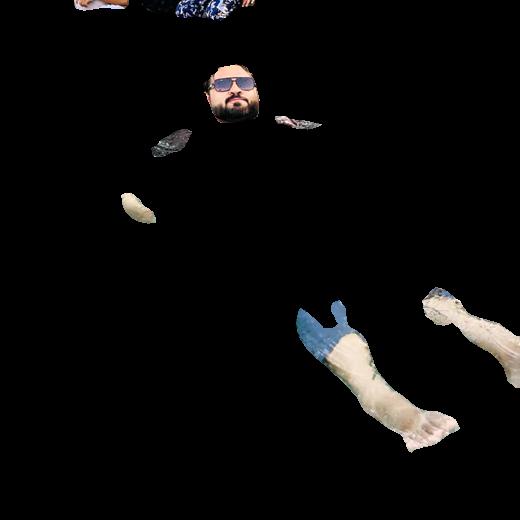

Supplement: Supplementary file 1 — Supplementary Information. [file 41598_2024_80657_MOESM1_ESM.zip › Dataset/High temperature human images/masked_104429526_1186143115063403_1644296110431012760_n.jpg]

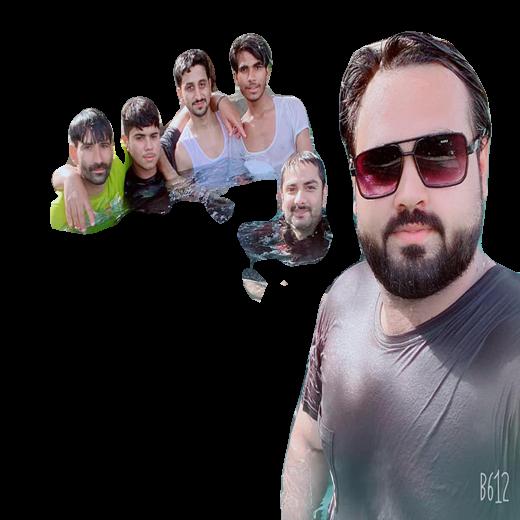

Supplement: Supplementary file 1 — Supplementary Information. [file 41598_2024_80657_MOESM1_ESM.zip › Dataset/High temperature human images/masked_104432862_1186143755063339_6177433934601153362_n.jpg]

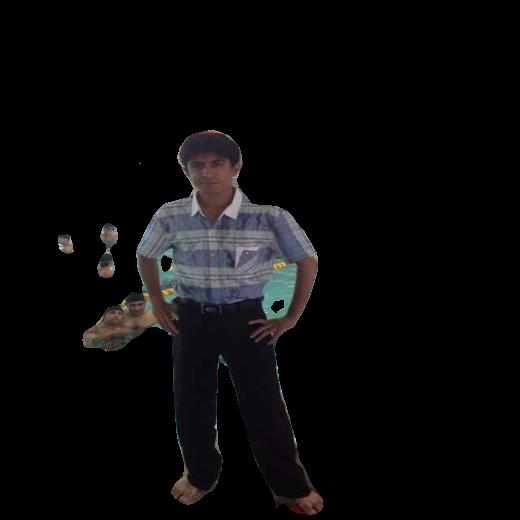

Supplement: Supplementary file 1 — Supplementary Information. [file 41598_2024_80657_MOESM1_ESM.zip › Dataset/High temperature human images/masked_10443412_712395892167595_2573267600840411076_n.jpg]

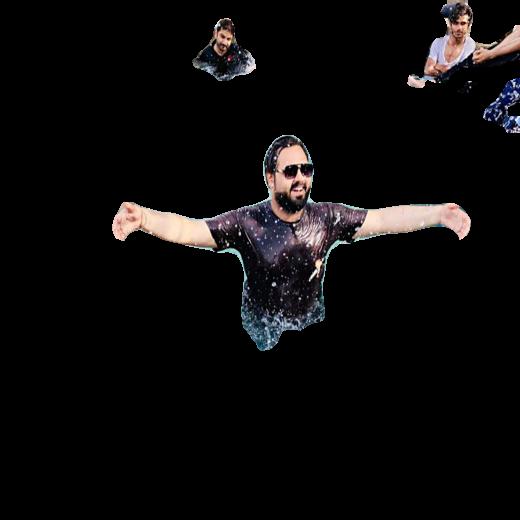

Supplement: Supplementary file 1 — Supplementary Information. [file 41598_2024_80657_MOESM1_ESM.zip › Dataset/High temperature human images/masked_104438865_1186143511730030_3872867803937791864_n.jpg]

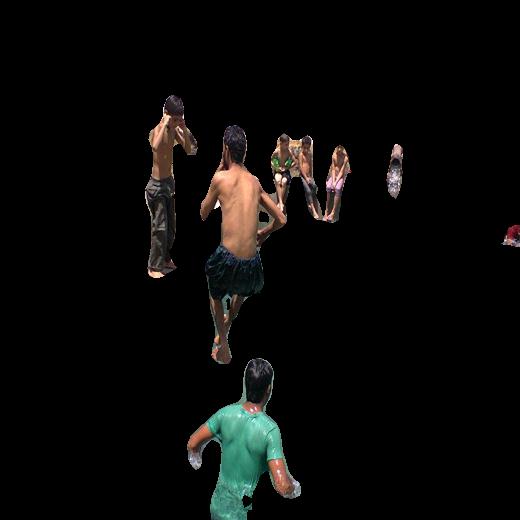

Supplement: Supplementary file 1 — Supplementary Information. [file 41598_2024_80657_MOESM1_ESM.zip › Dataset/High temperature human images/masked_10444539_811969942160078_1854277429440132241_n.jpg]

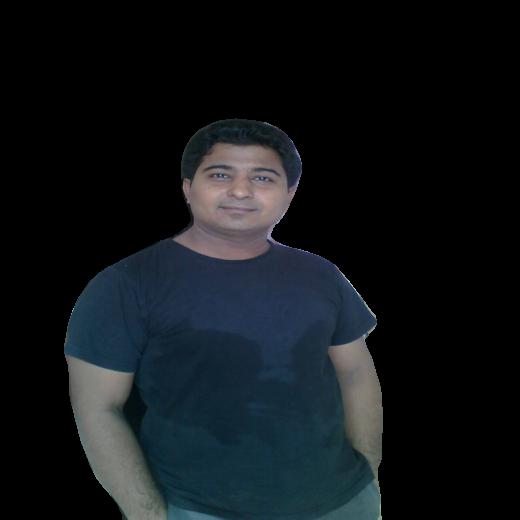

Supplement: Supplementary file 1 — Supplementary Information. [file 41598_2024_80657_MOESM1_ESM.zip › Dataset/High temperature human images/masked_10446101_690921977658705_2802173064844017315_o.jpg]

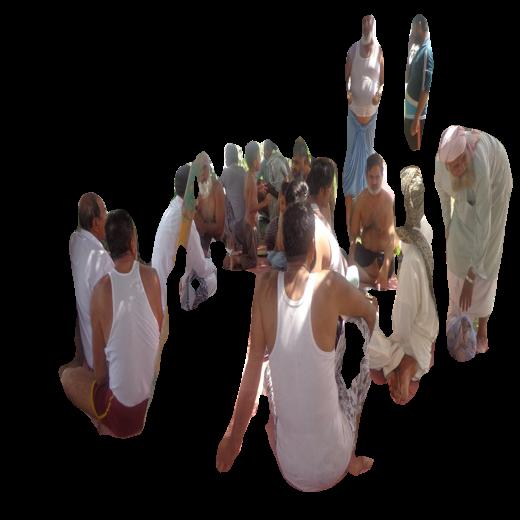

Supplement: Supplementary file 1 — Supplementary Information. [file 41598_2024_80657_MOESM1_ESM.zip › Dataset/High temperature human images/masked_10446332_725951457447909_2719864525693650966_o.jpg]

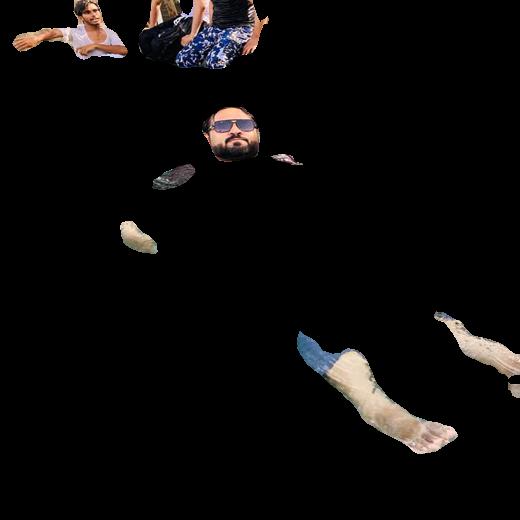

Supplement: Supplementary file 1 — Supplementary Information. [file 41598_2024_80657_MOESM1_ESM.zip › Dataset/High temperature human images/masked_104477039_1186144295063285_8151087417520123001_n.jpg]

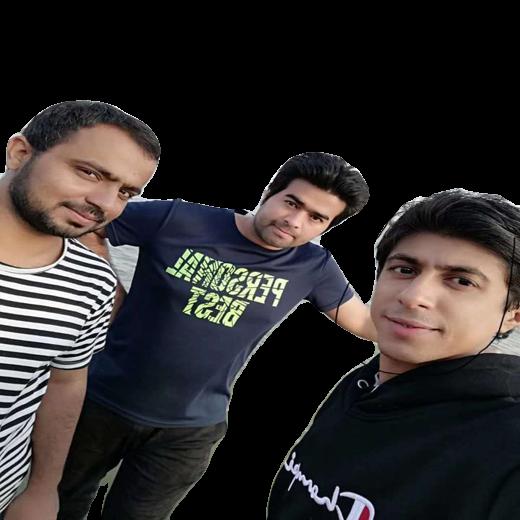

Supplement: Supplementary file 1 — Supplementary Information. [file 41598_2024_80657_MOESM1_ESM.zip › Dataset/High temperature human images/masked_104489128_2606961306211987_8042084262098040794_n.jpg]

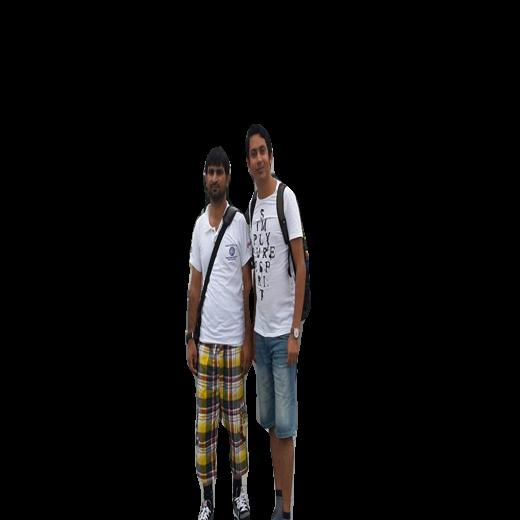

Supplement: Supplementary file 1 — Supplementary Information. [file 41598_2024_80657_MOESM1_ESM.zip › Dataset/High temperature human images/masked_10450174_789749734410880_3522390344392917082_n.jpg]

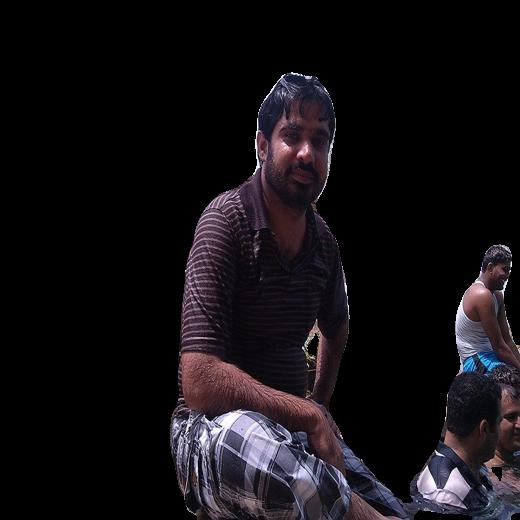

Supplement: Supplementary file 1 — Supplementary Information. [file 41598_2024_80657_MOESM1_ESM.zip › Dataset/High temperature human images/masked_10450556_752156988156335_5258795335164996069_n.jpg]

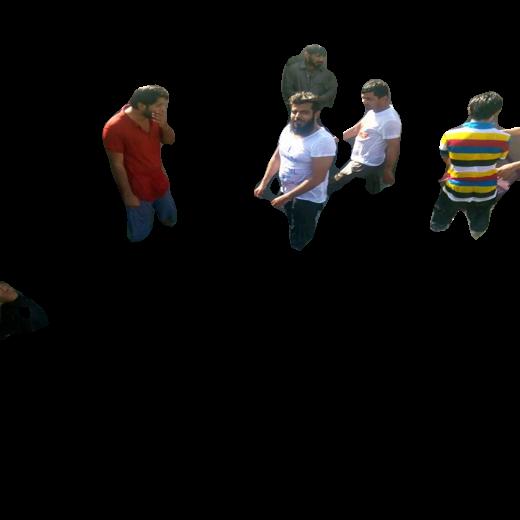

Supplement: Supplementary file 1 — Supplementary Information. [file 41598_2024_80657_MOESM1_ESM.zip › Dataset/High temperature human images/masked_10452389_690082244362634_1841858327378349787_n.jpg]

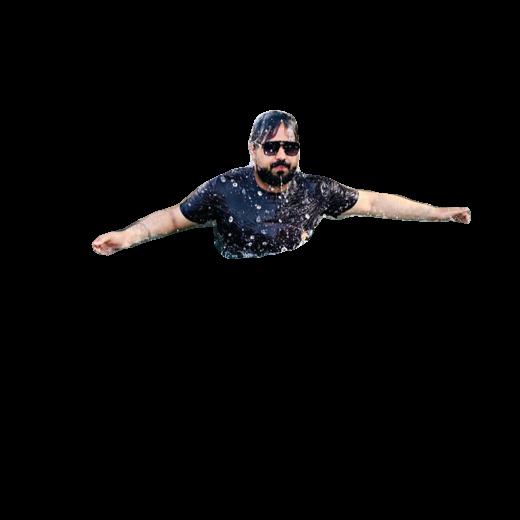

Supplement: Supplementary file 1 — Supplementary Information. [file 41598_2024_80657_MOESM1_ESM.zip › Dataset/High temperature human images/masked_104544201_1186143321730049_6084034674492307248_n.jpg]

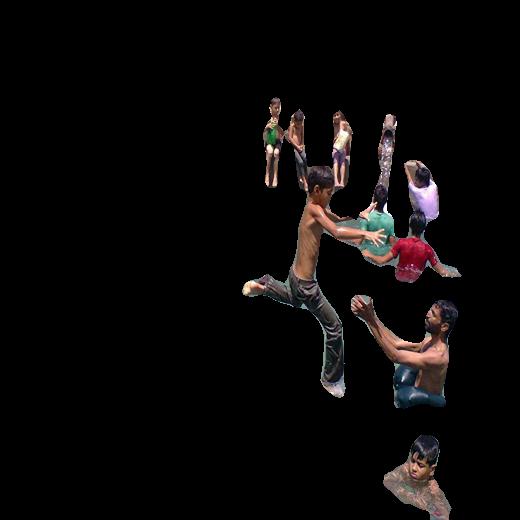

Supplement: Supplementary file 1 — Supplementary Information. [file 41598_2024_80657_MOESM1_ESM.zip › Dataset/High temperature human images/masked_10455443_811970555493350_4103270926088159137_n.jpg]

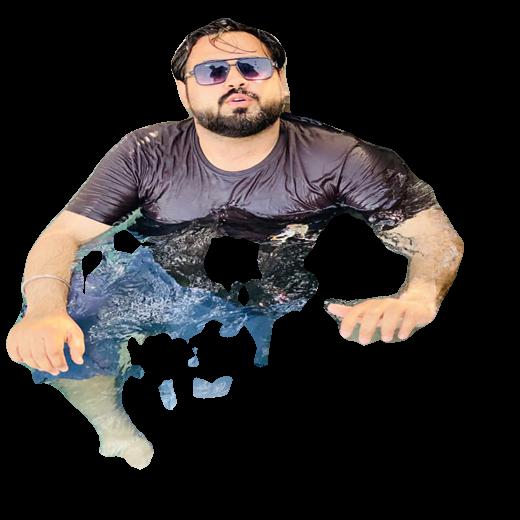

Supplement: Supplementary file 1 — Supplementary Information. [file 41598_2024_80657_MOESM1_ESM.zip › Dataset/High temperature human images/masked_104563646_1186143581730023_396857923997095292_n.jpg]

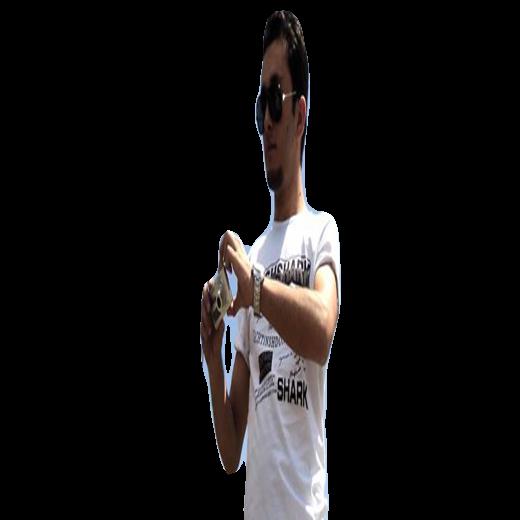

Supplement: Supplementary file 1 — Supplementary Information. [file 41598_2024_80657_MOESM1_ESM.zip › Dataset/High temperature human images/masked_10457184_805599039474804_3038446765694156845_n.jpg]

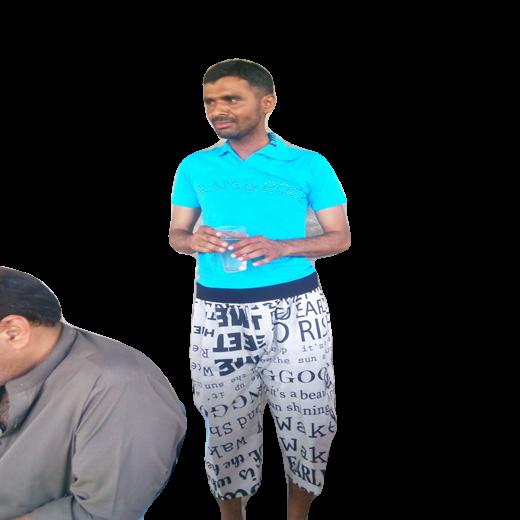

Supplement: Supplementary file 1 — Supplementary Information. [file 41598_2024_80657_MOESM1_ESM.zip › Dataset/High temperature human images/masked_10457963_690082331029292_914786788929838876_n.jpg]

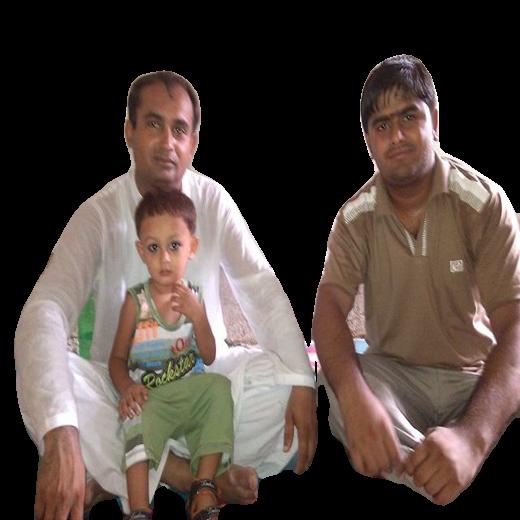

Supplement: Supplementary file 1 — Supplementary Information. [file 41598_2024_80657_MOESM1_ESM.zip › Dataset/High temperature human images/masked_10458076_752561274782573_2005250270564892073_n.jpg]

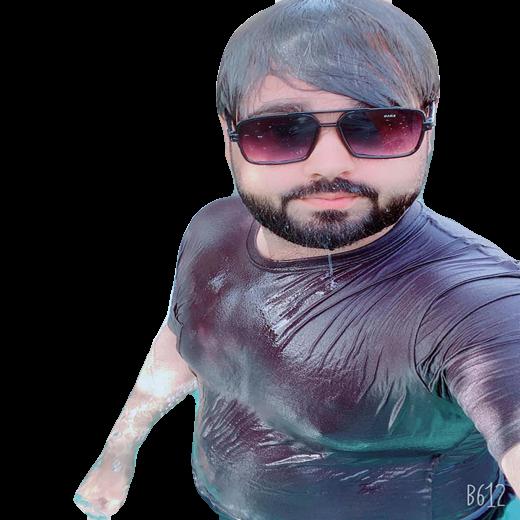

Supplement: Supplementary file 1 — Supplementary Information. [file 41598_2024_80657_MOESM1_ESM.zip › Dataset/High temperature human images/masked_104586588_1186144141729967_2071274294218150514_n.jpg]

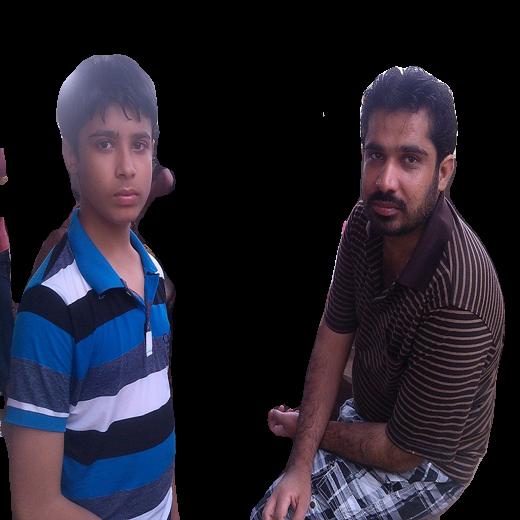

Supplement: Supplementary file 1 — Supplementary Information. [file 41598_2024_80657_MOESM1_ESM.zip › Dataset/High temperature human images/masked_10460259_751603021545065_8337076667290026127_n.jpg]

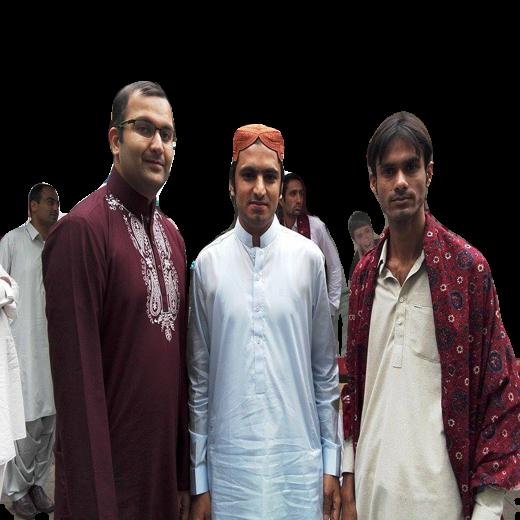

Supplement: Supplementary file 1 — Supplementary Information. [file 41598_2024_80657_MOESM1_ESM.zip › Dataset/High temperature human images/masked_10461972_908084229219291_6359018413292929921_n.jpg]

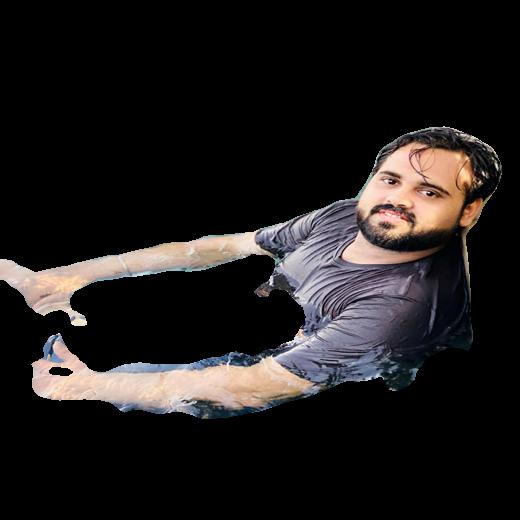

Supplement: Supplementary file 1 — Supplementary Information. [file 41598_2024_80657_MOESM1_ESM.zip › Dataset/High temperature human images/masked_104645426_1186143448396703_1402808956121627549_n.jpg]

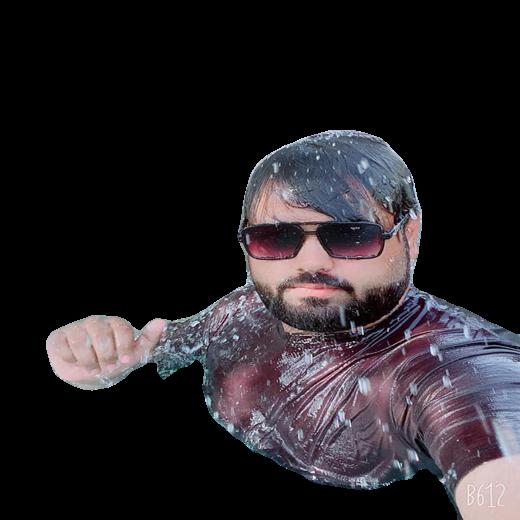

Supplement: Supplementary file 1 — Supplementary Information. [file 41598_2024_80657_MOESM1_ESM.zip › Dataset/High temperature human images/masked_104666218_1186144191729962_5487619782170471317_n.jpg]
