# Supplementary figures and images for: A deep transfer learning based convolution neural network framework for air temperature classification using human clothing images (part 1 of 2)
Source: Sci Rep. 2024 Dec 30;14:31658. doi: 10.1038/s41598-024-80657-y (PMC11685903; doi:10.1038/s41598-024-80657-y)

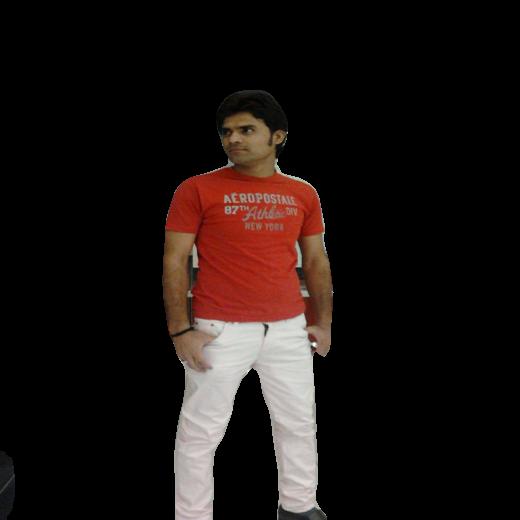

Supplement: Supplementary file 1 — Supplementary Information. [file 41598_2024_80657_MOESM1_ESM.zip › Dataset/High temperature human images/masked_10001051_690479744338568_3460252984618739129_o.jpg]

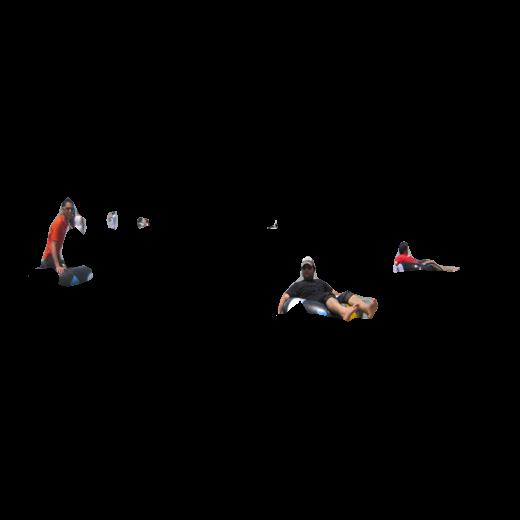

Supplement: Supplementary file 1 — Supplementary Information. [file 41598_2024_80657_MOESM1_ESM.zip › Dataset/High temperature human images/masked_10001061_623836004357379_3258080387175787112_o.jpg]

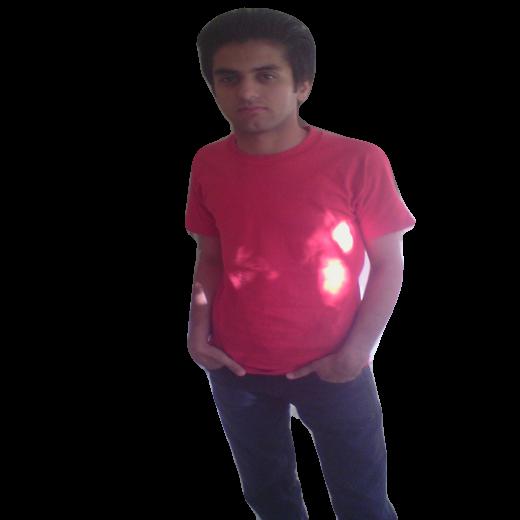

Supplement: Supplementary file 1 — Supplementary Information. [file 41598_2024_80657_MOESM1_ESM.zip › Dataset/High temperature human images/masked_10001262_614601928614120_945389631_o.jpg]

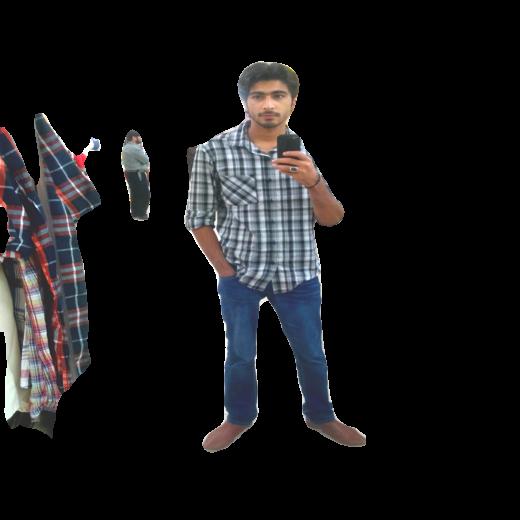

Supplement: Supplementary file 1 — Supplementary Information. [file 41598_2024_80657_MOESM1_ESM.zip › Dataset/High temperature human images/masked_10003674_996225863726904_6974160993899296168_o.jpg]

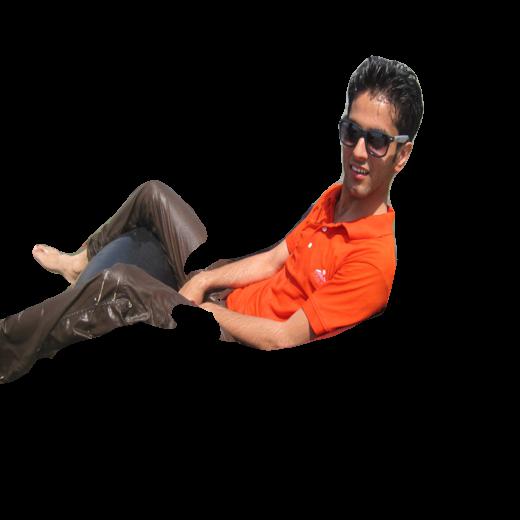

Supplement: Supplementary file 1 — Supplementary Information. [file 41598_2024_80657_MOESM1_ESM.zip › Dataset/High temperature human images/masked_10003689_623833817690931_5223359409259325371_o.jpg]

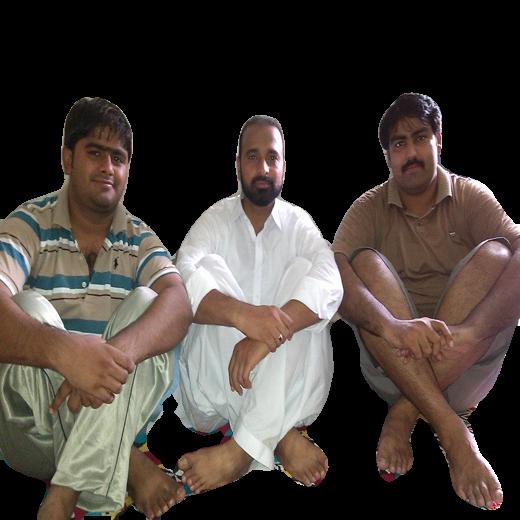

Supplement: Supplementary file 1 — Supplementary Information. [file 41598_2024_80657_MOESM1_ESM.zip › Dataset/High temperature human images/masked_10003878_752561181449249_2892024990516399014_n.jpg]

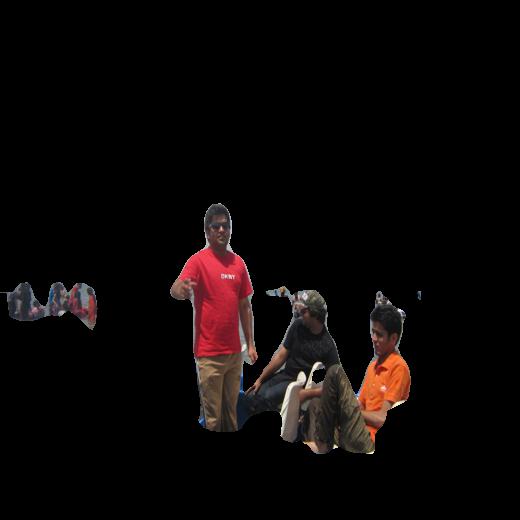

Supplement: Supplementary file 1 — Supplementary Information. [file 41598_2024_80657_MOESM1_ESM.zip › Dataset/High temperature human images/masked_10005815_623828787691434_645743488253424111_o.jpg]

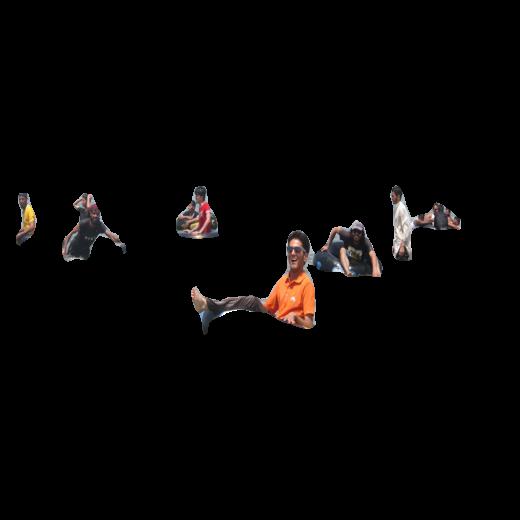

Supplement: Supplementary file 1 — Supplementary Information. [file 41598_2024_80657_MOESM1_ESM.zip › Dataset/High temperature human images/masked_10005869_623832464357733_9002293883507444564_o.jpg]

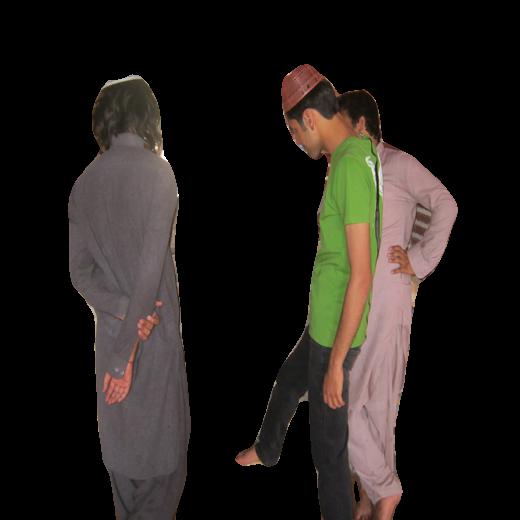

Supplement: Supplementary file 1 — Supplementary Information. [file 41598_2024_80657_MOESM1_ESM.zip › Dataset/High temperature human images/masked_10005901_623887477685565_3500127309762407181_o.jpg]

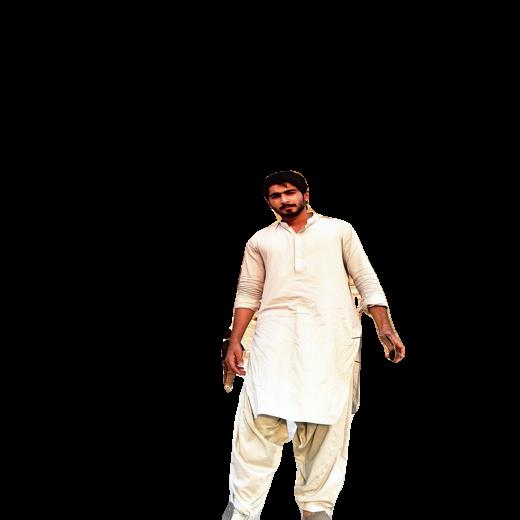

Supplement: Supplementary file 1 — Supplementary Information. [file 41598_2024_80657_MOESM1_ESM.zip › Dataset/High temperature human images/masked_10006004_979693885380102_8167018091657943619_o.jpg]

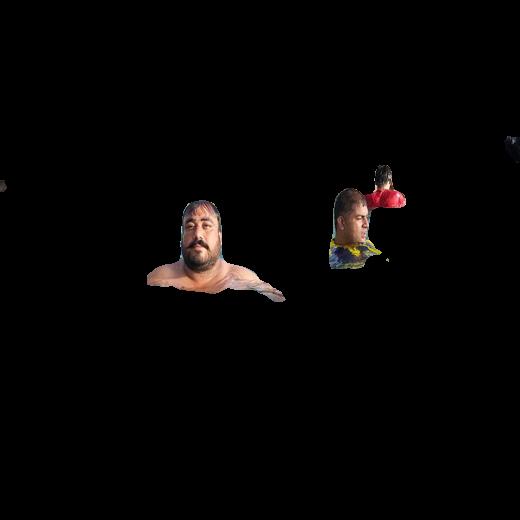

Supplement: Supplementary file 1 — Supplementary Information. [file 41598_2024_80657_MOESM1_ESM.zip › Dataset/High temperature human images/masked_10006511_762432517140758_6931091427826576723_n (1).jpg]

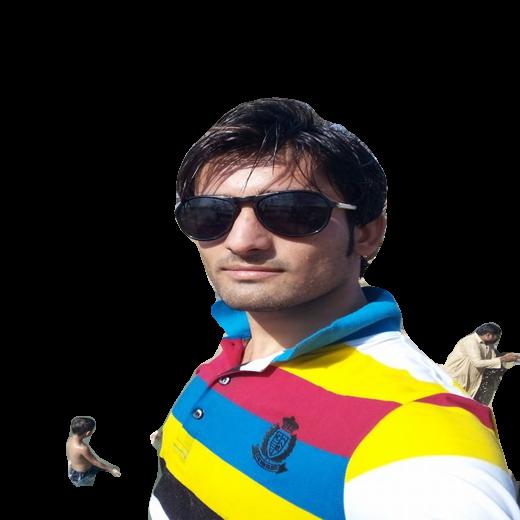

Supplement: Supplementary file 1 — Supplementary Information. [file 41598_2024_80657_MOESM1_ESM.zip › Dataset/High temperature human images/masked_10006618_690081054362753_1541123389799799373_n.jpg]

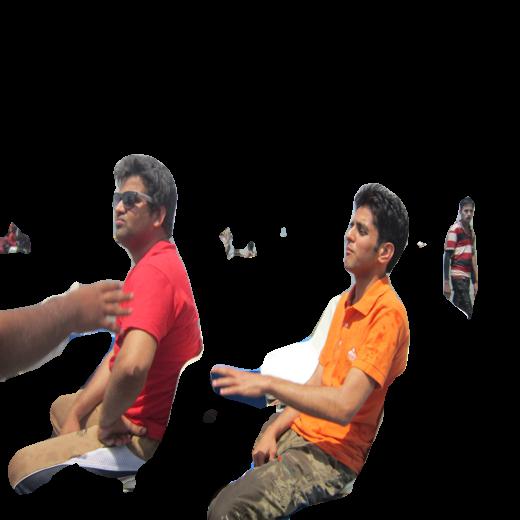

Supplement: Supplementary file 1 — Supplementary Information. [file 41598_2024_80657_MOESM1_ESM.zip › Dataset/High temperature human images/masked_10007307_623827967691516_6068452577270946015_o.jpg]

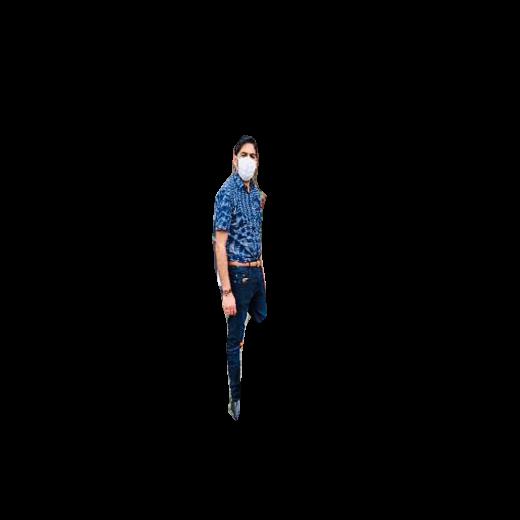

Supplement: Supplementary file 1 — Supplementary Information. [file 41598_2024_80657_MOESM1_ESM.zip › Dataset/High temperature human images/masked_100082562_2602638250010999_1727140477255286784_n.jpg]

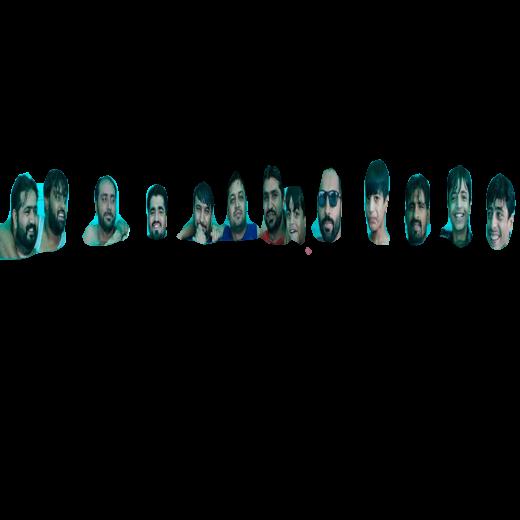

Supplement: Supplementary file 1 — Supplementary Information. [file 41598_2024_80657_MOESM1_ESM.zip › Dataset/High temperature human images/masked_100082562_2602638250010999_1727140477255286784_nl.jpg]

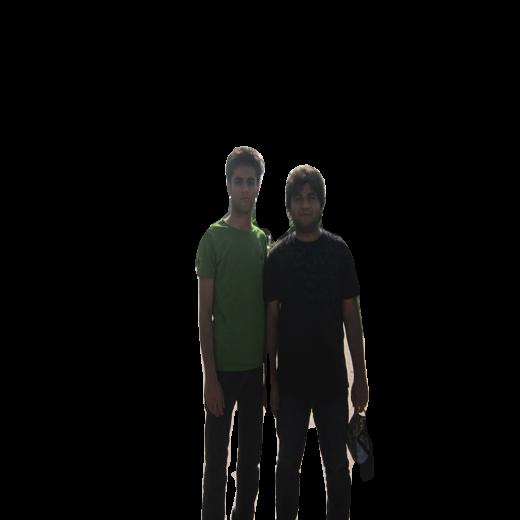

Supplement: Supplementary file 1 — Supplementary Information. [file 41598_2024_80657_MOESM1_ESM.zip › Dataset/High temperature human images/masked_10010235_623885851019061_3356238456430360482_o.jpg]

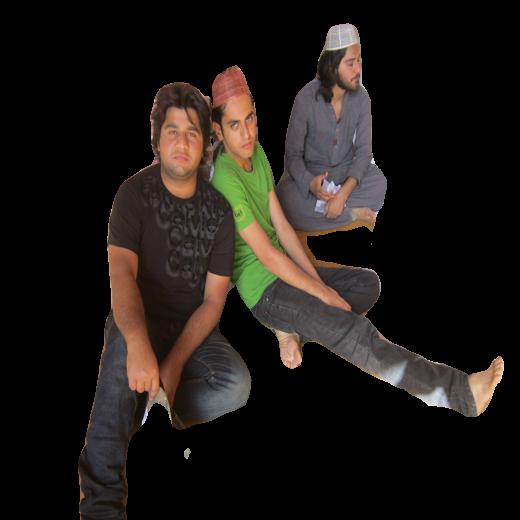

Supplement: Supplementary file 1 — Supplementary Information. [file 41598_2024_80657_MOESM1_ESM.zip › Dataset/High temperature human images/masked_10010385_623886377685675_3513638072604322266_o.jpg]

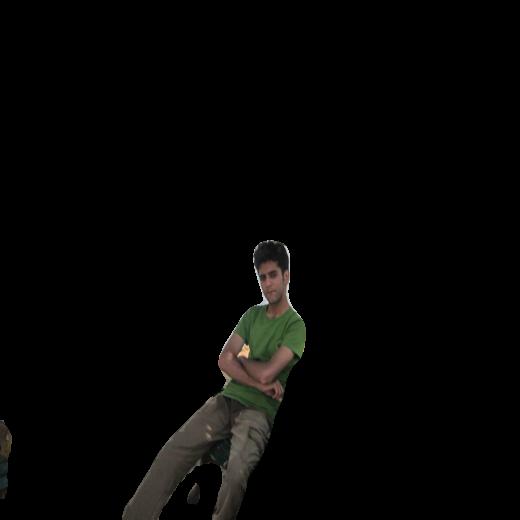

Supplement: Supplementary file 1 — Supplementary Information. [file 41598_2024_80657_MOESM1_ESM.zip › Dataset/High temperature human images/masked_10010445_623843894356590_3363015079675255806_o.jpg]

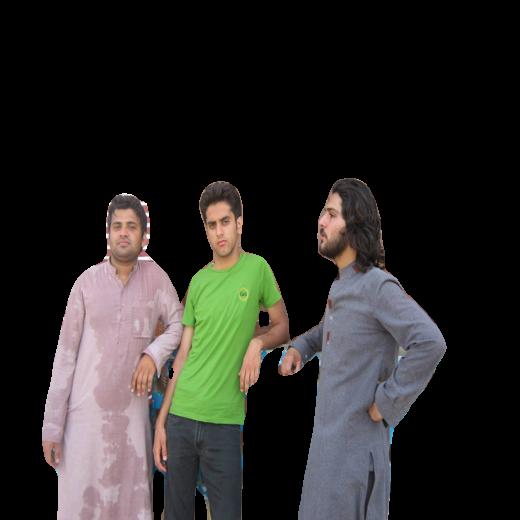

Supplement: Supplementary file 1 — Supplementary Information. [file 41598_2024_80657_MOESM1_ESM.zip › Dataset/High temperature human images/masked_10011698_623888164352163_7622628357941986044_o.jpg]

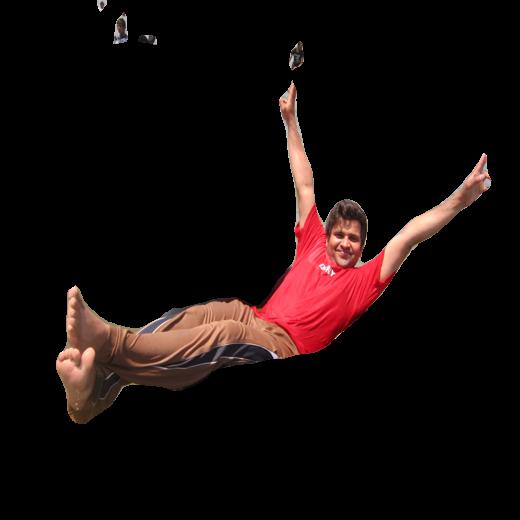

Supplement: Supplementary file 1 — Supplementary Information. [file 41598_2024_80657_MOESM1_ESM.zip › Dataset/High temperature human images/masked_10012784_623833711024275_2959840834550293260_o.jpg]

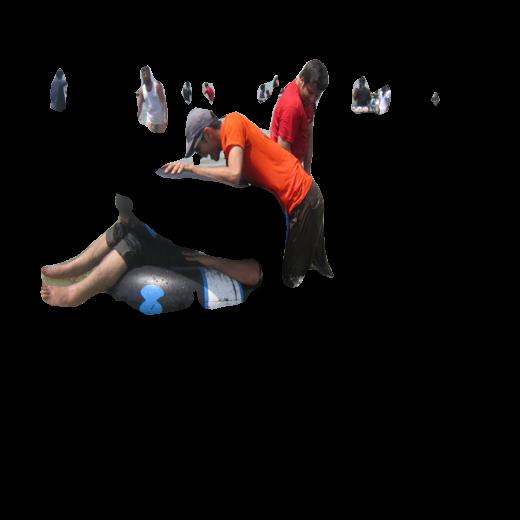

Supplement: Supplementary file 1 — Supplementary Information. [file 41598_2024_80657_MOESM1_ESM.zip › Dataset/High temperature human images/masked_10013391_623837444357235_7805182591701245354_o.jpg]

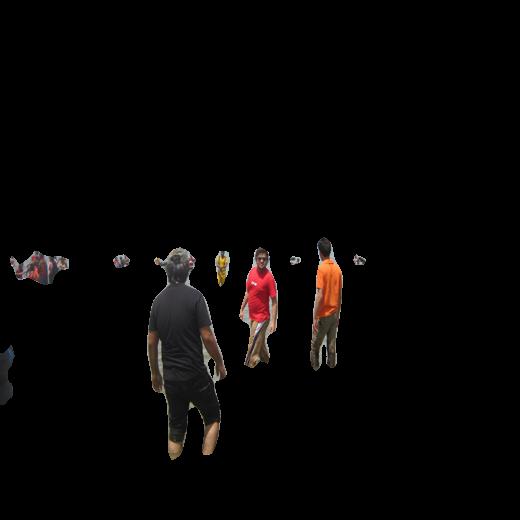

Supplement: Supplementary file 1 — Supplementary Information. [file 41598_2024_80657_MOESM1_ESM.zip › Dataset/High temperature human images/masked_10013395_623827627691550_4202116840604633357_o.jpg]

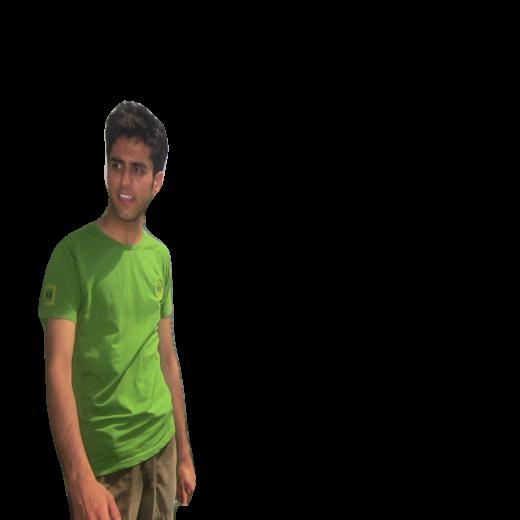

Supplement: Supplementary file 1 — Supplementary Information. [file 41598_2024_80657_MOESM1_ESM.zip › Dataset/High temperature human images/masked_10013426_623844714356508_1130619234089063259_o.jpg]

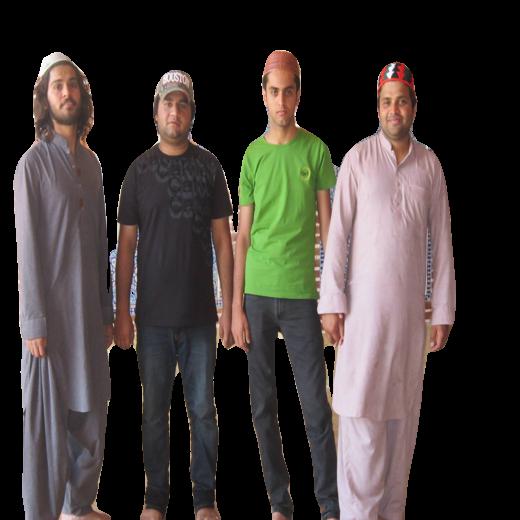

Supplement: Supplementary file 1 — Supplementary Information. [file 41598_2024_80657_MOESM1_ESM.zip › Dataset/High temperature human images/masked_10013446_623887074352272_5976893439546801494_o.jpg]

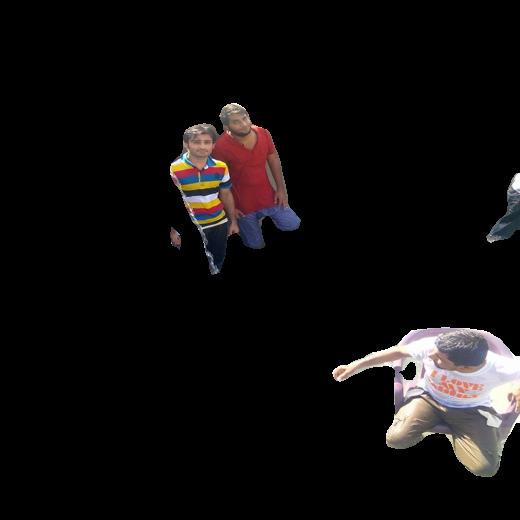

Supplement: Supplementary file 1 — Supplementary Information. [file 41598_2024_80657_MOESM1_ESM.zip › Dataset/High temperature human images/masked_10013884_690081907696001_5693707714752927658_n.jpg]

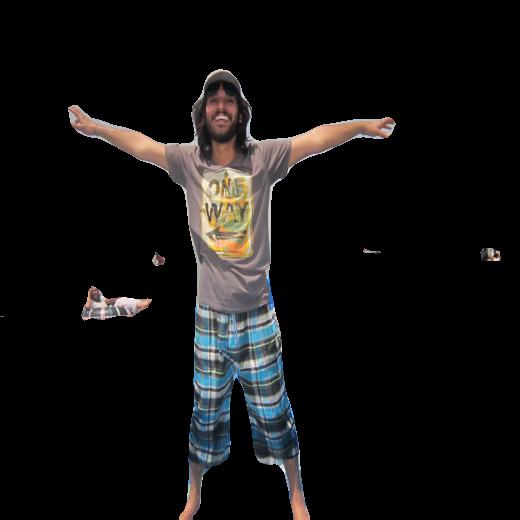

Supplement: Supplementary file 1 — Supplementary Information. [file 41598_2024_80657_MOESM1_ESM.zip › Dataset/High temperature human images/masked_10014011_623829851024661_8105959978530636224_o.jpg]

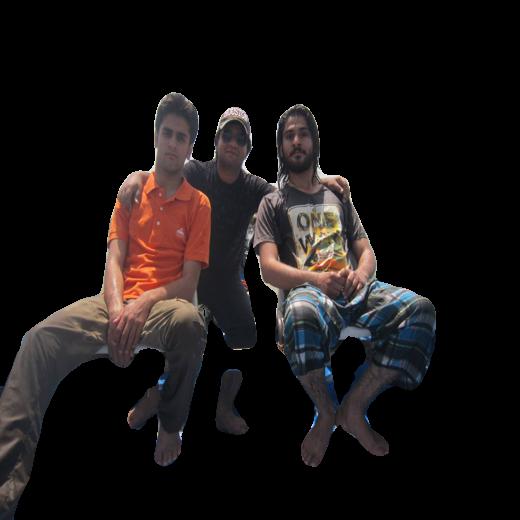

Supplement: Supplementary file 1 — Supplementary Information. [file 41598_2024_80657_MOESM1_ESM.zip › Dataset/High temperature human images/masked_10014050_623830277691285_6848474725124379882_o.jpg]

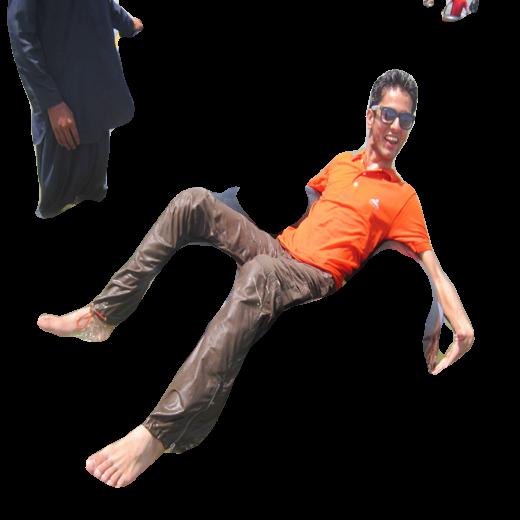

Supplement: Supplementary file 1 — Supplementary Information. [file 41598_2024_80657_MOESM1_ESM.zip › Dataset/High temperature human images/masked_10014056_623833961024250_5996208551976487852_o.jpg]

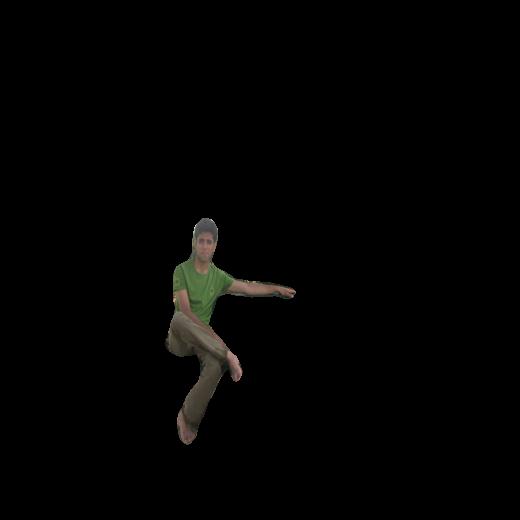

Supplement: Supplementary file 1 — Supplementary Information. [file 41598_2024_80657_MOESM1_ESM.zip › Dataset/High temperature human images/masked_10014065_623844977689815_2454334484050890512_o.jpg]

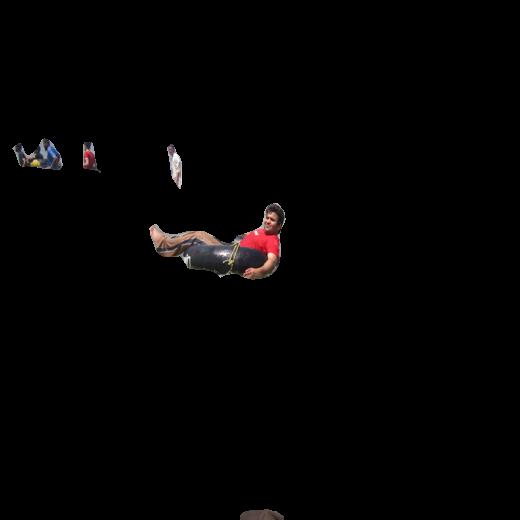

Supplement: Supplementary file 1 — Supplementary Information. [file 41598_2024_80657_MOESM1_ESM.zip › Dataset/High temperature human images/masked_10014226_623833734357606_7122160512047248180_o.jpg]

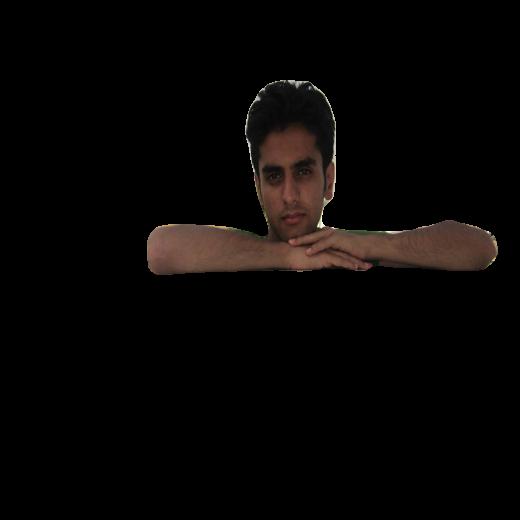

Supplement: Supplementary file 1 — Supplementary Information. [file 41598_2024_80657_MOESM1_ESM.zip › Dataset/High temperature human images/masked_10014316_623842601023386_8773722847975126254_o.jpg]

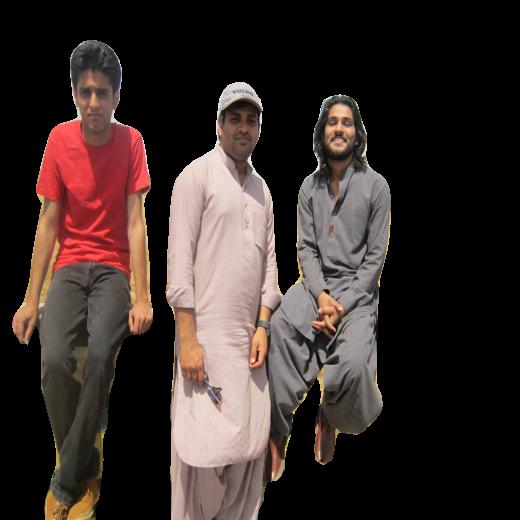

Supplement: Supplementary file 1 — Supplementary Information. [file 41598_2024_80657_MOESM1_ESM.zip › Dataset/High temperature human images/masked_10014718_623823821025264_7178499137109331751_o.jpg]

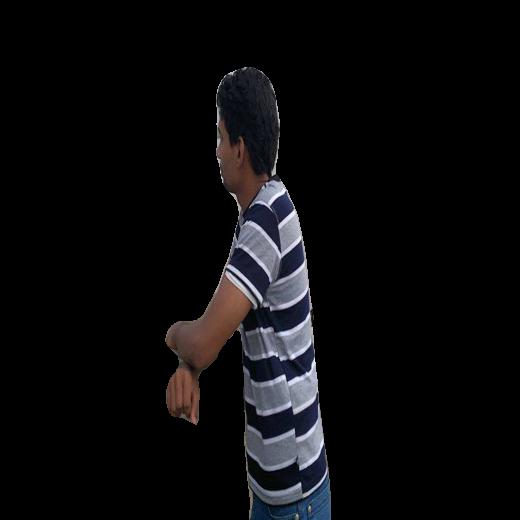

Supplement: Supplementary file 1 — Supplementary Information. [file 41598_2024_80657_MOESM1_ESM.zip › Dataset/High temperature human images/masked_1001622_406818122765768_1287387900_n.jpg]

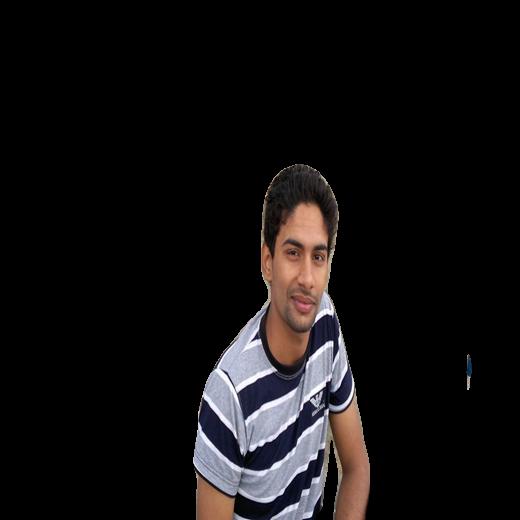

Supplement: Supplementary file 1 — Supplementary Information. [file 41598_2024_80657_MOESM1_ESM.zip › Dataset/High temperature human images/masked_1003652_406817116099202_34484182_n.jpg]

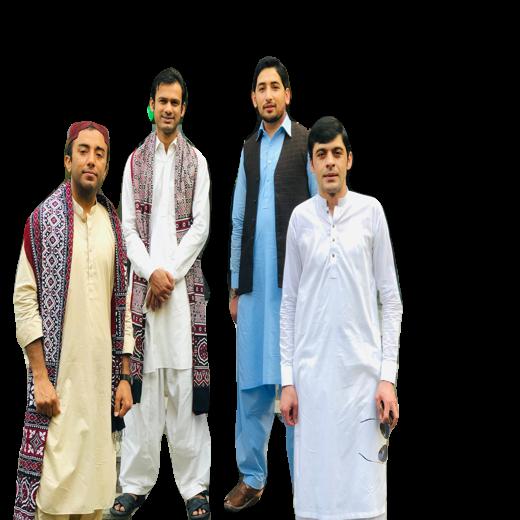

Supplement: Supplementary file 1 — Supplementary Information. [file 41598_2024_80657_MOESM1_ESM.zip › Dataset/High temperature human images/masked_100510262_2904379082980941_752474924479676416_n.jpg]

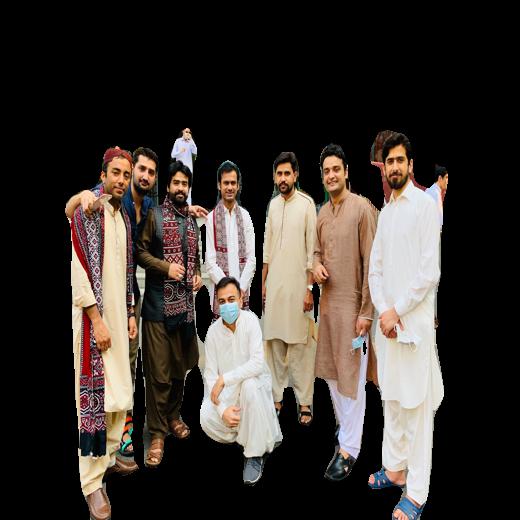

Supplement: Supplementary file 1 — Supplementary Information. [file 41598_2024_80657_MOESM1_ESM.zip › Dataset/High temperature human images/masked_100522746_2904378746314308_4797545441057046528_n.jpg]

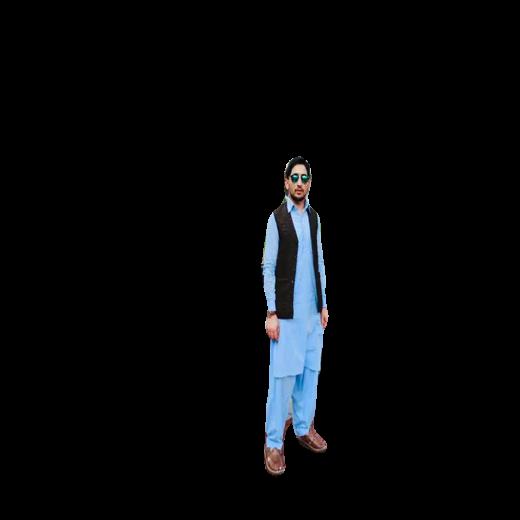

Supplement: Supplementary file 1 — Supplementary Information. [file 41598_2024_80657_MOESM1_ESM.zip › Dataset/High temperature human images/masked_100545688_2602638036677687_8399469267916947456_n.jpg]

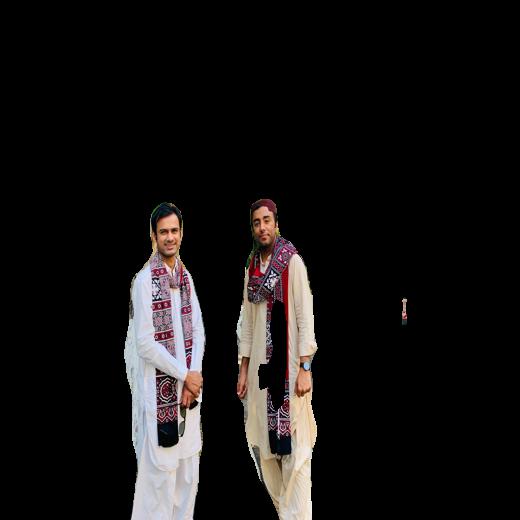

Supplement: Supplementary file 1 — Supplementary Information. [file 41598_2024_80657_MOESM1_ESM.zip › Dataset/High temperature human images/masked_100593721_2904387919646724_715200054368927744_n.jpg]

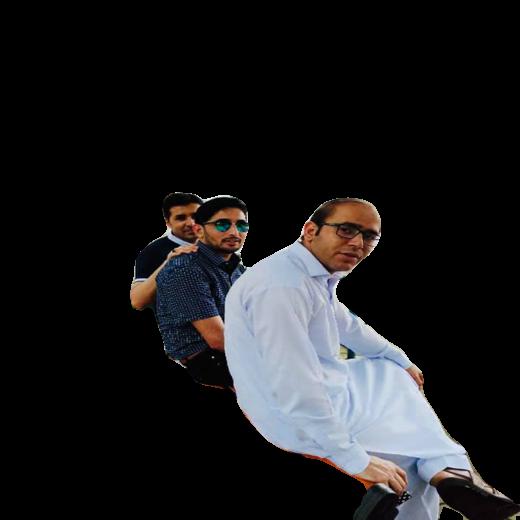

Supplement: Supplementary file 1 — Supplementary Information. [file 41598_2024_80657_MOESM1_ESM.zip › Dataset/High temperature human images/masked_100625572_2602637666677724_302687614027169792_n.jpg]

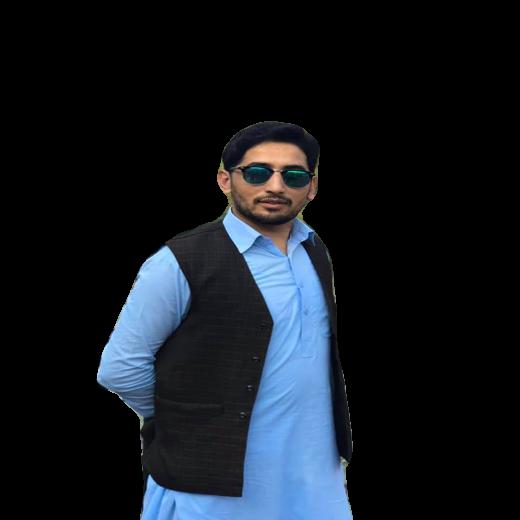

Supplement: Supplementary file 1 — Supplementary Information. [file 41598_2024_80657_MOESM1_ESM.zip › Dataset/High temperature human images/masked_100660218_2601042200170604_6622473265556226048_n.jpg]

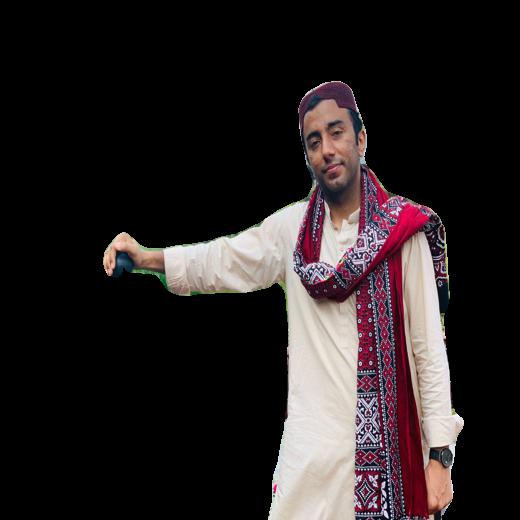

Supplement: Supplementary file 1 — Supplementary Information. [file 41598_2024_80657_MOESM1_ESM.zip › Dataset/High temperature human images/masked_100810890_2904385699646946_7710853407547850752_n.jpg]

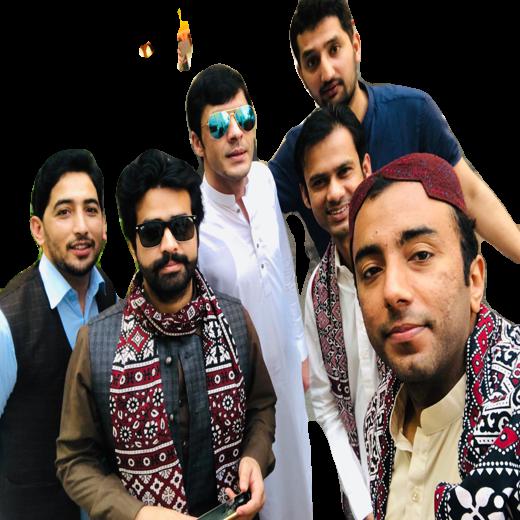

Supplement: Supplementary file 1 — Supplementary Information. [file 41598_2024_80657_MOESM1_ESM.zip › Dataset/High temperature human images/masked_100819707_2904386616313521_4682311919476932608_n.jpg]

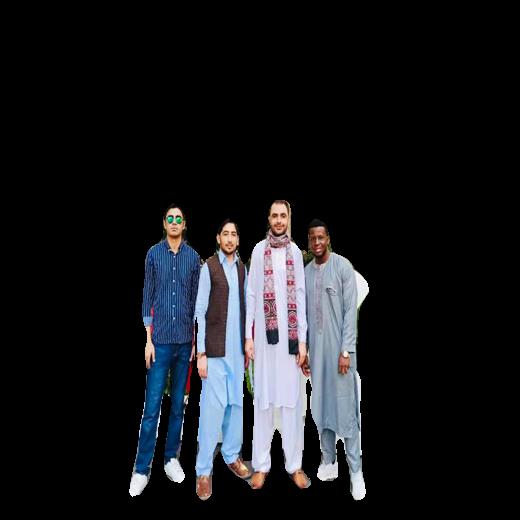

Supplement: Supplementary file 1 — Supplementary Information. [file 41598_2024_80657_MOESM1_ESM.zip › Dataset/High temperature human images/masked_100823443_2602637920011032_7092576054786654208_n.jpg]

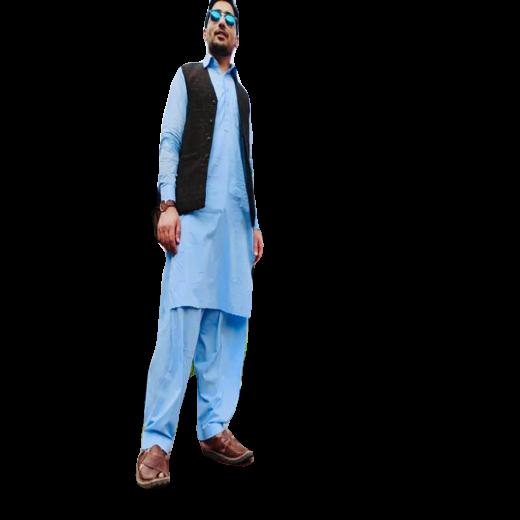

Supplement: Supplementary file 1 — Supplementary Information. [file 41598_2024_80657_MOESM1_ESM.zip › Dataset/High temperature human images/masked_100873419_2602637926677698_4418052301265043456_n.jpg]

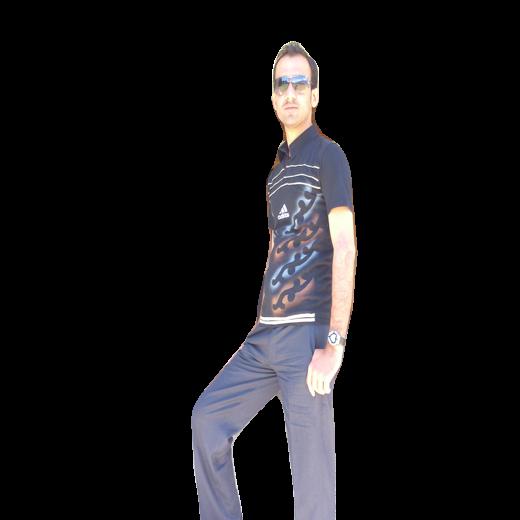

Supplement: Supplementary file 1 — Supplementary Information. [file 41598_2024_80657_MOESM1_ESM.zip › Dataset/High temperature human images/masked_1008930_207431239409540_418175511_o.jpg]

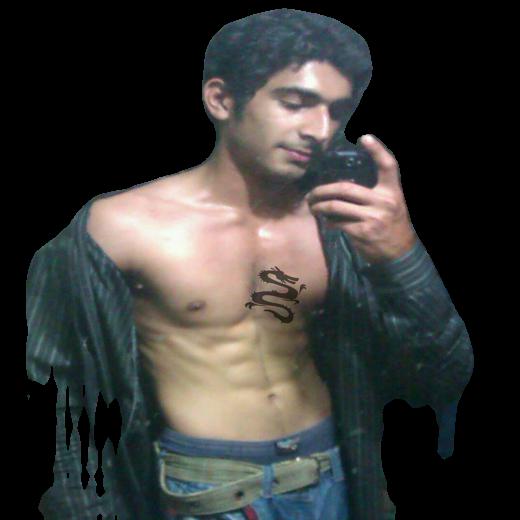

Supplement: Supplementary file 1 — Supplementary Information. [file 41598_2024_80657_MOESM1_ESM.zip › Dataset/High temperature human images/masked_1009593_678156248867202_2039947900_o.jpg]

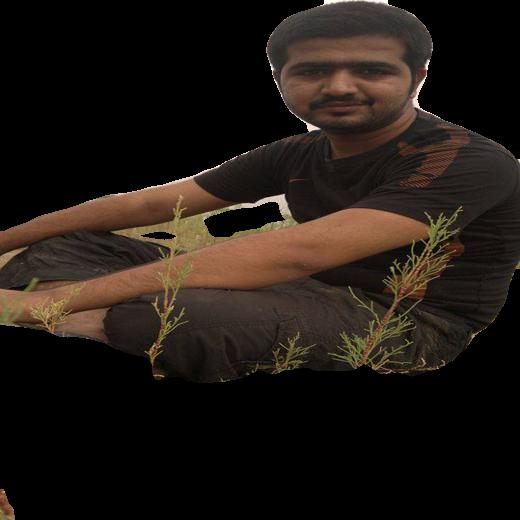

Supplement: Supplementary file 1 — Supplementary Information. [file 41598_2024_80657_MOESM1_ESM.zip › Dataset/High temperature human images/masked_1010163_406818012765779_1762765302_n.jpg]

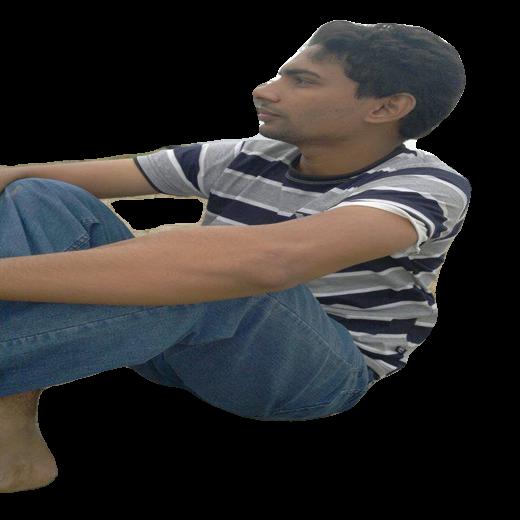

Supplement: Supplementary file 1 — Supplementary Information. [file 41598_2024_80657_MOESM1_ESM.zip › Dataset/High temperature human images/masked_1011127_406818226099091_1604369605_n.jpg]

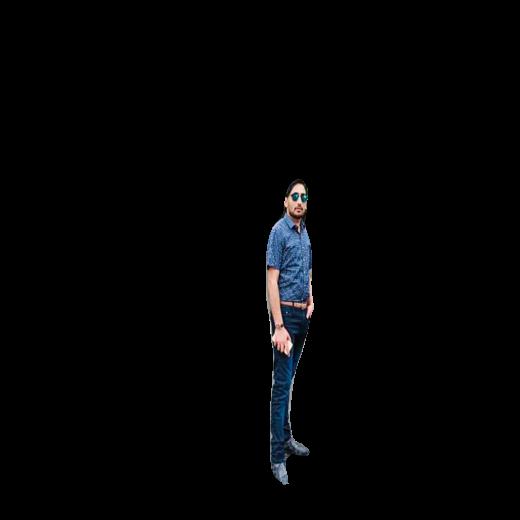

Supplement: Supplementary file 1 — Supplementary Information. [file 41598_2024_80657_MOESM1_ESM.zip › Dataset/High temperature human images/masked_101154113_2602637650011059_8064399735076683776_n.jpg]

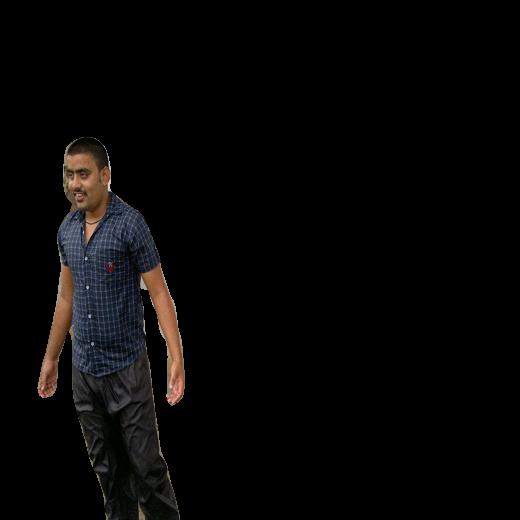

Supplement: Supplementary file 1 — Supplementary Information. [file 41598_2024_80657_MOESM1_ESM.zip › Dataset/High temperature human images/masked_1011756_406816019432645_650880684_n.jpg]

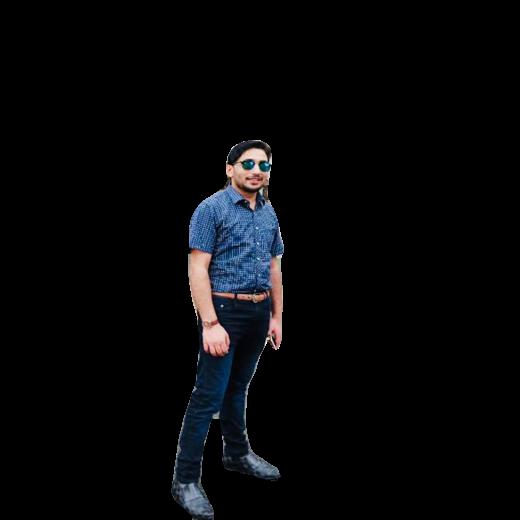

Supplement: Supplementary file 1 — Supplementary Information. [file 41598_2024_80657_MOESM1_ESM.zip › Dataset/High temperature human images/masked_101309867_2602638153344342_3175267101434707968_n.jpg]

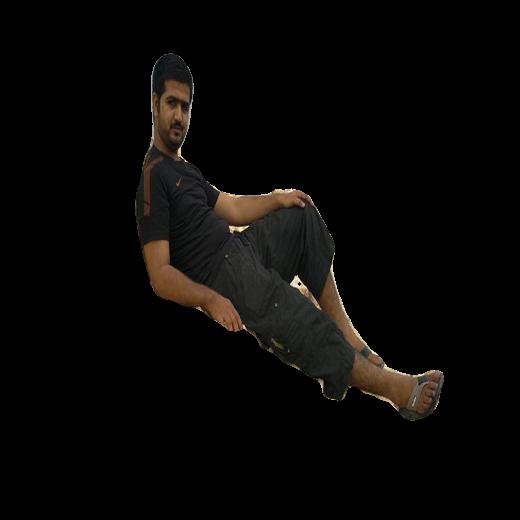

Supplement: Supplementary file 1 — Supplementary Information. [file 41598_2024_80657_MOESM1_ESM.zip › Dataset/High temperature human images/masked_1013691_406817432765837_1482583738_n.jpg]

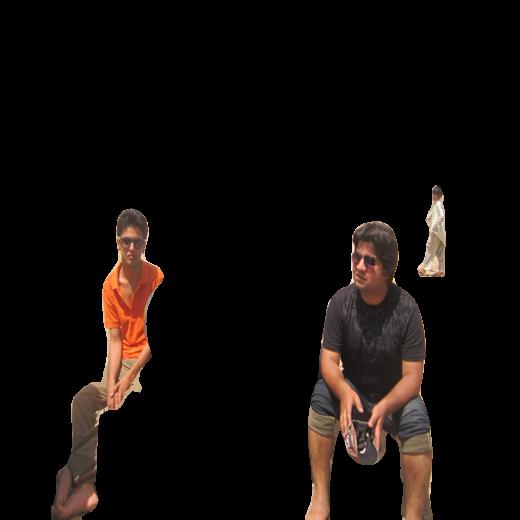

Supplement: Supplementary file 1 — Supplementary Information. [file 41598_2024_80657_MOESM1_ESM.zip › Dataset/High temperature human images/masked_1015025_623826507691662_3173482352320132535_o.jpg]

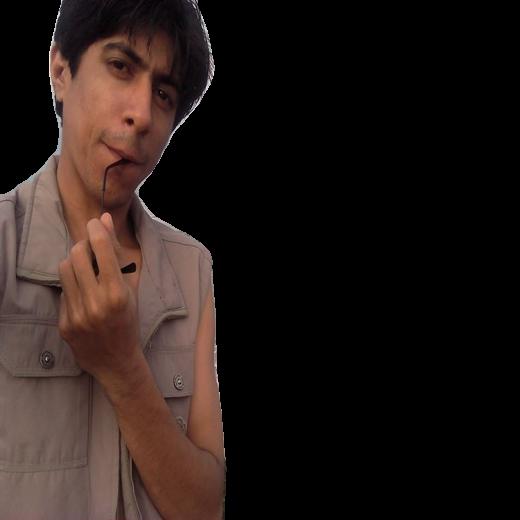

Supplement: Supplementary file 1 — Supplementary Information. [file 41598_2024_80657_MOESM1_ESM.zip › Dataset/High temperature human images/masked_10151907_746040902136427_4826129963275712336_n.jpg]

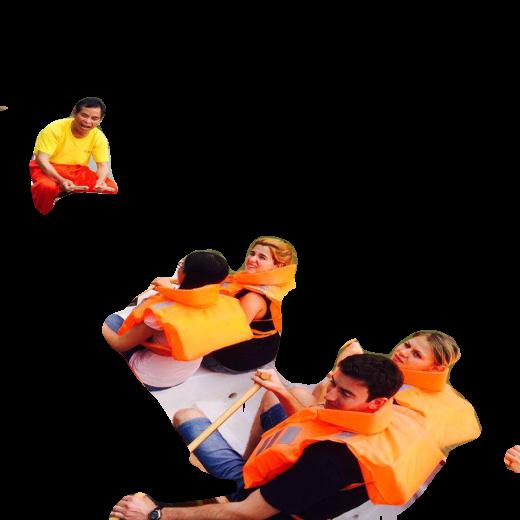

Supplement: Supplementary file 1 — Supplementary Information. [file 41598_2024_80657_MOESM1_ESM.zip › Dataset/High temperature human images/masked_10154348_943731209012731_3209738637787927237_n.jpg]

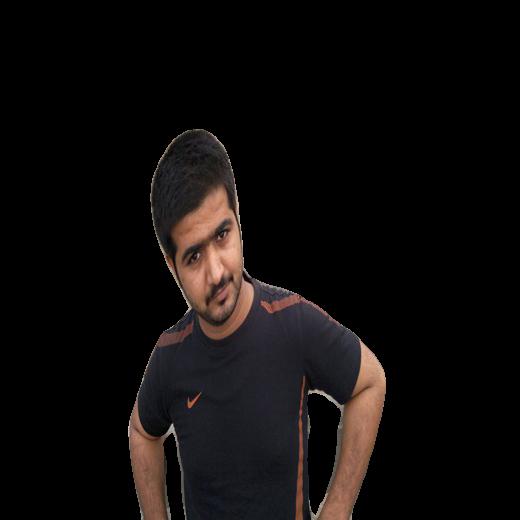

Supplement: Supplementary file 1 — Supplementary Information. [file 41598_2024_80657_MOESM1_ESM.zip › Dataset/High temperature human images/masked_1017051_406818302765750_281123327_n.jpg]

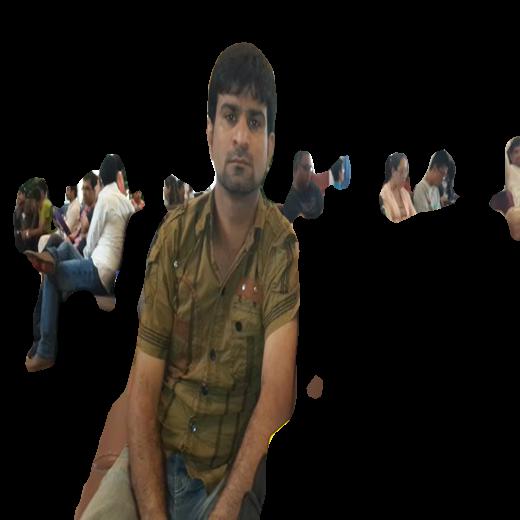

Supplement: Supplementary file 1 — Supplementary Information. [file 41598_2024_80657_MOESM1_ESM.zip › Dataset/High temperature human images/masked_10170714_788795104506343_2263129680471617376_n.jpg]

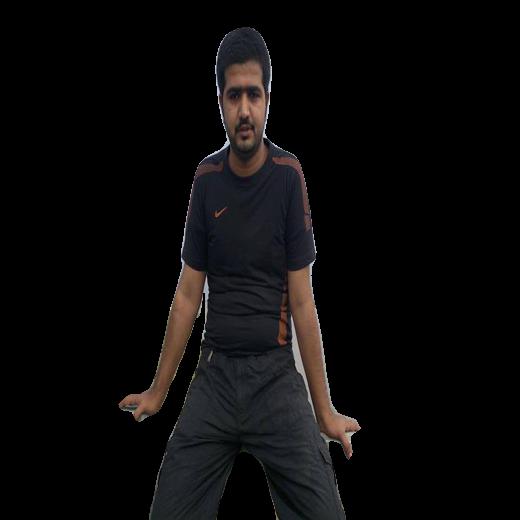

Supplement: Supplementary file 1 — Supplementary Information. [file 41598_2024_80657_MOESM1_ESM.zip › Dataset/High temperature human images/masked_1017089_406818399432407_1050918982_n.jpg]

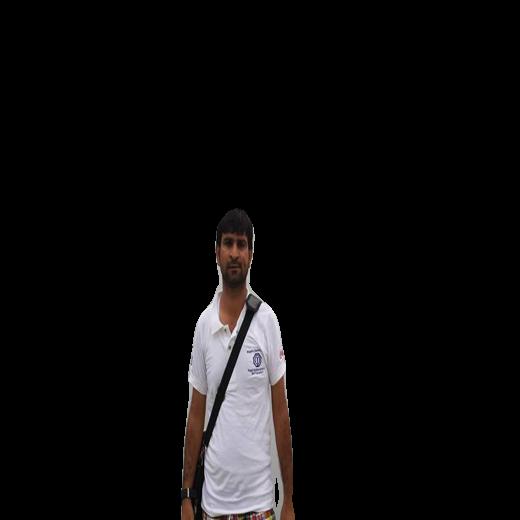

Supplement: Supplementary file 1 — Supplementary Information. [file 41598_2024_80657_MOESM1_ESM.zip › Dataset/High temperature human images/masked_10171629_789750111077509_6549257835974940836_n.jpg]

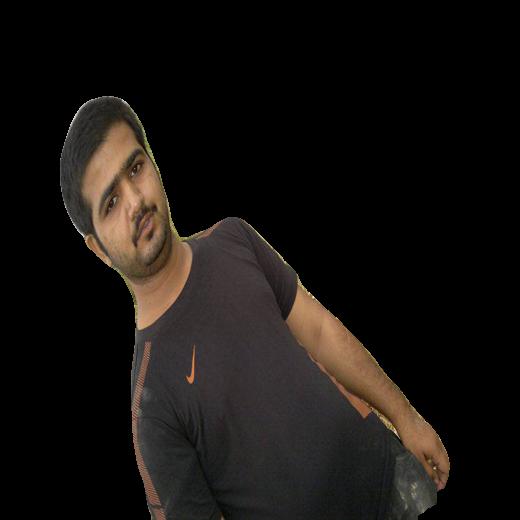

Supplement: Supplementary file 1 — Supplementary Information. [file 41598_2024_80657_MOESM1_ESM.zip › Dataset/High temperature human images/masked_1017445_406818589432388_1806962805_n.jpg]

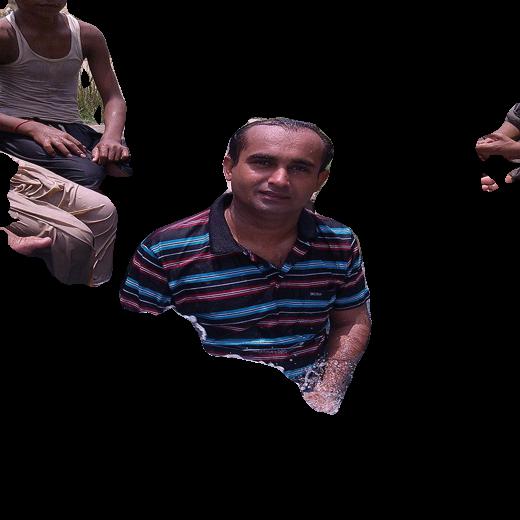

Supplement: Supplementary file 1 — Supplementary Information. [file 41598_2024_80657_MOESM1_ESM.zip › Dataset/High temperature human images/masked_1017460_752560364782664_6704727480764644402_n.jpg]

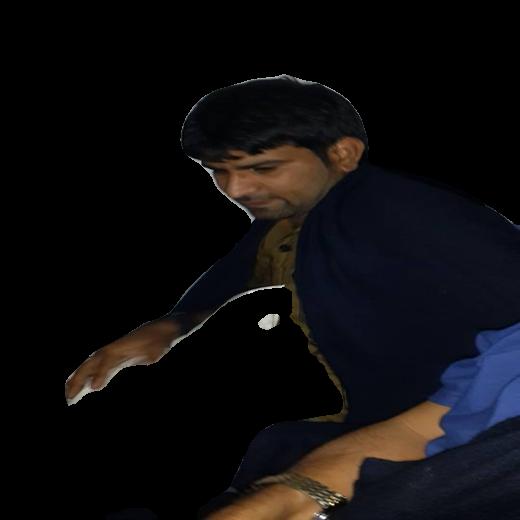

Supplement: Supplementary file 1 — Supplementary Information. [file 41598_2024_80657_MOESM1_ESM.zip › Dataset/High temperature human images/masked_10178061_788794991173021_5714769862531273409_n.jpg]

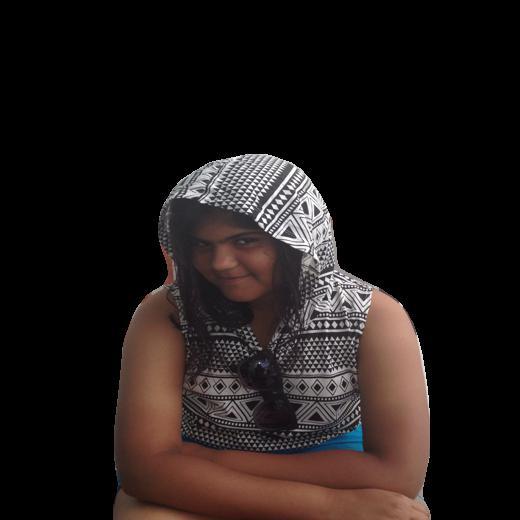

Supplement: Supplementary file 1 — Supplementary Information. [file 41598_2024_80657_MOESM1_ESM.zip › Dataset/High temperature human images/masked_1020173_394415330663405_661952371_o.jpg]

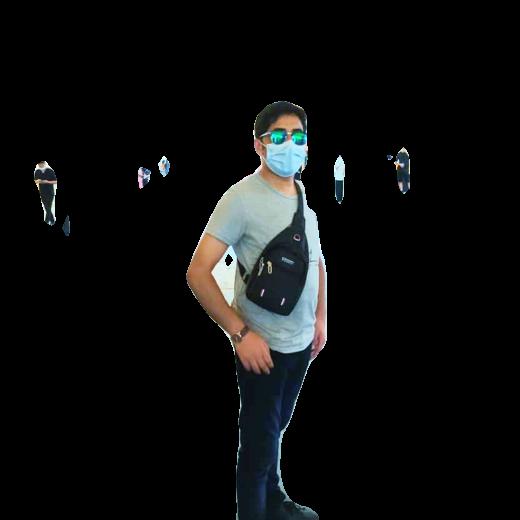

Supplement: Supplementary file 1 — Supplementary Information. [file 41598_2024_80657_MOESM1_ESM.zip › Dataset/High temperature human images/masked_102279744_2613936785547812_6964114596180025446_n.jpg]

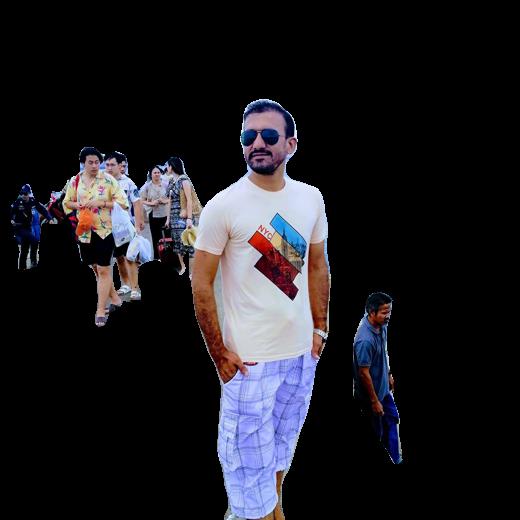

Supplement: Supplementary file 1 — Supplementary Information. [file 41598_2024_80657_MOESM1_ESM.zip › Dataset/High temperature human images/masked_102416226_1667676990051617_7743861616851452138_n.jpg]

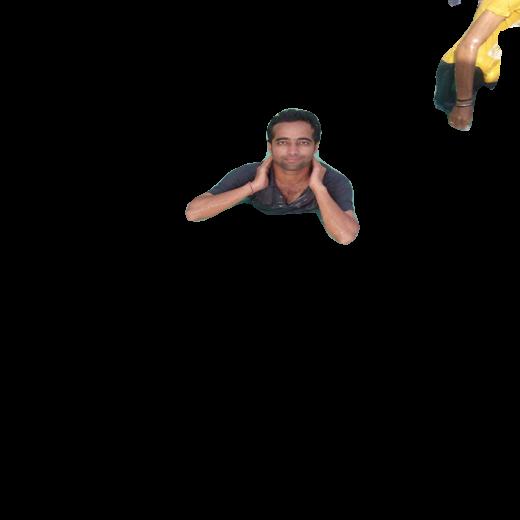

Supplement: Supplementary file 1 — Supplementary Information. [file 41598_2024_80657_MOESM1_ESM.zip › Dataset/High temperature human images/masked_10245280_620321368054925_2063026917983014729_n.jpg]

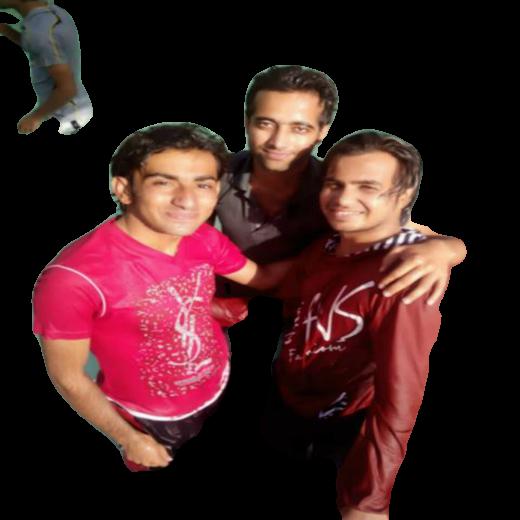

Supplement: Supplementary file 1 — Supplementary Information. [file 41598_2024_80657_MOESM1_ESM.zip › Dataset/High temperature human images/masked_10247288_620321961388199_1708835481993710462_n.jpg]

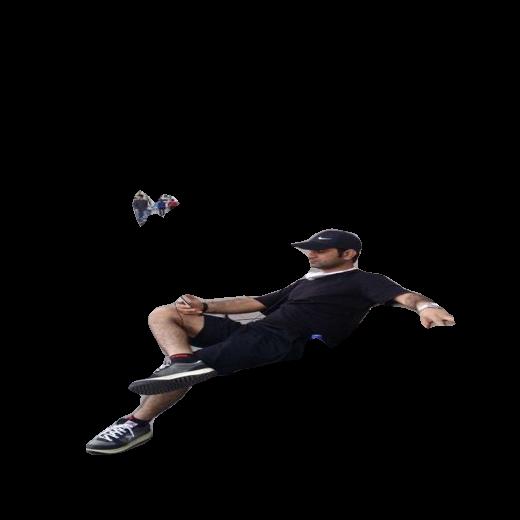

Supplement: Supplementary file 1 — Supplementary Information. [file 41598_2024_80657_MOESM1_ESM.zip › Dataset/High temperature human images/masked_10250158_911250862260766_1099635985185693357_n.jpg]

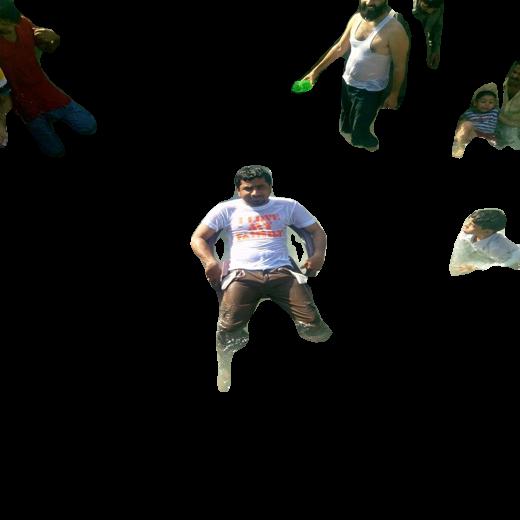

Supplement: Supplementary file 1 — Supplementary Information. [file 41598_2024_80657_MOESM1_ESM.zip › Dataset/High temperature human images/masked_10256495_690081984362660_4322529257030373812_n.jpg]

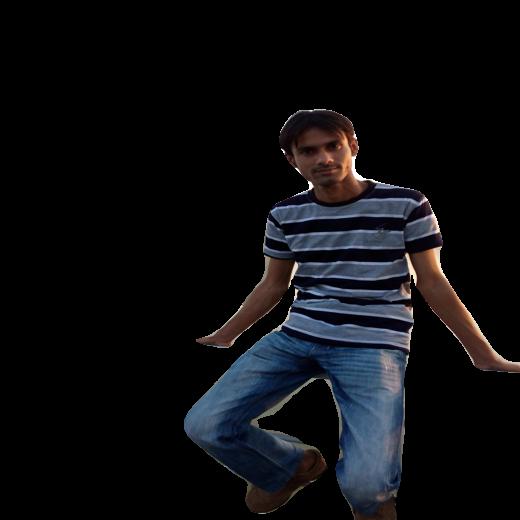

Supplement: Supplementary file 1 — Supplementary Information. [file 41598_2024_80657_MOESM1_ESM.zip › Dataset/High temperature human images/masked_10257103_910082035686177_1444357150399550008_o.jpg]

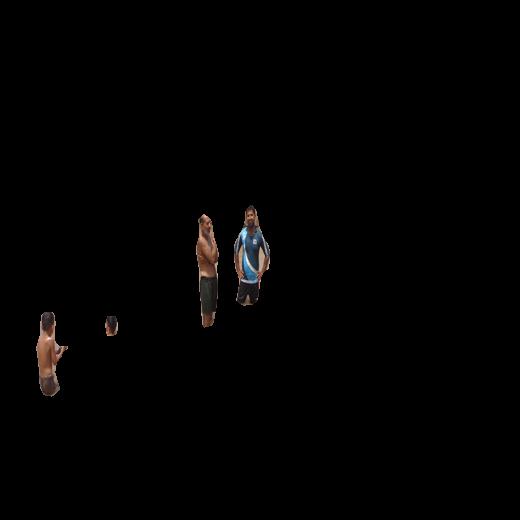

Supplement: Supplementary file 1 — Supplementary Information. [file 41598_2024_80657_MOESM1_ESM.zip › Dataset/High temperature human images/masked_10258261_725950757447979_2843408038791484514_o.jpg]

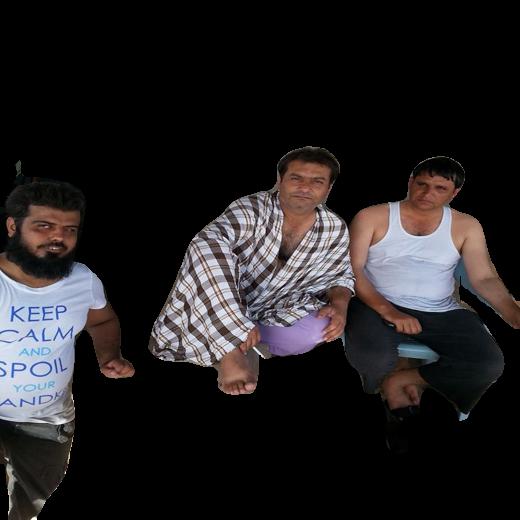

Supplement: Supplementary file 1 — Supplementary Information. [file 41598_2024_80657_MOESM1_ESM.zip › Dataset/High temperature human images/masked_10269424_762433937140616_313265303918599468_n.jpg]

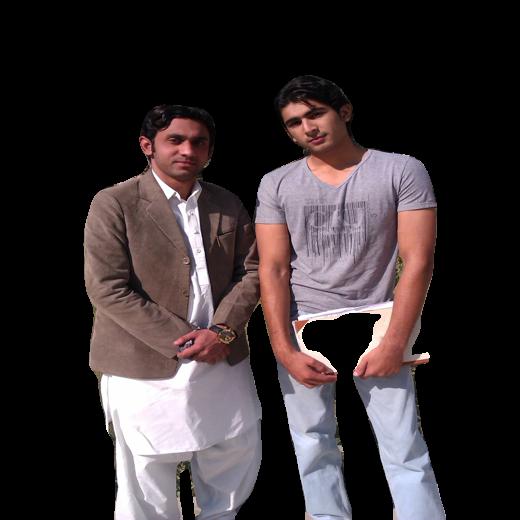

Supplement: Supplementary file 1 — Supplementary Information. [file 41598_2024_80657_MOESM1_ESM.zip › Dataset/High temperature human images/masked_10272749_883450098337815_4207777630273164196_o.jpg]

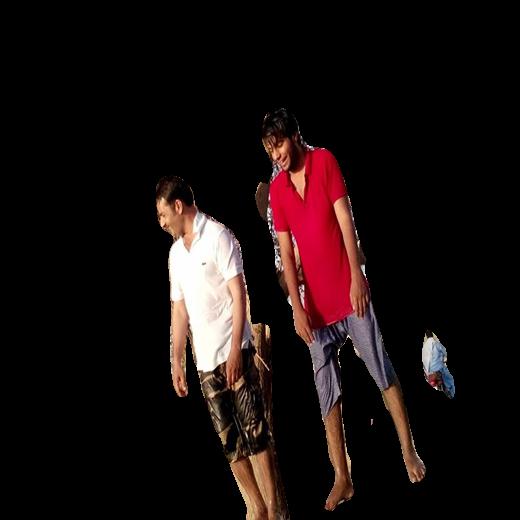

Supplement: Supplementary file 1 — Supplementary Information. [file 41598_2024_80657_MOESM1_ESM.zip › Dataset/High temperature human images/masked_10273605_690080691029456_6963145719584189933_n.jpg]

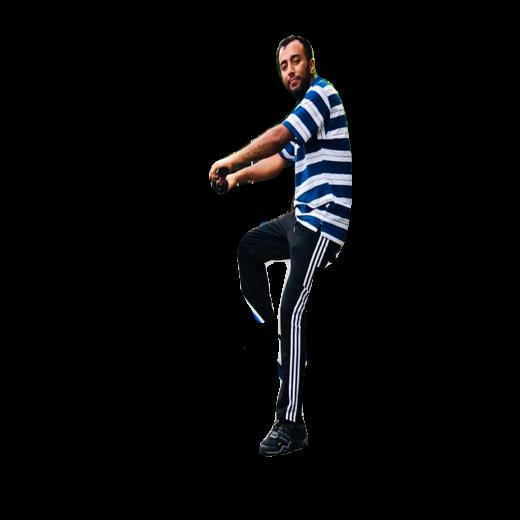

Supplement: Supplementary file 1 — Supplementary Information. [file 41598_2024_80657_MOESM1_ESM.zip › Dataset/High temperature human images/masked_102739488_2949562211795961_4629395664589593161_n.jpg]

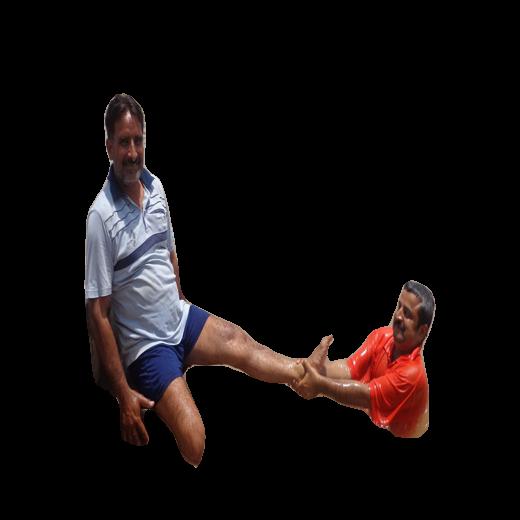

Supplement: Supplementary file 1 — Supplementary Information. [file 41598_2024_80657_MOESM1_ESM.zip › Dataset/High temperature human images/masked_10285726_725950470781341_6354019485987597057_o.jpg]

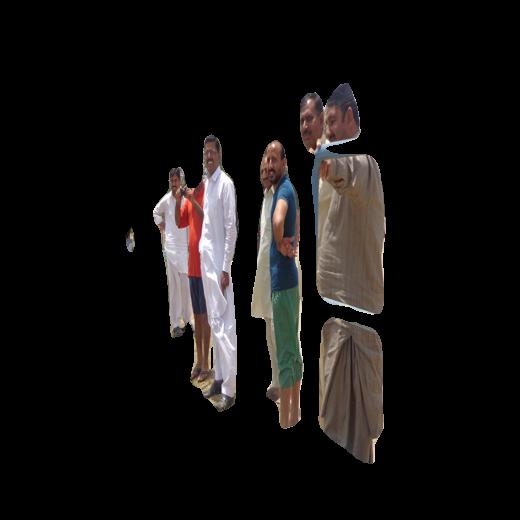

Supplement: Supplementary file 1 — Supplementary Information. [file 41598_2024_80657_MOESM1_ESM.zip › Dataset/High temperature human images/masked_10285784_725950434114678_7043297762068818730_o.jpg]

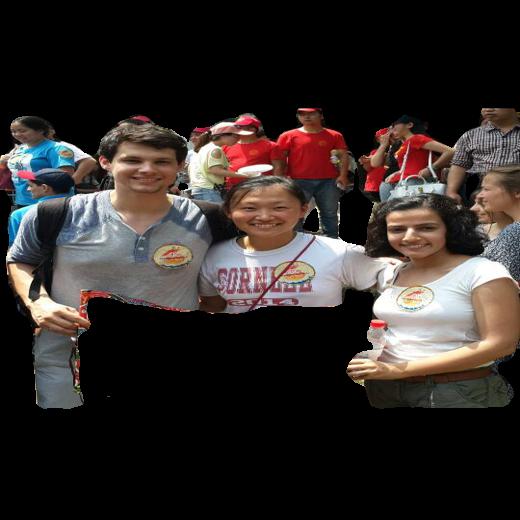

Supplement: Supplementary file 1 — Supplementary Information. [file 41598_2024_80657_MOESM1_ESM.zip › Dataset/High temperature human images/masked_10295701_944523102266875_3444691887194545873_n.jpg]

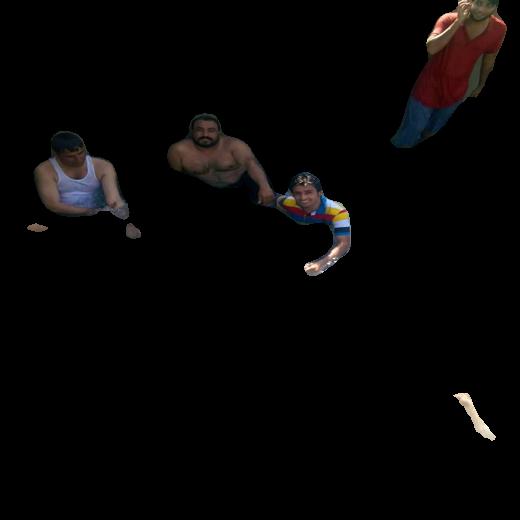

Supplement: Supplementary file 1 — Supplementary Information. [file 41598_2024_80657_MOESM1_ESM.zip › Dataset/High temperature human images/masked_10297774_690082534362605_409076392702236874_n.jpg]

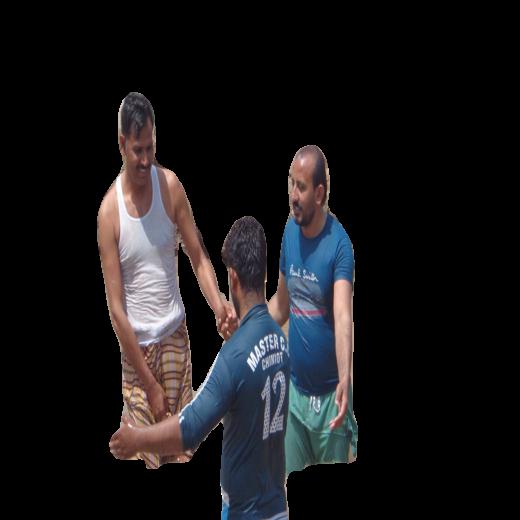

Supplement: Supplementary file 1 — Supplementary Information. [file 41598_2024_80657_MOESM1_ESM.zip › Dataset/High temperature human images/masked_10298178_725951080781280_3759624075001217450_o.jpg]

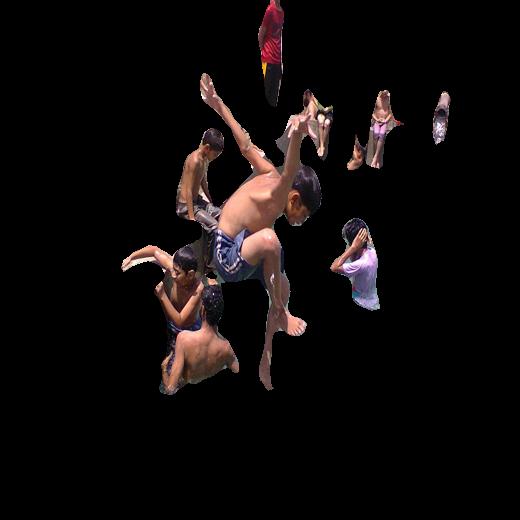

Supplement: Supplementary file 1 — Supplementary Information. [file 41598_2024_80657_MOESM1_ESM.zip › Dataset/High temperature human images/masked_10300867_811970392160033_1857134518679542302_n.jpg]

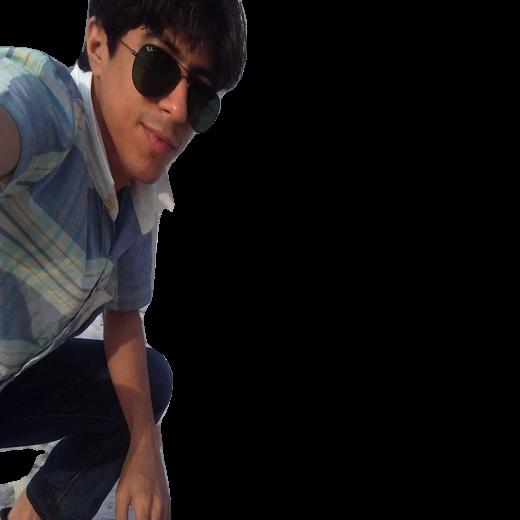

Supplement: Supplementary file 1 — Supplementary Information. [file 41598_2024_80657_MOESM1_ESM.zip › Dataset/High temperature human images/masked_10300875_746040582136459_3192669784483034290_n.jpg]

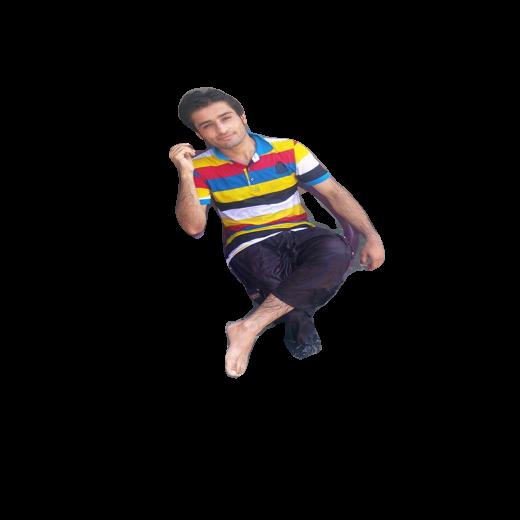

Supplement: Supplementary file 1 — Supplementary Information. [file 41598_2024_80657_MOESM1_ESM.zip › Dataset/High temperature human images/masked_10301065_690081557696036_8490133017985319221_n.jpg]

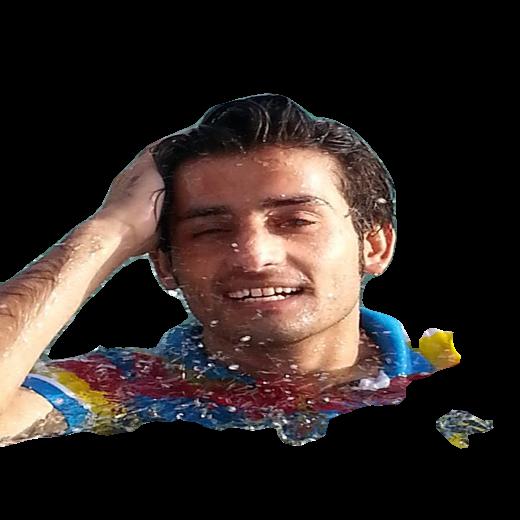

Supplement: Supplementary file 1 — Supplementary Information. [file 41598_2024_80657_MOESM1_ESM.zip › Dataset/High temperature human images/masked_10303171_762433480473995_3204837260505202371_n.jpg]

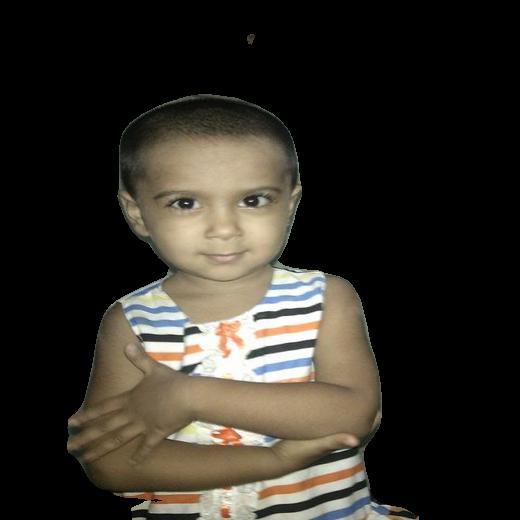

Supplement: Supplementary file 1 — Supplementary Information. [file 41598_2024_80657_MOESM1_ESM.zip › Dataset/High temperature human images/masked_10303794_739097676128933_5644884855013611489_n.jpg]

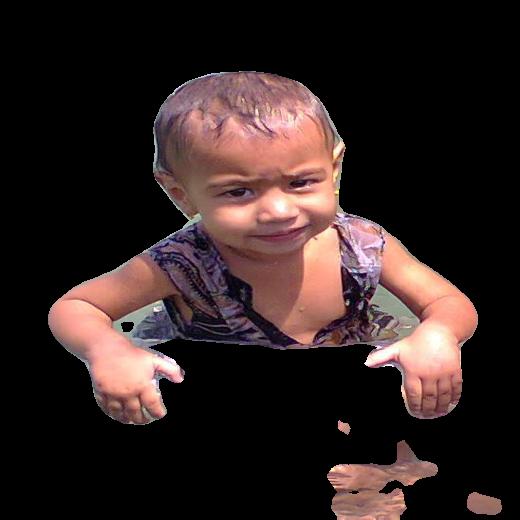

Supplement: Supplementary file 1 — Supplementary Information. [file 41598_2024_80657_MOESM1_ESM.zip › Dataset/High temperature human images/masked_10306645_811970472160025_9089800844693924507_n.jpg]

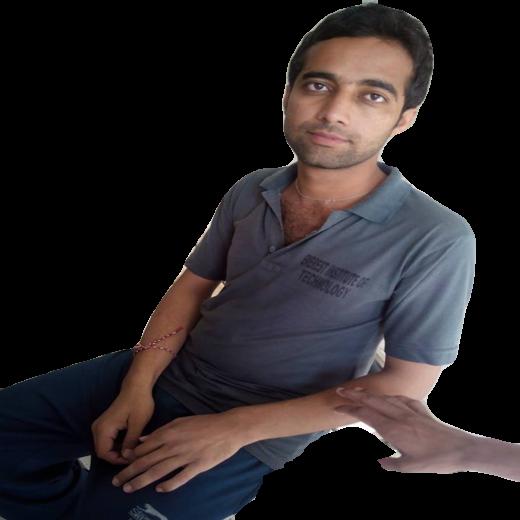

Supplement: Supplementary file 1 — Supplementary Information. [file 41598_2024_80657_MOESM1_ESM.zip › Dataset/High temperature human images/masked_10306772_620319931388402_7722865588427419805_n.jpg]

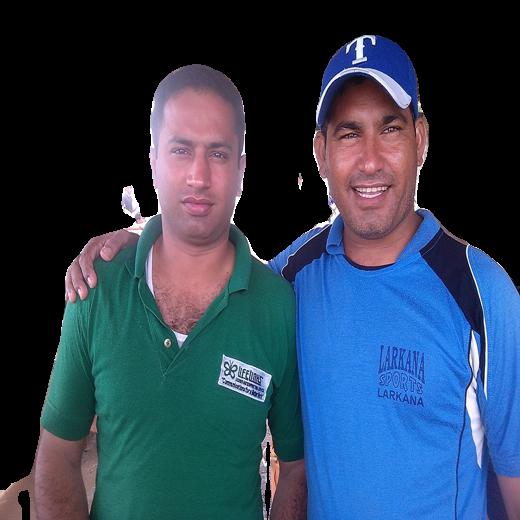

Supplement: Supplementary file 1 — Supplementary Information. [file 41598_2024_80657_MOESM1_ESM.zip › Dataset/High temperature human images/masked_10310643_751603071545060_892751763805731293_n.jpg]

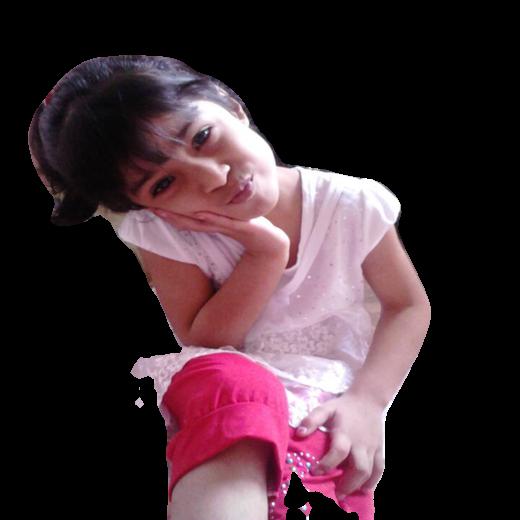

Supplement: Supplementary file 1 — Supplementary Information. [file 41598_2024_80657_MOESM1_ESM.zip › Dataset/High temperature human images/masked_10314494_896513223734530_769010822094591665_n.jpg]

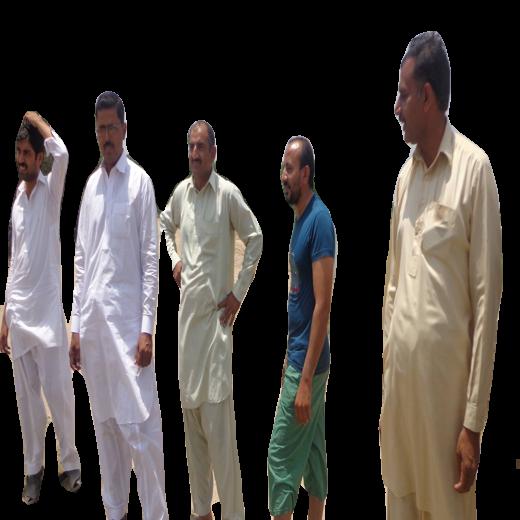

Supplement: Supplementary file 1 — Supplementary Information. [file 41598_2024_80657_MOESM1_ESM.zip › Dataset/High temperature human images/masked_10321739_725950344114687_648897345038378917_o.jpg]

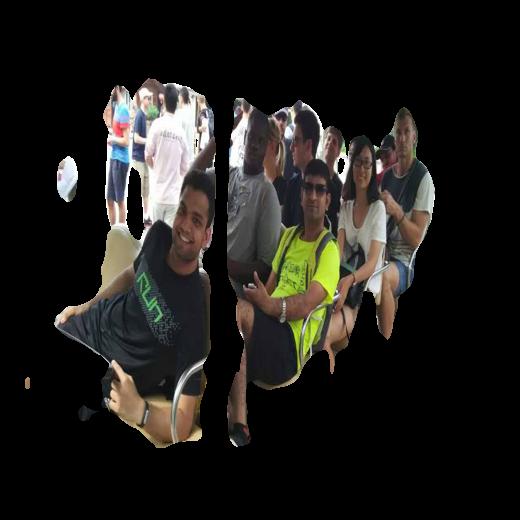

Supplement: Supplementary file 1 — Supplementary Information. [file 41598_2024_80657_MOESM1_ESM.zip › Dataset/High temperature human images/masked_10329663_943731039012748_7391760670875413523_o.jpg]

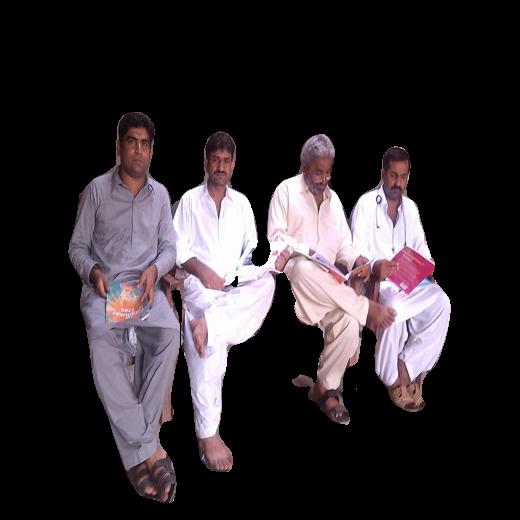

Supplement: Supplementary file 1 — Supplementary Information. [file 41598_2024_80657_MOESM1_ESM.zip › Dataset/High temperature human images/masked_10330500_750225368349497_1324266183068687321_n.jpg]

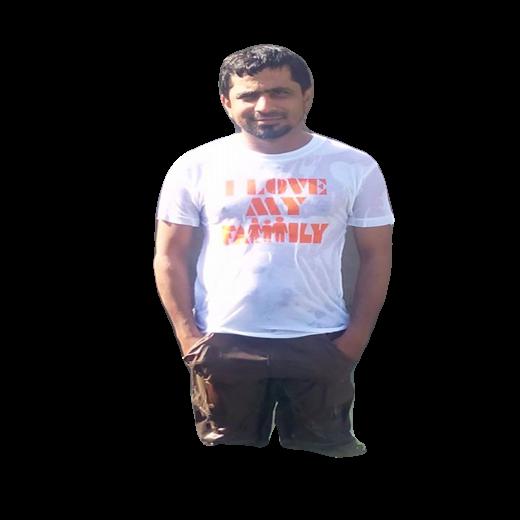

Supplement: Supplementary file 1 — Supplementary Information. [file 41598_2024_80657_MOESM1_ESM.zip › Dataset/High temperature human images/masked_10336709_690081371029388_4425128746821897015_n.jpg]

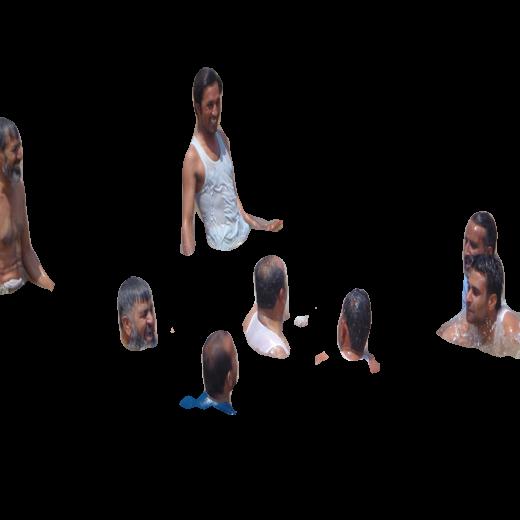

Supplement: Supplementary file 1 — Supplementary Information. [file 41598_2024_80657_MOESM1_ESM.zip › Dataset/High temperature human images/masked_10338615_725951130781275_5057040441371276401_o.jpg]

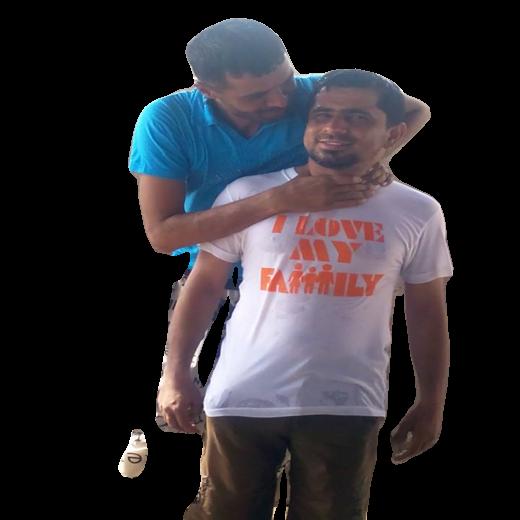

Supplement: Supplementary file 1 — Supplementary Information. [file 41598_2024_80657_MOESM1_ESM.zip › Dataset/High temperature human images/masked_10340151_690080927696099_1235928534411310392_n.jpg]

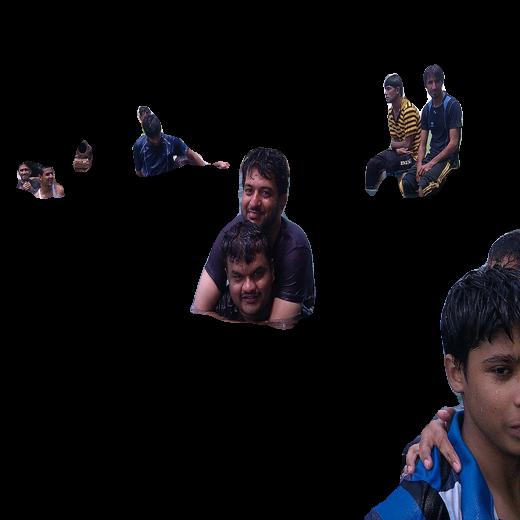

Supplement: Supplementary file 1 — Supplementary Information. [file 41598_2024_80657_MOESM1_ESM.zip › Dataset/High temperature human images/masked_10341861_752157261489641_9201416769670375316_n.jpg]

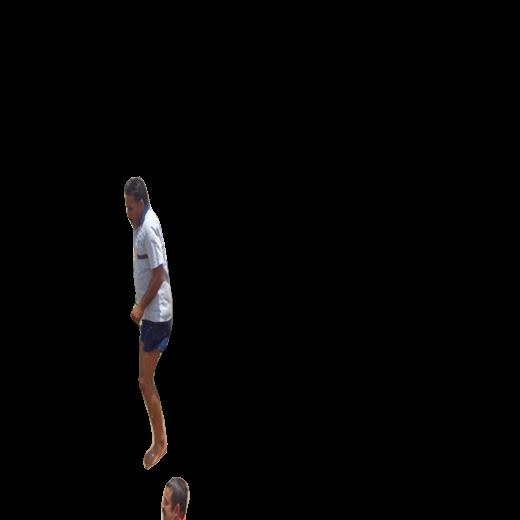

Supplement: Supplementary file 1 — Supplementary Information. [file 41598_2024_80657_MOESM1_ESM.zip › Dataset/High temperature human images/masked_10344420_725950637447991_8129170743696855807_o.jpg]

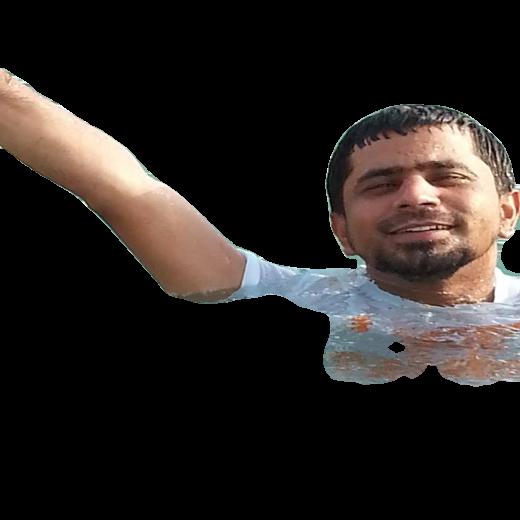

Supplement: Supplementary file 1 — Supplementary Information. [file 41598_2024_80657_MOESM1_ESM.zip › Dataset/High temperature human images/masked_10346188_762433657140644_6769755947242875350_n.jpg]

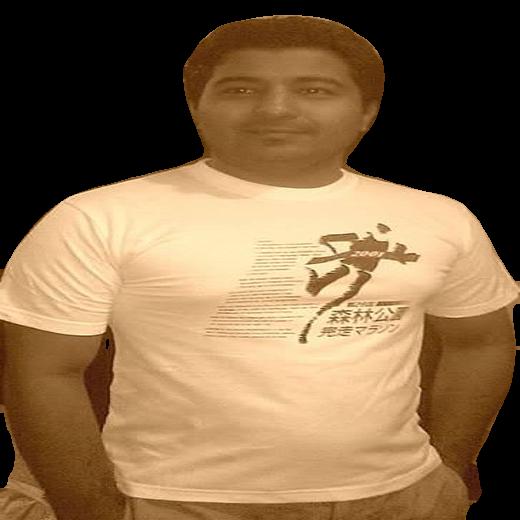

Supplement: Supplementary file 1 — Supplementary Information. [file 41598_2024_80657_MOESM1_ESM.zip › Dataset/High temperature human images/masked_10347509_647106662040237_8868708963654433014_n.jpg]

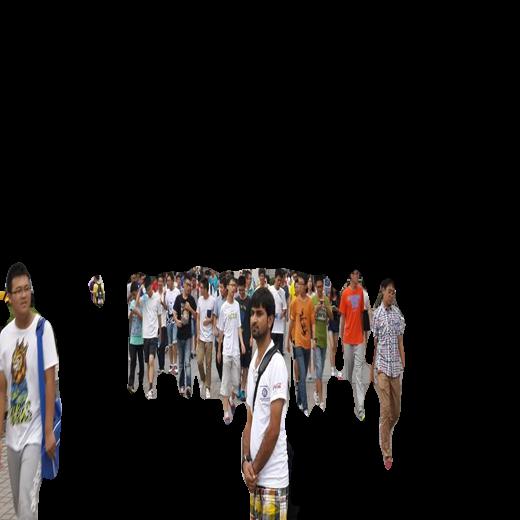

Supplement: Supplementary file 1 — Supplementary Information. [file 41598_2024_80657_MOESM1_ESM.zip › Dataset/High temperature human images/masked_10348442_789748917744295_421570907949046635_n.jpg]
